# Antibacterial Activity of Extract, Fractions, and Compounds from *Termitomyces clypeatus* R. Heim (Lyophyllaceae) Against Multidrug-Resistant Bacteria Overexpressing Efflux Pumps

**DOI:** 10.3390/ph19050737

**Published:** 2026-05-07

**Authors:** Jenifer R. N. Kuete, Jason B. T. Kuete, Joris Baier, Niklas Ehlenz, Simionne L. K. Tonga, Bienvenu Tsakem, Refilwe Matshitse, Borice T. Tsafack, Paul Eckhardt, Beaudelaire K. Ponou, Till Opatz, Léon Azefack Tapondjou, Ilhami Celik, Xavier Siwe-Noundou, Rémy B. Teponno

**Affiliations:** 1 Department of Chemistry, Faculty of Science, University of Dschang, Dschang P.O. Box 67, Cameroon; jeniferkuete@gmail.com (J.R.N.K.); simionnekuitcha@gmail.com (S.L.K.T.); btsakem23@gmail.com (B.T.); boricet@yahoo.fr (B.T.T.); beaudelaireponou@yahoo.fr (B.K.P.); tapondjou2001@yahoo.fr (L.A.T.); 2 Department of Biochemistry, Faculty of Science, University of Dschang, Dschang P.O. Box 67, Cameroon; kuetejason7@gmail.com; 3 Department of Chemistry, Johannes Gutenberg University of Mainz, Duesbergweg 10-14, D-55128 Mainz, Germany; jbaier01@uni-mainz.de (J.B.); ehlenz@uni-mainz.de (N.E.); eckhardt@uni-mainz.de (P.E.); opatz@uni-mainz.de (T.O.); 4 Department of Pharmaceutical Sciences, School of Pharmacy, Sefako Makgatho Health Sciences University, Pretoria 0204, South Africa; refilwemandla@yahoo.com; 5 Department of Chemistry, Faculty of Science, Eskisehir Technical University, Eskisehir 26470, Turkey; ilcelik@eskisehir.edu.tr

**Keywords:** *Termitomyces clypeatus*, antibacterial activity, efflux pumps, antibiotics association

## Abstract

**Background/Objectives**: Microbial resistance to antibiotics has become a major global public health problem, threatening the effectiveness of current therapeutic strategies. The present study seeks to investigate natural compounds originating from fungal sources for their ability to interfere with efflux pump-mediated resistance in multidrug-resistant (MDR) bacteria, with the overarching goal of uncovering new candidates for antimicrobial therapeutic development. A chemical investigation of the ethanol extract of *Termitomyces clypeatus* was carried out to isolate and identify its constituents. **Methods**: Structural elucidation of the isolated metabolites was achieved through 1D and 2D NMR spectroscopy supported by mass spectrometric data. The crude extract and the purified compounds were then evaluated for their antibacterial activities individually, in the presence of an efflux pump inhibitor, and in combination with three antibiotics, using standardized microdilution assays. **Results**: Chromatographic separation of the extract yielded eleven known compounds, including three sphingolipids: (9*Z*,12*Z*)-*N*-(1,3,4-trihydroxyoctadecan-2-yl)octadeca-9,12-dienamide (**1**), 2-hydroxy-*N*-(1,3,4-trihydroxyoctadecan-2-yl)hexadecanamide (**2**), and cerebroside B (**3**); four steroids: ergosterol (**4**), cerevisterol (**5**), ergosterol peroxide (**6**), and 5α,6α-epoxy-(22*E*,24*R*)-ergosta-8(14),22-diene-3*β*,7*α*-diol (**7**); one alkaloid: piperine (**8**); one carbohydrate: D-mannitol (**9**); and two phthalates: dimethyl phthalate (**10**) and bis(2-ethylhexyl) terephthalate (**11**). GC–MS analysis led to the identification of eight fatty acid derivatives (**12**–**19**). Sub-fraction A, along with compounds **3**, **4**, and **8**, exhibited moderate antibacterial activity against some tested strains, with MIC values of 64 μg/mL. These compounds were identified as substrates of bacterial efflux pumps, and their presence enhanced the antibacterial effects of ciprofloxacin, doxycycline, and amikacin. **Conclusions**: The findings of the present work indicate that *Termitomyces clypeatus* contains compounds with potential therapeutic value, as adjuvants that enhance the activity of conventional antibiotics.

## 1. Introduction

Infectious diseases are a global burden, and rising pathogen resistance has substantially reduced the effectiveness of antibiotic therapy [1,2]. In 2019, an estimated 13.7 million deaths were attributed to infectious diseases, of which 7.7 million were linked to bacterial pathogens, encompassing both resistant and susceptible isolates. Multidrug resistance has become a critical public health concern [3,4]. The World Health Organization (WHO) estimates that, if current trends continue, deaths attributable to antimicrobial resistance could rise to 10 million per year by 2050 [5].

With an estimated 60% of the world’s population depending on traditional medicine for primary healthcare, the therapeutic value of natural substances underscores the continued relevance of traditional practices. The use of natural products has gained considerable attention because of their effectiveness, cultural resonance, safety profile, broad availability, and deep roots in ancestral knowledge [6].

*Termitomyces clypeatus*, a wild edible mushroom, is traditionally recommended by several healers for the treatment of various ailments, including measles, yellow fever, and some gastrointestinal infectious diseases [7,8]. Studies have reported that *Termitomyces clypeatus* possesses antioxidant, immunomodulatory, anticancer, antitumor, and antibacterial activities [9]. As part of our ongoing search for potentially bioactive metabolites from African fungi [10,11,12,13,14], we report in the present study the isolation and identification of compounds from the ethanol extract of *Termitomyces clypeatus* as well as their antibacterial potential against multidrug-resistant bacteria.

## 2. Results

### 2.1. Isolation and Identification of Compounds

**Figure 1:**
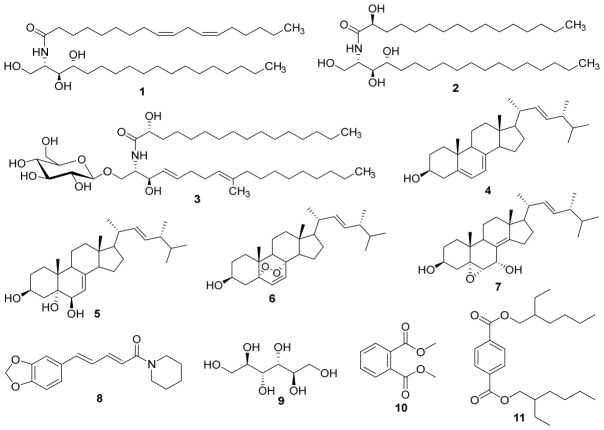
Structures of compounds **1**–**11** isolated from *T. clypeatus*. **1**: (9*Z*,12*Z*)-*N*-(1,3,4-trihydroxyoctadecan-2-yl) octadeca-9,12-dienamide; **2**: 2-hydroxy-*N*-(1,3,4-trihydroxyoctadecan-2-yl) hexadecanamide; **3**: Cerebroside B; **4**: Ergosterol; **5**: Cerevisterol; **6**: Ergosterol peroxide; **7**: 5*α*,6*α*-epoxy-(22*E*,24*R*)-ergosta-8(14),22-diene-3*β*,7*α*-diol; **8**: piperine; **9**: D-mannitol; **10**: Dimethyl phthalate; **11**: Bis (2-ethylhexyl) terephthalate.

The ethyl acetate soluble fraction of the ethanol extract of *T. clypeatus* was subjected to silica gel flash chromatography, and the sub-fractions obtained were repeatedly chromatographed on silica gel and Sephadex LH-20 columns, yielding eleven compounds. The isolated metabolites were identified as: (9*Z*,12*Z*)-*N*-(1,3,4-trihydroxyoctadecan-2-yl)octadeca-9,12-dienamide (**1**), 2-hydroxy-*N*-(1,3,4-trihydroxyoctadecan-2-yl)hexadecanamide (**2**) [15], cerebroside B (**3**) [16], ergosterol (**4**) [12,17], cerevisterol (**5**) [18], ergosterol peroxide (**6**) [19], 5*α*,6*α*-epoxy-(22*E*,24*R*)-ergosta-8(14),22-diene-3*β*,7*α*-diol (**7**) [20], piperine (**8**) [21], D-mannitol (**9**) [22], dimethyl phthalate (**10**) [23], and bis(2-ethylhexyl) terephthalate (**11**) [24] (Figure 1). The spectrometric and spectroscopic data of metabolite **1** match those reported in the literature for ceramide IIIA (*N*-linoleoyl-4-OH-sphinganine), a synthetic compound produced by lipase catalysis, which is an essential constituent in cosmetic formulations and dermatological applications [25]. Compound **2** was previously identified in the hydrolysate formed from the action of acid sphingomyelinase on sphingomyelin by MALDI TOF mass spectrometry [26].

Attempts to isolate compounds from sub-fraction A, the one that enhanced the antibacterial effects of ciprofloxacin, doxycycline, and amikacin, failed. This is the reason why it was analyzed by gas chromatography–mass spectrometry (GC–MS). Eight compounds (**12**–**19**) were identified based on their retention times (RT), molecular formulas, and molecular weights (MW) (Figures S66–S73). The identified compounds include: hexadecanoic acid methyl ester (**12**), hexadecanoic acid ethyl ester (**13**), 9,12-octadecadienoic acid methyl ester (**14**), 9-octadecanoic acid methyl ester (**15**), octadecanoic acid methyl ester (**16**), 9,12-octadecadienoic acid ethyl ester (**17**), 9,12-octadecadienoic acid (**18**), and octadecanoic acid ethyl ester (**19**) (Figures S66–S73).

### 2.2. Antibacterial Activity of Extracts and Compounds

The antibacterial activity of the crude ethanolic extract, the ethyl acetate and *n*-butanol fractions, sub-fraction A, and several isolated compounds was evaluated against ten MDR Gram-negative bacteria, including *Pseudomonas aeruginosa*, *Klebsiella pneumoniae*, *Escherichia coli*, *Providencia stuartii*, and *Enterobacter aerogenes*. The MIC and MBC values are displayed in Table 1, Table 2 and Table 3. The crude extract and the ethyl acetate as well as the *n*-butanol fractions showed no remarkable activity against the tested strains. In contrast, sub-fraction A derived from the ethyl acetate fraction exhibited moderate antibacterial activity, with MIC values ranging from 32 to 256 µg/mL against 50% of the tested bacteria. It demonstrated good activity against *Klebsiella pneumoniae* KP55 (MIC = 64 µg/mL) and modest activity against *Pseudomonas aeruginosa* (PA01, PA124) and *Providencia stuartii* P2636 (MIC = 128 µg/mL).

Among the isolated metabolites, compounds **4**–**7** showed moderate to low activity against the tested microorganisms. Compounds **3**, **4**, and **5** displayed moderate activity against *Escherichia coli* ATCC 10536, with MICs of 64 µg/mL for compounds **3** and **4**, and 32 µg/mL for compound **5**, while exhibiting low activity (MIC 128–256 µg/mL) against other strains. Compound **9** demonstrated moderate to low activity, with the best inhibition observed against *Enterobacter aerogenes* EA3 (MIC = 64 µg/mL). Compound **10** showed no significant antibacterial activity against the tested strains. Compounds **1** and **2** were not tested because they showed very low solubility in DMSO. Since DMSO is a polar solvent, the poor solubility could be explained by the lipophilic nature of these two ceramides.

### 2.3. Effect of Samples in Presence of Efflux Pumps Inhibitors

To evaluate whether the test samples act as substrates or inhibitors of bacterial efflux pumps, the MIC values of selected compounds were determined in the presence of the efflux pump inhibitor PA*β*N against the same Gram-negative bacteria (Table 4). No significant change in activity was observed for sub-fraction A in the presence of PA*β*N. However, the antibacterial activity of compound **4** improved against 50% of the tested strains, with an AIF of up to 8 against *Klebsiella pneumoniae* KP55 and *Enterobacter aerogenes* EA27. Similarly, the activity of compound **3** increased against 60% of the bacteria, with an AIF of up to 64 against *Enterobacter aerogenes* EA3. Compound **9** also showed enhanced activity against 50% of the strains, with a maximum improvement factor of 32 against *Escherichia coli* ATCC 10536 and *Providencia stuartii* PS2636.

### 2.4. Determination of the Antibiotic-Potentiating Effects of the Samples

To determine the suitable concentrations of samples for combination studies with antibiotics, a preliminary assay was conducted on *Escherichia coli* AG100. Sub-fractions A, B, C, and D, as well as compounds **3**, **4**, and **8**, showed the greatest potentiation of antibiotic activity at sub-inhibitory concentrations (MIC/2 and MIC/4). Based on these results, the selected samples were further tested in combination with antibiotics at MIC/2 and MIC/4. The results are summarized in Table 5 and Table 6.

All the sub-fractions enhanced the activity of the tested antibiotics. For ciprofloxacin, sub-fractions B-D achieved 100% potentiation across all tested bacteria, reducing the MIC from 512 to 4 µg/mL. For doxycycline, sub-fraction B was the most effective, producing 80% potentiation with a maximum improvement factor of 16 against *Pseudomonas aeruginosa* PA124 and *Enterobacter aerogenes* EA3 at both MIC/2 and MIC/4. Amikacin showed a more modest enhancement, with sub-fraction B increasing its activity by 40%, achieving an improvement factor of 8 against *Pseudomonas aeruginosa* PA124.

The isolated compounds exhibited variable effects. The activity of ciprofloxacin was enhanced by up to 40%, with an improvement factor of 2 at MIC/2 in combination with compound **4** against *Providencia stuartii* PS2636. The activity of doxycycline was potentiated by 40%, with an improvement factor of 16 against *Enterobacter aerogenes* when combined with compound **8**. However, the activity of amikacin increased by 20% in the presence of compound **4**, with an improvement factor of 2 at both MIC/2 and MIC/4 against *Pseudomonas aeruginosa* PA124.

## 3. Discussion

The chemical investigation of *T. clypeatus* led to the isolation and identification of 19 compounds, and we noticed that similar metabolites have been characterized in fungi of the genus *Termitomyces* [27]. Cerebroside B (**3**) was previously from *T. albuminosus* [27] and was recently identified by LC-MS/MS from *T. clypeatus* [28,29]. However, further chemical studies on species of the genus *Termitomyces* are needed to determine whether this compound can be considered as a chemotaxonomic marker. It is important to point out that the steroids cerevisterol (**5**), ergosterol peroxide (**6**), and 5α,6α-epoxy-(22*E*,24*R*)-ergosta-8(14),22-diene-3β,7α-diol (**7**) were already isolated from a *Termitomyces* species, notably *T. microcarpus* [30], while ergosterol was obtained from *T. heimii* [31,32]. Piperine is the main constituent of the medicinal plant *Piper nigrum* that has gained the attention of the chemistry and physiology communities mostly due to its wide range of biological activities [33]. It was also shown to be produced by endophytic fungi *Colletotrichum gloeosporioides* [34] and *Periconia* sp. [35], harboured in *Piper nigrum* and *Piper longum*, respectively. Several synthetic routes for this compound have also been developed [33]. Compounds **10** and **11** are phthalates and are generally considered as contaminants derived from plastics. Although some have been reported from natural sources including plants, fungi, and bacteria [36], these findings should be interpreted with caution, as the samples or solvents used for their extraction and purification may have been contaminated with synthetic compounds. However, the biosynthesis of phthalates in filamentous fungi has been investigated, and it has been reported that through the shikimic acid pathway, D-glucose can be converted into phthalic acid, which is subsequently esterified to afford the corresponding phthalates [37]. Importantly, no specific contamination control experiments (e.g., solvent or procedural blanks, or exclusion of plastic labware) were performed in the present study; therefore, a contamination origin of these compounds cannot be excluded and should be acknowledged as a limitation.

The antibacterial assays of the extracts, fractions and sub-fractions from *T. clypeatus* revealed that sub-fraction A exhibited higher activity than the crude extract and the EtOAc fraction. This enhanced activity may be attributed to several factors, primarily the removal of interfering matrix components such as pigments, tannins, and lipids, which can reduce the solubility and bioavailability of the crude extract [38]. Secondly, the fractionation process enables the isolation of individual compounds from the crude extract, thereby reducing potential inhibitory or antagonistic interactions among constituents [39]. Finally, sub-fraction A may contain a higher proportion of bioactive compounds, contributing to its enhanced antibacterial activity [40]. The antibacterial activity of compound **3** is consistent, and this is in accordance with previous studies which showed that cerebrosides exhibit notable activity against *Escherichia coli* strains [41]. Compounds **4**–**7** are steroid derivatives, and metabolite **4** showed the highest antibacterial activity. This difference may be explained by the higher polarity of compounds **5**, **6** and **7**, which likely hinders their ability to penetrate the outer membrane of Gram-negative bacteria. Gram-negative bacteria possess an outer membrane rich in lipopolysaccharides, which serves as a barrier to the entry and diffusion of polar molecules [42]. The antibacterial activity of compound **8** is also consistent with previously reported findings [43]. Compound **9**, in contrast, exhibited no significant antibacterial activity against the tested strains; however, the literature reports indicate that it may exert indirect effects by inhibiting bacterial biofilm formation [44]. These findings confirm the antibacterial potential of both the extracts and the isolated compounds from *T. clypeatus*.

Since active efflux is the main resistance mechanism in Gram-negative bacteria, we evaluated the antibacterial activity of sub-fractions and some isolated compounds in the presence of the efflux pump inhibitor PA*β*N. The contribution of efflux pumps was inferred by comparing MIC values obtained with and without PAβN. A reduction in MIC (AIF > 1) indicates that the compound is at least partially extruded by efflux systems; inhibition of these pumps therefore increases its intracellular accumulation. The high AIF values observed for compounds **3**, **4**, and **9** strongly suggest that they behave primarily as efflux substrates. In contrast, a true efflux pump inhibitor typically displays limited intrinsic antibacterial activity but consistently lowers the MICs of multiple unrelated antibiotics that are known efflux substrates. Because our samples exhibited pronounced AIF responses together with antibiotic-potentiating effects, the data support the conclusion that these metabolites function mainly as efflux substrates, whose antibacterial and adjuvant activities are modulated by efflux, rather than as direct efflux pump inhibitors. It is also important to note that PAβN has dual activity: beyond inhibiting Resistance-Nodulation-Division (RND) efflux pumps, it can increase outer-membrane permeability, thereby facilitating the entry of compounds into the periplasm and cytoplasm. The absence of activity enhancement in some cases may reflect the involvement of alternative efflux systems that are not inhibited by PAβN [45]. Additionally, hydrophobic molecules are frequently substrates of RND pumps such as AcrAB-TolC in *Escherichia coli* and MexAB-OprM in *Pseudomonas aeruginosa* [46]. If such molecules fail to efficiently cross the inner membrane and remain confined to the periplasmic space, efflux inhibition alone may not be sufficient to increase their intracellular concentration, resulting in limited antibacterial activity [47,48].

The antibiotic potentiating effect of some sub-fractions and isolated compounds was evaluated since it is known that the association of antibiotics with natural products is an alternative in the fight against multidrug-resistant bacteria. Sub-fractions B-D showed considerable antibiotic potentiating effects, probably due to the bioactive metabolites they contain and this was more pronounced for ciprofloxacin (100% potentiation) (Table 5). For pure isolated metabolites, some notable synergistic effects were observed for cerebroside B (**3**), ergosterol (**4**), and piperine (**8**) when they were associated with ciprofloxacin, doxycycline, and amikacin (Table 6). Piperine has already shown to considerably reduce the MICs of ciprofloxacin for some multidrug-resistant *Staphylococcus aureus* strains [49], and more recently, it was demonstrated that this compound is capable of improving the effectiveness of rifampicin and tetracycline [50]. The antimicrobial activity of ergosterol is well documented [51] and its antibiotic modulating effect for aminoglycosides was also reported [47]. Thus, the presence of these metabolites in active sub-fractions may be the origin of the observed antibiotic-potentiating activity.

## 4. Materials and Methods

### 4.1. General Experimental Procedures

The high-resolution mass spectra were recorded on an Agilent 6545 QTOF-MS spectrometer (Agilent GmbH, Waldbronn, Germany) equipped with a HRESI source, a LockSpray interface, and a suitable external calibrant. LC-MS spectra were obtained using a 1260 Infinity HPLC-System by Agilent Technologies coupled to a Quadrupole-ESI-MS (G612B, Agilent InfinityLab LC/MSD Series, Agilent, Santa Clara, CA, USA). The GC-MS was performed on a Shimadzu Ultra Model QP-2010 GC (Shimadzu, Kyoto, Japan) coupled with MS. The GC was equipped with a capillary column (30 m × 0.25 mm i.d., film thickness 0.25 μm) and HP-5 MS (5% phenylmethyl siloxane) at a helium flow rate of 1.61 mL/minute with the temperature ranging from 40 °C to 280 °C for 10 min at a rate of 20 °C/min. The ion source was maintained at 250 °C and 70 eV electron energy. Methanol was added to the extracts before injecting 1 μL into the column. The exact name and molecular weight of unknown compounds were found by comparing their mass spectrum with the reference spectra available in the GC/MS Library. NMR spectra were recorded in deuterated solvents (acetone-*d*_6_, CD_3_OD, DMSO-*d*_6_, and pyridine-*d*_5_) on a Bruker Avance-III (^1^H NMR, 600 MHz; ^13^C NMR, 150.9 MHz) spectrometer (Bruker, Rheinstetten, Germany) equipped with a 5 mm TCI cryoprobe. All chemical shifts (*δ*) are reported in ppm relative to the residual solvent signals and coupling constants (*J*) are given in Hz. Column chromatography was performed using silica gel 60 (Merck, Darmstadt/Germany) (0.063–0.200 mm and 0.04–0.063 mm) and Sephadex LH-20. The following solvent systems were used: MeOH for Sephadex column chromatography and mixtures of hexane-EtOAc and EtOAc-MeOH for silica gel column chromatography. Thin-layer chromatography (TLC) was performed on Merck precoated silica gel 60 F_254_ aluminum foil. The plates were revealed using a UV lamp (254–365 nm) and 10% H_2_SO_4_ reagent followed by heating at 90 °C.

The flash chromatography was used during the initial separation of the EtOAc and *n*-BuOH fractions, using the Büchi column (460 × 25 mm) and silica gel GF254. Once filled with the stationary phase and the fixed extract, the column was connected to a vacuum pump, Büchi V-700 (Büchi, Flawil, Switzerland), and the elution system solvent consisted of *n*-hexane-EtOAc and EtOAc-MeOH mixtures in increasing polarity.

### 4.2. Fungal Material

The mushroom *Termitomyces clypeatus* R. Heim (Lyophyllaceae) was collected in Banengo-Bafoussam (5°27′34.5″ N 10°25′05.4″ E, Western Region of Cameroon) in October 2021 and identified by Professor Njouonkou André-Ledoux, mycologist at the University of Bamenda.

### 4.3. Extraction and Isolation

The air-dried and pulverized fungal material (*T. clypeatus*) (6 kg) was macerated in ethanol 95% (15 L) at 25 °C for 24 h (3 times) to give, after evaporation to dryness, 725.76 g of crude extract. An amount of 722.51 g of this extract was suspended in distilled water (500 mL) and successively partitioned with EtOAc (3 × 500 mL) and *n*-BuOH (3 × 500 mL). The solutions were evaporated under reduced pressure to afford 344.42 g and 24.32 g of each fraction, respectively. A part of the EtOAc fraction (341.67 g) was subjected to silica gel (0.2–0.5 mm) flash chromatography eluted from *n*-hexane–EtOAc 5% to EtOAc–MeOH 20% to give five main sub-fractions (A–E). The different sub-fractions collected were grouped according to the elution solvents: A [101.42 g, *n*-hexane-EtOAc 95:5 (*v*:*v*)], B [16.46 g, *n*-hexane-EtOAc 80:20 (*v*:*v*)], C [16.80 g, *n*-hexane-EtOAc 40:60 (*v*:*v*)], D [13.48 g, *n*-hexane-EtOAc 0:100 (*v*:*v*)], and E [4.39 g, EtOAc-MeOH 90:10 (*v*:*v*)]. The sub-fraction A (101.42 g) was oily yellow and was qualitatively investigated using the GC-MS technique to determine its constituents. To identify the exact name and molecular weight of the different compounds, the spectra obtained were compared to the Wiley GC/MS Library, Mass Finder Library, and Adams Library. Compounds **12**–**19** were identified.

From sub-fraction B (16.46 g), white flakes were recrystallized with MeOH to afford compound **4** (520 mg). The filtrate was further subjected to the silica gel CC (0.063–0.200 mm) eluted with *n*-hexane–EtOAc (90:10 to 70:30) to afford six main sub-fractions: B1 [2.30 g, *n*-hexane-EtOAc 90:10], B2 [4.86 g, *n*-hexane-EtOAc 90:10 to 85:15], B3 [1.60 g, *n*-hexane-EtOAc 85:15], B4 [0.9 g, *n*-hexane-EtOAc 85:15], B5 [2.80 g, *n*-hexane-EtOAc 80:20], and B6 [1.20 g, *n*-hexane-EtOAc 70:30]. The sub-fraction B2 (4.80 g) was subjected to silica gel CC using *n*-hexane–EtOAc (82:18) to afford compounds **4** (9 mg) and **6** (13 mg). The filtration of sub-fraction C (23.80 g) afforded compound **1** (240 mg) and the filtrate obtained was purified by silica gel CC eluted with *n*-hexane–EtOAc (80:20 to 60:40) to yield five sub-fractions based on comparative TLC: C1 [3.94 g, *n*-hexane-EtOAc 80:20], C2 [7.38 g, *n*-hexane-EtOAc 80:20, 75:25], C3 [4.91 g, *n*-hexane-EtOAc 75:25], C4 [1.60 g, *n*-hexane-EtOAc 70:30], and C5 [0.35 g, *n*-hexane-EtOAc 60:40]. The sub-fraction C1 was filtered and washed with *n*-hexane-EtOAc 40:60 to give compound **2** (27 mg) as a white powder. Sub-fraction C3 was subjected to the silica gel CC using *n*-hexane–EtOAc (75:25) to yield compounds **5** (10 mg) and **7** (20 mg) while sub-fraction C4 was subjected to the silica gel CC using *n*-hexane–EtOAc (78:22) to afford compound **11** (3 mg). Compounds **10** (10 mg) and **8** (91 mg) were obtained from the sub-fraction C5 by Sephadex LH-20 CC [CH_2_Cl_2_-MeOH 1:1 (*v*/*v*)]. Sub-fraction E (4.39 g) was purified by silica gel CC using *n*-hexane–EtOAc (15:85) to EtOAc–MeOH (85:15) to afford five main sub-fractions: E1 [0.58 g, *n*-hexane-EtOAc 15:85], E2 [1.03 g, *n*-hexane-EtOAc 00:100], E3 [1.54 g, EtOAc–MeOH 95:5], E4 [0.31 g, EtOAc–MeOH 90:10], and E5 [0.18 g, EtOAc–MeOH 85:15]. Compound **3** (200 mg) crystallized from sub-fraction E2 while compound **9** (18 mg) crystallized in sub-fractions E5.

*(9Z,12Z)-N-(1,3,4-trihydroxyoctadecan-2-yl)octadeca-9,12-dienamide (**1**)*: White powder; Molecular formula: C_36_H_69_NO_4_; HR-ESI (-) *m*/*z* 624.5200 [M + HCOO]^−^ (cald for C_37_H_70_NO_6_^−^: 624,5209), HR-ESI (+) *m*/*z* 580.5291 [M + H]^+^ (cald for C_36_H_70_NO_4_^+^: 580.5299); ^1^H NMR (600 MHz, DMSO-*d*_6_) *δ* 7.50 (d, *J* = 8.9 Hz, 1H, NH), 5.32 (m, 2H, H-9′/H-13′), 5.30 (m, 2H, H-10′/H-12′), 4.54 (s, 1H, OH-3), 4.46 (s, 1H, OH-1), 4.21 (s, 1H, OH-4), 3.79 (m, 1H, H-2), 3.48 (m, 1H, H-1a); 3.45 (m, 1H, H-1b), 3.36 (m, 1H, H-3), 3.31 (m, 1H, H-4), 2.71 (t, *J* = 6.9 Hz, 2H, H-11′), 2.03 (d, *J* = 9.6 Hz, 2H, H-2′), 1.99 (m, 4H, H-8′/H-14′), 1.41 (m, 2H, H-3′), 0.83 (t, *J* = 6.7 Hz, 6H, Me-18/Me-18′); ^13^C NMR (150 MHz, DMSO-*d*_6_) *δ* 172.3 (C-1′), 130.2 (C-9′/C-13′), 128.2 (C-10′/C-12′), 74.6 (C-3), 71.2 (C-4), 61.1 (C-1), 52.5 (C-2), 36.0 (C-2′), 31.4 (C-16′), 29.9–29.0 (C-5 to C-17, C-4′ to C-7′, C-15′), 27.2 (C-8′/C-14′), 25.8 (C-3′), 25.7 (C-11′), 22.6 (C-17′), 14.4 (C-18/C-18′).

*2-hydroxy-N-(1,3,4-trihydroxyoctadecan-2-yl)hexadecanamide (**2**)*: White powder; Molecular formula: C_34_H_69_NO_5_; LC-ESI-MS *m*/*z* 592.30 [M + K-H_2_O]^+^, *m*/*z* 429.40 [M − C_13_H_27_ + Na − H_2_O]^+^, *m*/*z* 383.35 [M − C_15_H_31_O + K]^+^, *m*/*z* 325.00 [M − C_16_H_32_NO_2_ + Na]^+^; ^1^H NMR (600 MHz, Pyridine-*d*_5_) *δ* 5.15 (m, 1H, H-2), 4.65 (dd, *J* = 7.9, 3.7 Hz, 1H, H-2′), 4.53 (dd, *J* = 11.0, 4.8 Hz, 1H, H-1a), 4.45 (dd, *J* = 10.9, 4.8 Hz, 1H, H-1b), 4.40 (t, *J* = 5.7 Hz, 1H, H-3), 4.30 (t, *J* = 7.9 Hz, 1H, H-4), 2.26 (m, 1H, H-5a), 2.23 (m, 1H, H-3′a), 2.04 (m, 1H, H-3′b), 194 (m, 1H, H-5b), 1.92 (m, 2H, H-6), 1.69 (m, 2 H, H-4′), 0.85 (o, 6H, H-18/H-16′), ^13^C NMR (150 MHz, Pyridine-*d*_5_) *δ* 175.2 (C-1′), 76.4 (C-3), 72.7 (C-2′), 72.2 (C-4), 61.7 (C-1), 52.7 (C-2), 35.4 (C-3′), 33.8 (C-5), 29.9–29.4 (C-7 to C-16, C5′ to C15′), 26.4 (C-6), 25.6 (C-4′), 22.7 (C-17), 14.1 (C-18/C-16′).

*Cerebroside B (**3**)*: White powder; Molecular formula: C_41_H_78_NO_9_; LC-ESI-MS *m*/*z* 728.200 [M + H]^+^; ^1^H-NMR (CD_3_OD, 600 MHz): *δ* 5.75 (m, 1H, H-5), 5.51 (dd, *J* = 15.5, 7.4 Hz, 1H, H-4), 5.16 (tt, *J* = 5.5, 2.4, 1H, H-8), 4.29 (d, *J* = 7.8 Hz, 1H, H-1″), 4.15 (o, 2H, H-1a/H-3), 4.01 (m, 2H, H-2/2′), 3.89 (m, 1 H, H-6″a), 3.72 (dd, *J* = 6.7, 5.5 Hz, 1H, H-1b), 3.69 (m, 1 H, H-6″b), 3.37 (m, 1 H, H-3″), 3.30 (m, 1H, H-4″), 3.29 (m, 1H, H-5″), 3.21 (dd, *J* = 9.2, 7.8 Hz, 1H, H-2″), 2.10 (m, 2H, H-7), 2.07 (m, 2H, H-6), 2.00 (t, *J* = 7.6, 2H, H-10), 1.72 (t, *J* = 1.3 Hz, 1H, H-3′a), 1.61 (d, *J* = 1.3 Hz, 3H, H-19), 1.57 (t, *J* = 1.3 Hz, 1H, H-3′b), 1.43 (m, 2H, H-4′), 0.92 (t, *J* = 6.9 Hz, 6H, Me-18 and Me-16′); ^13^C-NMR (CD_3_OD, 150 MHz): *δ* 175.8 (C-1′), 135.3 (C-9), 133.2 (C-5), 129.7 (C-4), 123.4 (C-8), 103.3 (C-1″), 76.6 (C-5″), 76.4 (C-3″), 73.6 (C-2″), 71.6 (C-2′), 71.4 (C-3), 70.1 (C-4″), 68.3 (C-1), 61.2 (C-6″), 53.3 (C-2), 39.4 (C-10), 37.3 (C-7), 34.5 (C-3′), 32.4 (C-6), 29.4–27.7 (C-11 to C-17/C-5′ to C-15′), 24.8 (C-4′), 14.7 (C-19), 13.1 (C-18/C-16).

*Ergosterol (**4**)*: White powder; ^1^H-NMR (CDCl_3_, 600 MHz): *δ* 5.59 (dd, *J* = 5.7, 2.5 Hz, 1H, H-6), 5.40 (dd, *J* = 5.6, 2.8 Hz, 1H, H-7), 5.23 (m, 1H, H-23), 5.20 (m, 1H, H-22), 3.65 (m, 1H, H-3), 2.48 (m, 1H, H-4a), 2.30 (m, 1H, H-4b), 2.09 (dd, *J* = 4.8, 2.5, 1H, H-12), 2.05 (m, 1H, H-20), 1.99 (d, *J* = 2.3 Hz, 1H, H-9), 1.91 (m, 2H, H-1), 1.90 (d, *J* = 2.3 Hz, 1H, H-14), 1.89 (m, 1H, H-2a), 1.87 (m, 1H, H-24), 1.71 (m, 1H, H-11), 1.68 (m, 2H, H-15), 1.59 (m, 1H, H-2b), 1.49 (m, 1H, H-25), 1.32 (m, 2H, H-16), 1.27(m, 1H, H-17), 1.05 (d, *J* = 6.6 Hz, 3H, Me-21), 0.96 (s, 3H, Me-18), 0.93 (d, *J* = 6.8 Hz, 3H, Me-28), 0.85 (d, *J* = 6.8 Hz, 6H, Me-26/Me-27), 0.65 (s, 3H, Me-19), ^13^C-NMR (CDCl_3_, 150 MHz): *δ* 141.4 (C-8), 139.8 (C-5), 135.6 (C-22), 132.0 (C-23), 119.6 (C-6), 116.3 (C-7), 70.5 (C-3), 55.7 (C-17), 54.5 (C-14), 46.2 (C-9), 42.8 (C-24/13), 40.7 (C-4), 40.5 (C-20), 39.1 (C-12), 38.4 (C-1), 37.0 (C-10), 33.1 (C-25), 32.0 (C-2), 28.3 (C-16), 23.0 (C-15), 21.1 (C-11/C-21), 20.0 (C-26), 19.7 (C-27), 17.6 (C-28), 16.3 (C-19), 12.1 (C-18).

*Cerevisterol (**5**)*: White powder; ^1^H-NMR (CDCl_3_, 600 MHz): *δ* 5.24 (dd, *J* = 15.3, 7.4 Hz, 1H, H-23), 5.17 (dd, *J* = 15.3, 8.3 Hz, 1H, H-22), 5.08 (dt, *J* = 5.0, 2.3 Hz, 1H, H-7), 3.76 (tq, *J* = 10.7, 5.1 Hz, 1H, H-3), 3.37 (m, 1H, H-6), 2.00 (m, 1H, H-20), 1.96 (m, 1H, H-12a), 1.93 (m, 1H, H-9), 1.89 (m, 1H, H-4a), 1.85 (m, 1H, H-24), 1.80 (m, 1H, H-14), 1.67 (m, 1H, H-16), 1.61 (m, 1H, H-2a), 1.50 (m, 1H, H-4b), 1.48 (m, 2H, H-15), 1.46 (m, 1H, H-25), 1.44 (m, 1H, H-11a), 1.40 (m, 1H, H-11b), 1.30 (m, 1H, H-1), 1.25 (m, 1H, H-12b), 1.26 (m, 1H, H-17), 1.23 (m, 1H, H-2b), 1.00 (d, *J* = 6.6 Hz, 3H, H-21), 0.90 (s, 3H, H-19), 0.89 (d, *J* = 6.9 Hz, 3H, H-28) 0.81 (d, *J* = 6.4 Hz, 6H, H-26/H-27),0.55 (s, 3H, H-18); ^13^C-NMR (CDCl_3_, 150 MHz): *δ* 140.1 (C-8), 135.8 (C-22), 132.3 (C-23),119.9 (C-7), 74.4 (C-5), 72.5 (C-6), 66.4 (C-3), 55.7 (C-17), 54.6 (C-14), 43.4 (C-13), 42.7 (C-9), 42.4 (C-24), 40.6 (C-4), 40.4 (C-20), 39.5 (C-12), 37.1 (C-10), 32.90 (C-1/C-25), 31.6 (C-2), 28.2 (C-16), 23.0 (C-15), 21.7 (C-11), 21.4 (C-21), 19.9 (C-26), 19.6 (C-27), 18.1 (C-19), 17.7 (C-28), 12.5 (C-18).

*Ergosterol peroxyde (**6**)*: White powder; ^1^H-NMR (CDCl_3_, 600 MHz): *δ* 6.53 (d, *J* = 8.5 Hz, 1H, H-7), 6.26 (d, *J* = 8.5 Hz, 1H, H-6), 5.24 (dd, *J* = 15.2, 7.7 Hz, 1H, H-23), 5.16 (dd, *J* = 15.4, 8.3 Hz, 1H, H-22), 3.99 (tt, *J* = 11.5, 5.1 Hz, 1H, H-3), 2.14 (m, 1H, H-4a), 2.13 (ddd, *J* = 13.8, 5.0, 2.0 Hz, 1H, H-10), 2.03 (m, 1H, H-20), 1.97 (m, 1H, H-12a), 1.96 (m, 1H, H-1a), 1.94 (m, 1H, H-4b), 1.87 (m, 1H, H-24), 1.85 (m, 1H, H-2a), 1.72 (m, 1H, H-1b), 1.60 (m, 1H, H-11a), 1.59 (m, 1H, H-9), 1.58 (d; J = 4.0 Hz, 1H, H-14), 1.56 (m, 1H, H-2b), 1.53 (m, 1H, H-15a), 1.48 (m, 1H, H-25), 1.43 (m, 1H, H-11b), 1.36 (m, 2H, H-16), 1.24 (m, 1H, H-12b/H-15b), 1.23 (m, 1H, H-17), 1.02 (d, *J* = 6.6 Hz, 3H, Me-21), 0.93 (d, *J* = 6.9 Hz, 3H, Me-28), 0.90 (s, 3H, Me-19), 0.86 (s, 3H, Me-18), 0.84 (d, *J* = 6.4 Hz, 6H, Me-26 and Me-27); ^13^C-NMR (CDCl_3_, 150 MHz): *δ* 135.4 (C-6), 135.2 (C-22), 132.3 (C-23), 130.8 (C-7), 82.2 (C-5), 79.4 (C-8), 66.5 (C-3), 56.1 (C-17), 51.6 (C-14), 51.0 (C-9), 44.5 (C-13), 42.8 (C-24), 39.8 (C-20), 20.9 (C-21), 39.3 (C-12), 36.9 (C-4/C-10), 34.7 (C-1), 33.1 (C-25), 30.1 (C-2), 28.7 (C-16), 23.4 (C-15), 20.6 (C-11), 19.9 (C-26), 19.6 (C-27), 18.2 (C-19), 17.6 (C-28), 12.8 (C-18).

*5α,6α-epoxy-(22E,24R)-ergosta-8(14),22-diene-3β,7α-diol (**7**)*: White powder; ^1^H-NMR (CD_3_OD, 600 MHz): *δ* 5.25 (m, 2H, H-22/H-23), 4.43 (d, *J* = 3.4 Hz, 1H, H-7), 3.77 (tt, *J* = 11.3, 4.6 Hz, 1H, H-3), 3.08 (d, *J* = 3.4 Hz, 1H, H-6), 2.66 (m, 1H, H-15a), 2.45 (ddd, *J* = 10.5, 7.0, 3.4 Hz, 1H, H-9), 2.26 (m, 1H, H-15b), 2.00 (m, 1H, H-20), 1.96 (m, 1H, H-12a), 1.91 (m, 1H, H-2a), 1.87 (m, 1H, H-24), 1.75 (m, 1H, H-16a), 1.65 (m, 1H, H-4a), 1.63 (m, 1H, H-1a), 1.56 (m, 1H, H-2b), 1.55 (m, 1H, H-4b), 1.52 (m, 1H, H-11a), 1.49 (m, 1H, H-25), 1.46 (m, 1H, H-11b), 1.45 (m, 1H, H-16b), 1.43 (m, 1H, H-1b), 1.25 (m, 1H, H-17), 1.23 (m, 1H, H-12b), 1.05 (d, *J* = 6.5, 3H, Me-21), 0.96 (d, *J* = 6.8, 3H, Me-28), 0.92 (s, 3H, Me-18), 0.90 (s, 3H, Me-19), 0.88 (d, *J* = 6.8, 3H, Me-26), 0.86 (d, *J* = 6.8, 3H, Me-27); ^13^C-NMR (CD_3_OD, 150 MHz): *δ* 151.4 (C-14), 135.5 (C-22), 131.9 (C-23), 125.1 (C-8), 67.8 (C-3), 66.7 (C-5), 64.4 (C-7), 61.2 (C-6), 56.7 (C-17), 42.9 (C-24), 42.7 (C-13), 40.7 (C-4), 39.3 (C-20), 39.1 (C-9), 36.4 (C-12), 35.6 (C-10), 32.9 (C-25), 32.0 (C-1), 30.4 (C-2), 27.0 (C-16), 24.2 (C-15), 20.4 (C-21), 19.1 (C-26), 18.8 (C-11), 18.7 (C-27), 17.1 (C-18), 16.8 (C-28), 15.4 (C-19).

*Piperine (**8**):* Yellow oil; ^1^H-NMR (CD_3_OD, 600 MHz): *δ* 7.32 (dd, *J* = 14.6, 10.7 Hz, 1H, H-3), 7.10 (d, *J* = 1.7 Hz, 1H, H-7), 6.96 (dd, *J* = 8.1, 1.7 Hz, 1H, H-12), 6.89 (m, 1H, H-4), 6.84 (s, 1H, H-5), 6.80 (d, *J* = 8.0 Hz, 1H, H-11), 6.63 (d, *J* = 14.6 Hz, 1H, H-2), 5.97 (s, 2H, H-9), 3.64 (m, 4H, H-13/H-17), 1.70 (qd, *J* = 6.0, 5.1 Hz, 2H, H-15), 1.60 (m, 4H, H-14/H-16); ^13^C-NMR (CD_3_OD, 150 MHz): *δ* 167.7 (C-1), 149.8 (C-8), 149.7 (C-10), 144.6 (C-3), 140.2 (C-5), 132.4 (C-6), 126.4 (C-4), 123.9 (C-12), 120.6 (C-2), 109.4 (C-11), 106.7 (C-7), 102.7 (C-9), 48.1 (C-17), 44.5 (C-13), 27.8 (C-14), 26.9 (C-16), 25.6 (C-15).

*D-mannitol (**9**)*: White powder; ^1^H-NMR (CD_3_OD, 600 MHz): *δ* 3.83 (dd, *J* = 11.1, 3.6 Hz, 2H, H-1a), 3.79 (d, *J* = 8.1 Hz, 2H, H-3), 3.71 (ddd, *J* = 8.1, 6.0, 3.6 Hz, 2H, H-2), 3.65 (dd, *J* = 11.2, 6.0 Hz, 2H, H-1b); ^13^C-NMR (CD_3_OD, 150 MHz): *δ* 71.5 (C-2), 69.8 (C-3), 63.7 (C-1).

*Dimethyl phthalate (**10**)*: Yellow oil; ^1^H-NMR (CD_3_OD, 600 MHz): *δ* 7.75 (dd, *J* = 5.7, 3.3 Hz, 2H, H-3/H-4), 7.64 (dd, *J* = 5.7, 3.3 Hz, 2H, H-2/H-5), 3.90 (s, 6H, Me-2′/Me-2″); ^13^C-NMR (CD_3_OD, 150 MHz): *δ* 169.6 (C-1′/C-1″), 133.3 (C-1/C-6), 132.5 (C-2/C-5), 129.9 (C-3/C-4), 53.2 (C-2′/C-2″).

*Bis (2-ethylhexyl) terephthalate (**11**)*: White powder; ^1^H-NMR (CD_3_OD, 600 MHz): *δ* 8.07 (s, 4H, H-2, H-3, H-5, H-6), 4.25 (dd, *J* = 5.7, 2.7 Hz, 4H, H-8/H-8′), 1.72 (m, 2H, H-9/H-9′), 1.50 (m, 4H, H-12/12′), 1.46 (m, 4H, H-10/10′), 1.34 (m, 4H, H-14/14′), 1.31 (m, 4H, H-11/11′), 0.93 (t, *J* = 7.5 Hz, 6H, H-13/13′), 0.87 (dt, *J* = 15.3, 7.1 Hz, 6H, H-15/15′); ^13^C-NMR (CD_3_OD, 150 MHz): *δ* 166.7 (C-7/7′), 135.1 (C-1/4), 130.2 (C-2/C-3/C-5/C-6), 68.2 (C-8/8′), 39.9 (C-9/9′), 31.3 (C-10/10′), 29.7 (C-11/11′), 24.6 (C-12/12′), 23.3 (C-14/14′), 14.0 (C-13/13′), 11.0 (C-15/15′).

### 4.4. Antibacterial Activity

#### 4.4.1. Culture Media and Chemicals

Para-iodonitrotetrazolium chloride (≥97% purity, INT) was used as the bacterial growth indicator and the efflux pump inhibitor was phenylalanine arginine *β*-naphthylamide (PA*β*N). Dimethyl sulfoxide (DMSO) served to dissolve extracts and products. Four antibiotics from four families, namely chloramphenicol, doxycycline (DOX), amikacin (AMK), and levofloxacin (LEV) were used. Chloramphenicol and amikacin were selected as representative efflux-pump substrates commonly used in mechanistic studies of Gram-negative MDR bacteria. Although chloramphenicol is less frequently used clinically, it remains a standard probe for efflux-pump activity. Amikacin, despite its parenteral administration, is a key aminoglycoside for MDR Gram-negative infections and a validated efflux substrate. These antibiotics were therefore chosen for their mechanistic relevance rather than their current prescribing frequency. Mueller–Hinton agar was used for the activation of bacteria; Mueller–Hinton broth was used for microdilution as a nutrient medium for bacteria. Eosin-Methylene Blue (EMB), MacConkey, and cetrimide agars were used to ensure the purity of strains and isolates of *Escherichia coli*, *Klebsiella pneumonia*, and *Pseudomonas aeruginosa*, respectively. The chemicals were obtained from Sigma-Aldrich (St. Quentin Fallavier, France).

#### 4.4.2. Microorganisms

The Gram-negative bacteria tested involved both reference strains and clinical isolates of *Escherichia coli* (ATCC 10536, AG100), *Klebsiella pneumoniae* (ATCC 11296, KP55), *Pseudomonas aeruginosa* (PA01, PA124), *Enterobacter aerogenes* (EA3, EA27), and *Providencia stuartii* (PS2636, NEA16). Their phenotypic and genotypic characteristics have been previously described [52].

#### 4.4.3. Minimal Inhibitory and Bactericidal Concentrations

The bacterial inoculum was prepared following the method described by Mbaveng et al. (2015) [53] and adjusted to the turbidity of a standard 0.5 McFarland solution (1.5 × 10^8^ CFU/mL). Test samples and the reference drug chloramphenicol were dissolved in 100 µL DMSO and brought to the desired volume with Mueller–Hinton broth (MHB). Plant extracts and fractions were prepared at 8192 µg/mL, sub-fractions and purified compounds at 1024 µg/mL, and antibiotics at 512 µg/mL. Minimal inhibitory concentrations (MICs) and minimal bactericidal concentrations (MBCs) were determined using a 96-well broth microdilution method combined with the rapid INT colorimetric assay. Chloramphenicol was used as a positive antibacterial control, while 2.5% DMSO in MHB and MHB alone were used as negative controls. The MIC was defined as the lowest concentration of a sample that completely inhibited bacterial growth after 18–24 h of incubation at 37 °C. The MBC was defined as the lowest concentration that did not induce a colour change upon the addition of INT following an additional 48 h of incubation. All experiments were performed in triplicate and repeated three times.

#### 4.4.4. Effect of Efflux Pumps on the Antibacterial Activity of the Samples

Test samples and chloramphenicol were also evaluated in the presence of the efflux pump inhibitor (EPI) PA*β*N at 30 μg/mL, following the method described by Kuete et al. (2011) [54]. The ratio of MIC (sample alone) to MIC (sample + PA*β*N), referred to as the activity improvement factor (AIF), was used to quantify the fold enhancement of antibacterial activity in the presence of PA*β*N.

#### 4.4.5. Antibiotic Potentiating Effect

The effect of combining the test samples with antibiotics was evaluated against five MDR bacterial strains. Initially, extracts were tested at sub-inhibitory concentrations (MIC/2, MIC/4, MIC/8, and MIC/16) in a preliminary assay on *Escherichia coli* AG100. This allowed the selection of appropriate sub-inhibitory concentrations (MIC/2 and MIC/4) for subsequent combination testing. The antibiotic-resistance modulating factor (AMF) was calculated as the ratio of the MIC of the antibiotic alone to the MIC in combination with the plant extract. Potentiation was considered significant when AMF ≥ 2 [55].

## 5. Conclusions

This study aimed to characterize compounds from the ethanol extract of *T. clypeatus* and evaluate their antibacterial potential against multidrug-resistant (MDR) bacteria. Chemical investigation of the ethyl acetate soluble fraction of the ethanol extract led to the isolation and identification of 19 compounds. Sub-fraction A, as well as compounds **3**, **4**, and **8**, exhibited moderate antibacterial activity against the tested strains. These compounds are substrates for bacterial efflux pumps, and their use enhanced the activity of the reference antibiotics ciprofloxacin, doxycycline, and amikacin. Overall, *T. clypeatus* and its bioactive constituents represent a promising source of antibacterial molecules, effective in combination with antibiotics against MDR Gram-negative bacteria. Although the present study focused on Gram-negative MDR bacteria due to their efflux-pump-mediated resistance profiles, future investigations will include clinically relevant Gram-positive pathogens such as methicillin-resistant Staphylococcus aureus (MRSA) and *Streptococcus pneumoniae* to broaden the antimicrobial spectrum assessment of the isolated compounds.

## Figures and Tables

**Table 1:** Antibacterial activities of extract, fractions and sub-fraction A from *Termitomyces clypeatus*.

Bacteria and Strains		EtOH Extract			EtOAc Fraction			*n*-BuOH Fraction			Sub-Fraction A		
		MIC	MBC	R	MIC	MBC	R	MIC	MBC	R	MIC	MBC	R
*Pseudomonas* *aeruginosa*	PA01	>2048	-	-	1024	>2048	-	2048	>2048	-	128	>256	-
	PA124	>2048	-	-	512	>2048	-	>2048	-	-	128	>256	-
*Klebsiella* *pneumoniae*	KP55	>2048	-	-	>2048	-	-	>2048	-	-	64	>256	-
	ATCC11296	>2048	-	-	>2048	-	-	>2048	-	-	256	256	1
*Escherichia coli*	AG100	2048	2048	1	1024	>2048	-	2048	>2048	-	>256	-	-
	ATCC10536	>2048	-	-	>2048	-	-	>2048	-	-	>256	-	-
*Providencia stuartii*	PS2636	2048	>2048	-	1024	>2048	-	2048	>2048	-	128	>256	-
	NEA16	>2048	-	-	>2048	-	-	>2048	-	-	>256	-	-
*Enterobacter aerogenes*	EA3	>2048	-	-	>2048	-	-	>2048	-	-	>256	-	-
	EA27	2048	>2048	-	2048	2048	-	>2048	-	-	>256	-	-

**R:** MBC/MIC ratio; **-:** not determined; **MIC:** minimum inhibitory concentration; **MBC:** minimum bactericidal concentration; MICs and MBCs are given in µg/mL.

**Table 2:** Antibacterial activities of compounds **3**–**6**.

Bacteria and Strains		3			4			5			6		
		MIC	MBC	R	MIC	MBC	R	MIC	MBC	R	MIC	MBC	R
*Pseudomonas* *aeruginosa*	PA01	256	256	1	128	256	2	>256	-	-	>256	-	-
	PA124	128	256	2	128	-	-	>256	-	-	>256	-	-
*Klebsiella* *pneumoniae*	KP55	>256	-		>256	-	-	>256	-	-	>256	-	-
	ATCC11296	256	>256	-	256	>256	-	128	256	2	>256	-	-
*Escherichia coli*	AG100	128	>256	-	256	256	1	256	>256	-	>256	-	-
	ATCC10536	64	64	1	64	>256	-	32	128	4	128	256	2
*Providencia stuartii*	PS2636	128	-	-	128	128	1	>256	-	-	256	256	1
	NEA16	256	>256	-	256	>256	-	>256	-	-	>256	-	-
*Enterobacter aerogenes*	EA3	256	>256	-	128	>256	-	256	256	1	>256	-	-
	EA27	>256	-	-	256	>256	-	>256	-	-	>256	-	-

**R:** MBC/MIC ratio; **-:** not determined; **MIC:** minimum inhibitory concentration; **MBC:** minimum bactericidal concentration; MICs and MBCs are given in µg/mL.

**Table 3:** Antibacterial activities of compounds **7**, **8**, and **9**.

Bacteria and Strains		7			8			9			Chloramphenicol		
		MIC	MBC	R	MIC	MBC	R	MIC	MBC	R	MIC	MBC	R
*Pseudomonas* *aeruginosa*	PA01	>256	-	-	>256	>256	-	>256	-	-	32	64	2
	PA124	>256	-	-	256	>256	-	256	>256	-	8	128	32
*Klebsiella* *pneumoniae*	KP55	>256	-	-	>256	>256	-	>256	-	-	64	128	2
	ATCC11296	>256	-	-	256	256	1	>256	-	-	16	32	2
*Escherichia coli*	AG100	256	256	1	256	256	1	256	>256	-	2	16	8
	ATCC10536	256	>256	-	>256	>256	-	256	256	1	32	32	1
*Providencia stuartii*	PS2636	>256	-	-	128	256	2	>256	-	-	16	>128	-
	NEA16	>256	-	-	>256	>256	-	>256	-	-	64	>128	-
*Enterobacter aerogenes*	EA3	>256	-	-	64	128	2	>256	-	-	32	128	4
	EA27	>256	-	-	>256	>256	-	>256	-	-	32	>128	-

**R:** MBC/MIC ratio; **-:** not determined; **MIC:** minimum inhibitory concentration; **MBC:** minimum bactericidal concentration.

**Table 4:** Minimal inhibitory concentrations of samples from *T. clypeatus* in absence and presence of Pa*β*N.

Bacteria and Strains	Sub-Fraction A			3			4			8			Chloramphenicol		
	MIC Alone	MIC + PA*β*N	R	MIC Alone	MIC + PA*β*N	R	MIC Alone	MIC + PA*β*N	R	MIC Alone	MIC + PA*β*N	R	MIC Alone	MIC + PA*β*N	R
PA01	1024	>2048	/	256	/	/	128	32	4	>256	/	/	32	32	1
PA124	512	>2048	/	128	512	4	128	64	2	256	128	2	64	64	1
KP55	>2048	/	/	>256	/	/	>256	32	>8	>256	256	1	128	16	8
ATCC11296	256	>2048	/	256	32	8	256	64	4	256	256	1	64	16	4
AG100	1024	>2048	/	128	32	4	256	256	1	256	32	8	128	64	2
ATCC10536	>2048	16	>128	64	4	16	64	32	2	>256	8	>32	256	256	1
PS2636	1024	256	4	128	4	32	128	128	1	128	4	32	128	8	16
NEA16	>2048	/	/	256	/	/	256	128	2	>256	/	/	128	64	2
EA3	>2048	/	/	256	4	>64	128	32	4	64	4	16	64	4	16
EA27	>2048	/	/	>256	128	>2	256	32	8	>256	/	/	32	16	2

**PA01:** *Pseudomonas aeruginosa* PA01; **PA124:** *Pseudomonas aeruginosa* PA124; **KP55:** *Klebsiella pneumoniae* KP55; **ATCC11296:** *Klebsiella pneumoniae* ATCC11296; **AG100:** *Escherichia coli* AG100; **ATCC10536*:*** *Escherichia coli* ATCC10536; **PS2636:** *Providencia stuartii* PS2636; **NEA16:** *Providencia stuartii* NEA16; **EA3:** *Enterobacter aerogenes* EA3; **EA27:** *Enterobacter aerogenes* EA27; **MIC:** minimum inhibitory concentration; **MIC with PA*β*N:** minimum inhibitory concentration in the presence of PA*β*N; **R:** MIC alone/MIC with PA*β*N ratio.

**Table 5:** Antibiotic potentiating effect of sub-fractions.

ATBs	Sub-Fractions	Concentrations	MIC of Antibiotics in the Presence of Sub-Fractions (AMF)					PSP (%)
			KP55	AG100	PS2636	PA124	EA3	
CIP		0	>512	>512	256	512	>512	
	A	MIC/2	512 (-)	>512 (-)	128 (2)	512 (1)	256 (~2)	40
		MIC/4	>512 (-)	>512 (-)	128 (2)	512 (1)	512 (~1)	20
	B	MIC/2	˂4 (128)	˂4 (128)	˂4 (256)	˂4 (128)	˂4 (128)	100
		MIC/4	˂4 (128)	˂4 (128)	˂4 (256)	˂4 (128)	˂4 (128)	100
	C	MIC/2	˂4 (128)	˂4 (128)	˂4 (256)	˂4 (128)	˂4 (128)	100
		MIC/4	˂4 (128)	˂4 (128)	˂4 (256)	˂4 (128)	˂4 (128)	100
	D	MIC/2	˂4 (128)	˂4 (128)	˂4 (256)	˂4 (128)	˂4 (128)	100
		MIC/4	˂4 (128)	˂4 (128)	˂4 (256)	˂4 (128)	˂4 (128)	100
DOX		0	16	˂4	32	64	64	
	A	MIC/2	˂4 (4)	˂4 (1)	32 (1)	128 (0.5)	8 (8)	40
		MIC/4	8 (2)	˂4 (1)	˂4 (8)	64 (1)	8 (8)	60
	B	MIC/2	˂4 (4)	˂4 (1)	˂4 (8)	˂4 (16)	˂4 (16)	80
		MIC/4	˂4 (4)	˂4 (1)	˂4 (8)	˂4 (16)	˂4 (16)	80
	C	MIC/2	˂4 (4)	˂4 (1)	˂4 (8)	˂4 (16)	˂4 (16)	0
		MIC/4	˂4 (4)	˂4 (1)	˂4 (8)	˂4 (16)	˂4 (16)	20
	D	MIC/2	˂4 (4)	˂4 (1)	˂4 (8)	˂4 (16)	˂4 (16)	40
		MIC/4	˂4 (4)	˂4 (1)	˂4 (8)	˂4 (16)	˂4 (16)	20
AMK		0	˂4	16	˂4	32	˂4	
	A	MIC/2	˂4 (1)	16 (1)	˂4 (1)	8 (4)	˂4 (1)	20
		MIC/4	˂4 (1)	16 (1)	˂4 (1)	64 (0.5)	˂4 (1)	0
	B	MIC/2	8 (0.5)	˂4 (4)	32 (0.125)	˂4 (8)	˂4 (1)	40
		MIC/4	16 (0.25)	˂4 (4)	32 (0.125)	˂4 (8)	8 (0.5)	40
	C	MIC/2	˂4 (1)	32 (0.5)	˂4 (1)	˂4 (8)	16 (0.25)	20
		MIC/4	˂4 (1)	32 (0.5)	32 (0.125)	˂4 (8)	16 (0.25)	20
	D	MIC/2	8 (0.5)	16 (1)	16 (0.25)	˂4 (8)	16 (0.25)	20
		MIC/4	16 (0.25)	64 (0.25)	16 (0.25)	˂4 (8)	16 (0.25)	20

**KP55:** *Klebsiella pneumoniae* KP55; **MIC:** minimum inhibitory concentration; **AMF:** activity modulating factors; **PSP (%):** percentage of strain where potentiation effect was observed; **ATBs:** antibiotics; **CIP:** ciprofloxacin; **DOX:** doxycycline; **AMK:** amikacin.

**Table 6:** Antibiotic potentiating effect of compounds **3**, **4**, and **8**.

ATBs	Compounds	Concentration	MIC of Antibiotics in the Presence of Extract (AMF)					PSP (%)
			KP55	AG100	PS2636	PA124	EA3	
CIP		0	>512	>512	256	512	>512	
	**3**	MIC/2	>512 (-)	>512 (-)	512 (0.5)	512 (1)	256 (~2)	20
		MIC/4	>512 (-)	>512 (-)	512 (−2)	512 (1)	256 (~2)	20
	**4**	MIC/2	256 (2)	>512 (-)	128 (2)	512 (1)	512 (~1)	40
		MIC/4	>512 (-)	>512 (-)	128 (2)	512 (1)	512 (~1)	20
	**8**	MIC/2	>512 (-)	>512 (-)	256 (1)	512 (1)	256 (~2)	20
		MIC/4	>512 (-)	>512 (-)	256 (1)	512 (1)	512 (~1)	0
DOX		0	16	˂4	32	64	64	
	**3**	MIC/2	64 (0.25)	32 (0.125)	64 (0.5)	64 (1)	64 (1)	0
		MIC/4	32 (0.5)	64 (0.06)	64 (0.5)	64 (1)	8 (8)	20
	**4**	MIC/2	˂4 (4)	˂4 (1)	32 (1)	64 (1)	64 (1)	20
		MIC/4	˂4 (4)	8 (0.5)	32 (1)	64 (1)	16 (4)	40
	**8**	MIC/2	32 (0.5)	16 (0.25)	16 (2)	64 (1)	˂4 (16)	40
		MIC/4	32 (−2)	32 (−8)	16 (2)	64 (1)	64 (1)	20
AMK		0	˂4	16	˂4	32	˂4	
	**3**	MIC/2	8 (0.5)	32 (0.5)	˂4 (1)	64 (0.5)	˂4 (1)	0
		MIC/4	8 (0.5)	32 (0.5)	˂4 (1)	64 (0.5)	˂4 (1)	0
	**4**	MIC/2	˂4 (1)	32 (0.5)	˂4 (1)	16 (2)	˂4 (1)	20
		MIC/4	˂4 (1)	32 (0.5)	˂4 (1)	16 (2)	˂4 (1)	20
	**8**	MIC/2	16 (0.25)	32 (0.5)	˂4 (1)	64 (0.5)	˂4 (1)	0
		MIC/4	8 (0.5)	32 (0.5)	˂4 (1)	32 (1)	˂4 (1)	0

**MIC:** minimum inhibitory concentration; **AMF:** activity modulating factors; **PSP (%):** percentage of strain where potentiation effect was observed; **ATBs:** antibiotics; **CIP:** ciprofloxacin; **DOX:** doxycycline; **AMK:** amikacin.

## Data Availability

The original contributions presented in this study are included in the article/Supplementary Material. Further inquiries can be directed to the corresponding authors.

## Supplementary Materials

The following supporting information can be downloaded at: https://www.mdpi.com/article/10.3390/ph19050737/s1, Mass spectrometric and NMR spectra of compounds **1** and **2** are available as Supplementary Material.
