# Supplementary material for: Antibacterial Activity of Extract, Fractions, and Compounds from Termitomyces clypeatus R. Heim (Lyophyllaceae) Against Multidrug-Resistant Bacteria Overexpressing Efflux Pumps
Source: Pharmaceuticals (Basel). 2026 May 7;19(5):737. doi: 10.3390/ph19050737 (PMC13209873; doi:10.3390/ph19050737)
Supplement: Supplementary file 1 [file pharmaceuticals-19-00737-s001.zip › pharmaceuticals-4239623-supplementary.pdf]

pharmaceuticals

MDPI

Article

Antibacterial activity of extract, fractions, and compounds from *Termitomyces clypeatus* R. Heim (Lyophyllaceae) against multi-drug-resistant bacteria overexpressing efflux pumps

Jenifer R. N. Kuete[1], Jason B. T. Kuete[2], Joris Baier[3], Niklas Ehlenz [3], Simionne L. K. Tonga[1], Bienvenu Tsakem[1,4], Refilwe Matshitse[4], Borice T. Tsafack[1], Paul Eckhardt[3], Beaudelaire K. Ponou[1], Till Opatz[3], Léon Azefack Tapondjou[1], Ilhami Celik[5], Xavier Siwe-Noundou[4]*, Rémy B. Teponno[1]*

1 Department of Chemistry, Faculty of Science, University of Dschang, Dschang P.O. Box 67, Cameroon; jeniferkuete@gmail.com (J.R.N.K.); simionnekuitcha@gmail.com (S.L.K.T.); btsakem23@gmail.com (B.T.); boricet@yahoo.fr (B.T.T.); beaudelaireponou@yahoo.fr (B.K.P.); tapondjou2001@yahoo.fr (L.A.T.)
2 Department of Biochemistry, Faculty of Science, University of Dschang, Dschang P.O. Box 67, Cameroon; kuetejason7@gmail.com
3 Department of Chemistry, Johannes Gutenberg University of Mainz, Duesbergweg 10-14, D-55128 Mainz, Germany; jbaier01@uni-mainz.de (J.B.); ehlenz@uni-mainz.de (N.E.); eckhardt@uni-mainz.de (P.E.); opatz@uni-mainz.de (T.O.)
4 Department of Pharmaceutical Sciences, School of Pharmacy, Sefako Makgatho Health Sciences University, Pretoria 0204, South Africa, South Africa; refilwemandla@yahoo.com
5 Department of Chemistry, Faculty of Science, Eskisehir Technical University, Eskisehir 26470, Turkey; ilcelik@eskisehir.edu.tr
* Correspondence: xavier.siwenoundou@smu.ac.za (X.S.-N.); remyteponno@gmail.com (R.B.T.)

Abstract

Background/Objectives: Microbial resistance to antibiotics has become a major global public health problem, threatening the effectiveness of current therapeutic strategies. The present study seeks to investigate natural compounds originating from fungal sources for their ability to interfere with efflux pump-mediated resistance in multidrug-resistant (MDR) bacteria, with the overarching goal of uncovering new candidates for antimicrobial therapeutic development. A chemical investigation of the ethanol extract of *Termitomyces clypeatus* was carried out to isolate and identify its constituents. **Methods:** Structural elu-cidation of the isolated metabolites was achieved through 1D and 2D NMR spectroscopy supported by mass spectrometric data. The crude extract and the purified compounds were then evaluated for their antibacterial activities individually, in the presence of an efflux pump inhibitor, and in combination with three antibiotics, using standardized mi-crodilution assays. **Results:** Chromatographic separation of the extract yielded eleven known compounds, including three sphingolipids: (9*Z*,12*Z*)-N-(1,3,4-trihydroxyoctadecan-2-yl)octadeca-9,12-dienamide (**1**), 2-hydroxy-*N*-(1,3,4-trihydroxyoctadecan-2-yl)hexadecanamide (**2**), and cerebroside B (**3**); four steroids: ergosterol (**4**), cerevisterol (**5**), ergosterol peroxide (**6**), and 5α,6α-epoxy-(22*E*,24*R*)-ergosta-8(14),22-diene-3β,7α-diol (**7**); one alkaloid: piperine (**8**); one carbohydrate: D-mannitol (**9**); and two phthalates: dimethyl phthalate (**10**) and bis(2-ethylhexyl) terephthalate (**11**). GC–MS analysis led to the identification of eight fatty acid derivatives (**12–19**). Sub-fraction A, along with compounds **3**, **4**, and **8**, exhibited moderate antibacterial activity against some tested strains, with MIC values of 64 µg/mL. These compounds were identified as substrates of bacterial efflux pumps, and their presence enhanced the antibacterial effects of ciprofloxacin, doxycycline, and amikacin. **Conclusions:** The findings of the present work indicate that *Termitomyces clypeatus* contains compounds with potential therapeutic value, as adjuvants that enhance the activity of conventional antibiotics.

Keywords: *Termitomyces clypeatus*, antibacterial activity, efflux pumps; antibiotics association

Figure S1. HRESIMS (-) of compound **1**
Figure S2. HRESIMS (+) of compound **1**
Figure S3. ^{1}H NMR spectrum (600 MHz, DMSO-d_6) of compound **1**
Figure S4. ^{13}C NMR spectrum (150 MHz, DMSO-d_6) of compound **1**
Figure S5. ^{1}H-^{1}H COSY spectrum of compound **1**
Figure S6. HSQC spectrum of compound **1**
Figure S7. HMBC spectrum of compound **1**
Figure S8. NOESY spectrum of compound **1**
FigureS9. LCMS (+) of compound **2**
Figure S10. ^{1}H NMR spectrum (600 MHz, Pyridine-d_5) of compound **2**
Figure S11. ^{13}C NMR spectrum (150 MHz, Pyridine-d_5) of compound **2**
Figure S12. ^{1}H-^{1}H COSY spectrum of compound **2**
Figure S13. HSQC spectrum of compound **2**
Figure S14. HMBC spectrum of compound **2**
Figure S15. NOESY spectrum of compound **2**
Figure S16. LCESIMS of compound **3**
Figure S17. ^{1}H NMR spectrum (600 MHz, CD_3OD) of compound **3**
Figure S18. ^{13}C NMR spectrum (150 MHz, CD_3OD) of compound **3**
Figure S19. ^{1}H-^{1}H COSY spectrum of compound **3**
Figure S20. HSQC spectrum of compound **3**
Figure S21. HMBC spectrum of compound **3**
Figure S22. NOESY spectrum of compound **3**
Figure S23. ^{1}H NMR spectrum (600 MHz, $CDCl_3$) of compound **4**
Figure S24. ^{13}C NMR spectrum (150 MHz, $CDCl_3$) of compound **4**
Figure S25. ^{1}H-^{1}H COSY spectrum of compound **4**
Figure S26. HSQC spectrum of compound **4**
Figure S27. HMBC spectrum of compound **4**
Figure S28. ^{1}H NMR spectrum of compound **5**
Figure S29. ^{13}C NMR spectrum (600 MHz, DMSO-d_6) of compound **5**
Figure S30. ^{1}H-^{1}H COSY spectrum (150 MHz, DMSO-d_6) of compound 5
Figure S31. HSQC spectrum of compound **5**
Figure S32. HMBC spectrum of compound **5**
Figure S33. ^{1}H NMR spectrum (600 MHz, $CDCl_3$) of compound **6**
Figure S34. ^{13}C NMR spectrum (150 MHz, $CDCl_3$) of compound **6**
Figure S35. ^{1}H-^{1}H COSY spectrum of compound **6**
Figure S36. HSQC spectrum of compound **6**
Figure S37. HMBC spectrum of compound **6**
Figure S38. NOESY spectrum of compound **6**
Figure S39. ^{1}H NMR spectrum (600 MHz, CD_3OD) of compound **7**
Figure S40. ^{13}C NMR spectrum (150 MHz, CD_3OD) of compound **7**
Figure S41. ^{1}H-^{1}H COSY spectrum of compound **7**
Figure S42. HSQC spectrum of compound **7**
Figure S43. HMBC spectrum of compound **7**
Figure S44. ^{1}H NMR spectrum (600 MHz, CD_3OD) of compound **8**
Figure S45. ^{13}C NMR spectrum (150 MHz, CD_3OD) of compound **8**
Figure S46. ^{1}H-^{1}H COSY spectrum of compound **8**

Figure S47. HSQC spectrum of compound **8**
Figure S48. HMBC spectrum of compound **8**
Figure S49. ^{1}H NMR spectrum (600 MHz, CD_3OD) of compound **9**
Figure S50. ^{13}C NMR spectrum (150 MHz, CD_3OD) of compound **9**
Figure S51. ^{1}H-^{1}H COSY spectrum of compound **9**
Figure S52. HSQC spectrum of compound **9**
Figure S53. HMBC spectrum of compound **9**
Figure S54. LCESIMS (+) of compound **10**
Figure S55. ^{1}H NMR spectrum (600 MHz, CD_3OD) of compound **10**
Figure S56. ^{13}C NMR spectrum (150 MHz, CD_3OD) of compound **10**
Figure S57. ^{1}H-^{1}H COSY spectrum of compound **10**
Figure S58. HSQC spectrum of compound **10**
Figure S59. HMBC spectrum of compound **10**
Figure S60. ^{1}H NMR spectrum (600 MHz, CD_3OD) of compound **11**
Figure S61. ^{13}C NMR spectrum (150 MHz, CD_3OD) of compound **11**
Figure S62. ^{1}H-^{1}H COSY spectrum of compound **11**
Figure S63. HSQC spectrum of compound **11**
Figure S64. HMBC spectrum of compound **11**
Figure S65. GC Chromatogram of Sub-fraction A of the EtOH extract of *T. clypeatus*
Figure S66. EI-MS of compound **12**
Figure S67. EI-MS of compound **13**
Figure S68. EI-MS of compound **14**
Figure S69. EI-MS of compound **15**
Figure S70. EI-MS of compound **16**
Figure S71. EI-MS of compound **17**
Figure S72. EI-MS of compound **18**
Figure S73. EI-MS of compound **19**

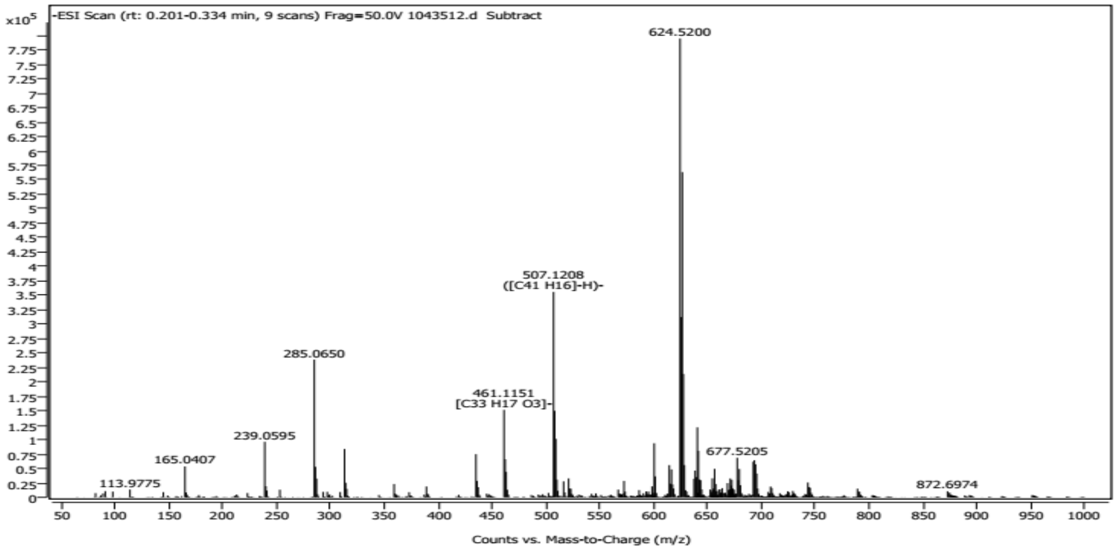

Figure S1. HRESIMS (-) of compound **1**

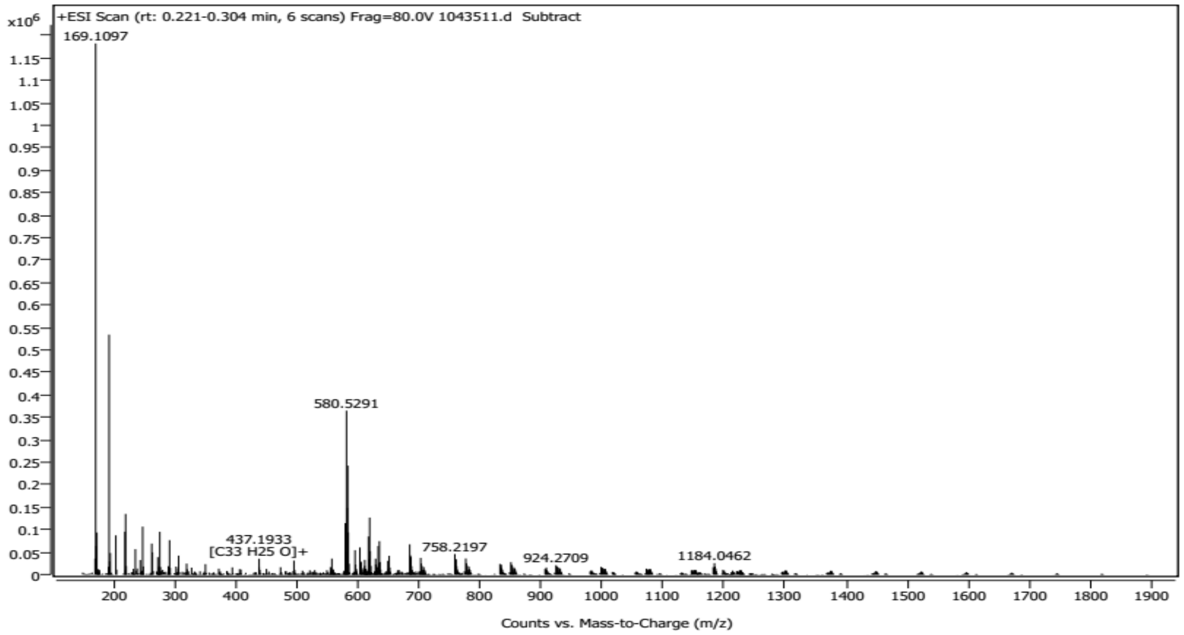

Figure S2. HRESIMS (+) of compound **1**

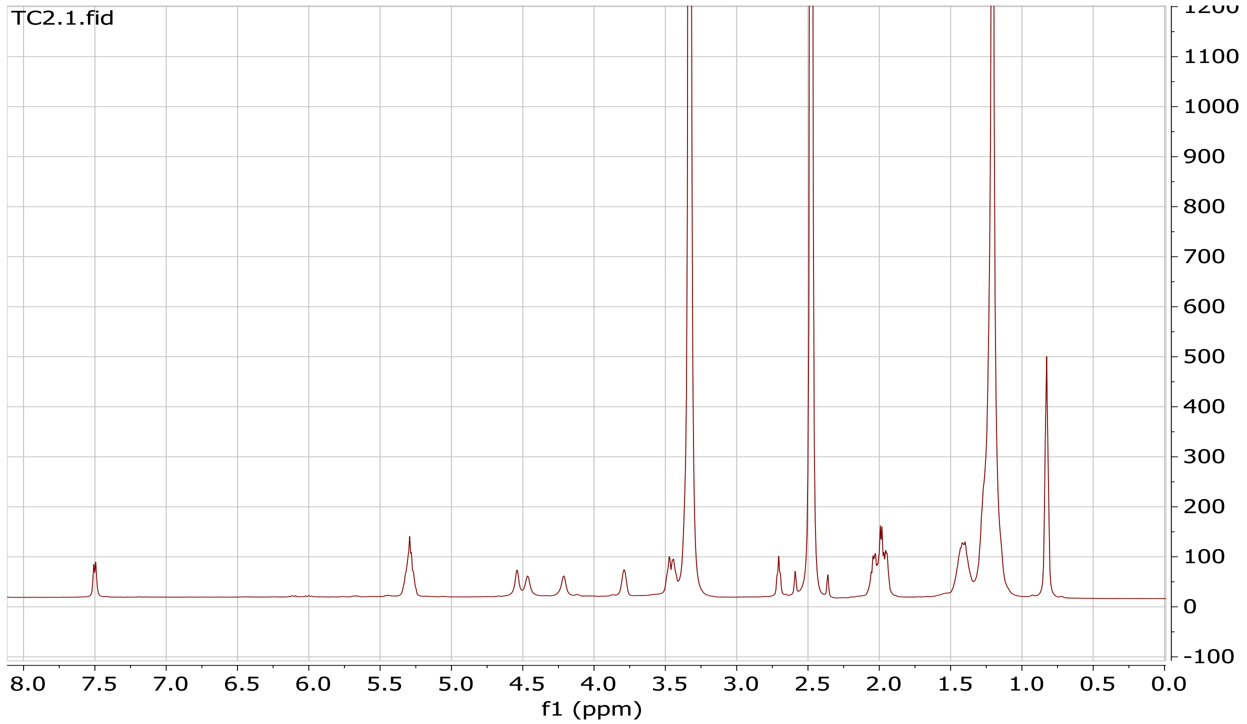

Figure S3. ^{1}H NMR spectrum (600 MHz, DMSO-d_6) of compound **1**

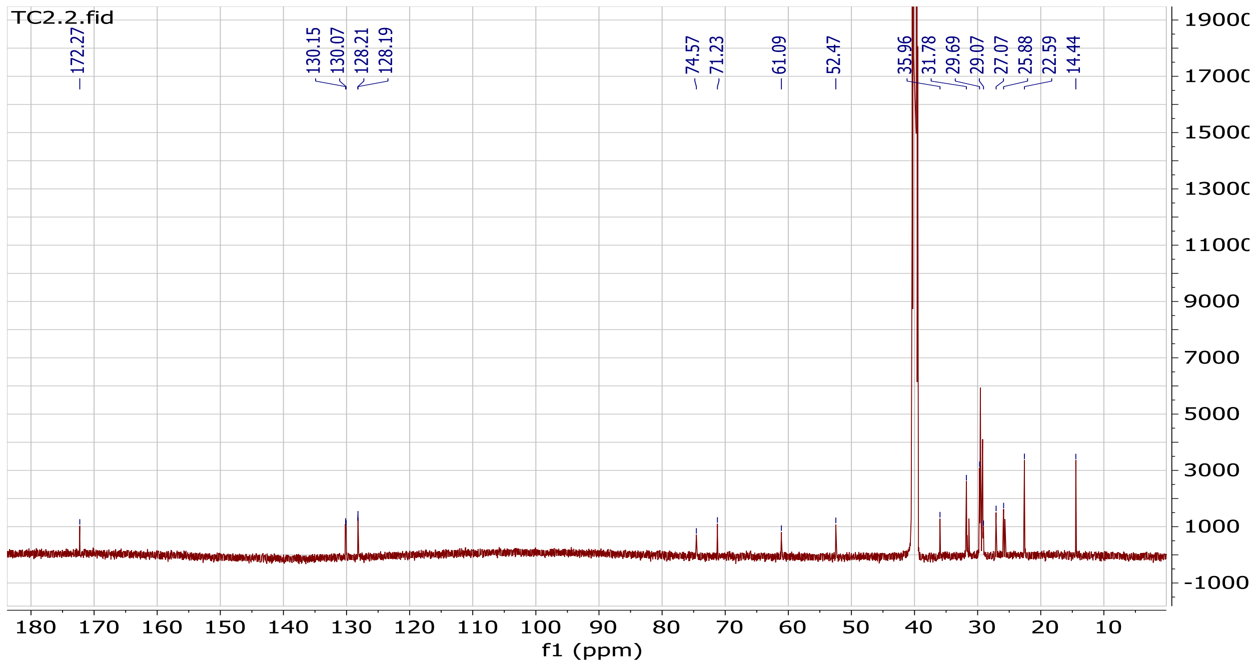

Figure S4. ^{13}C NMR spectrum (150 MHz, DMSO-d_6) of compound **1**

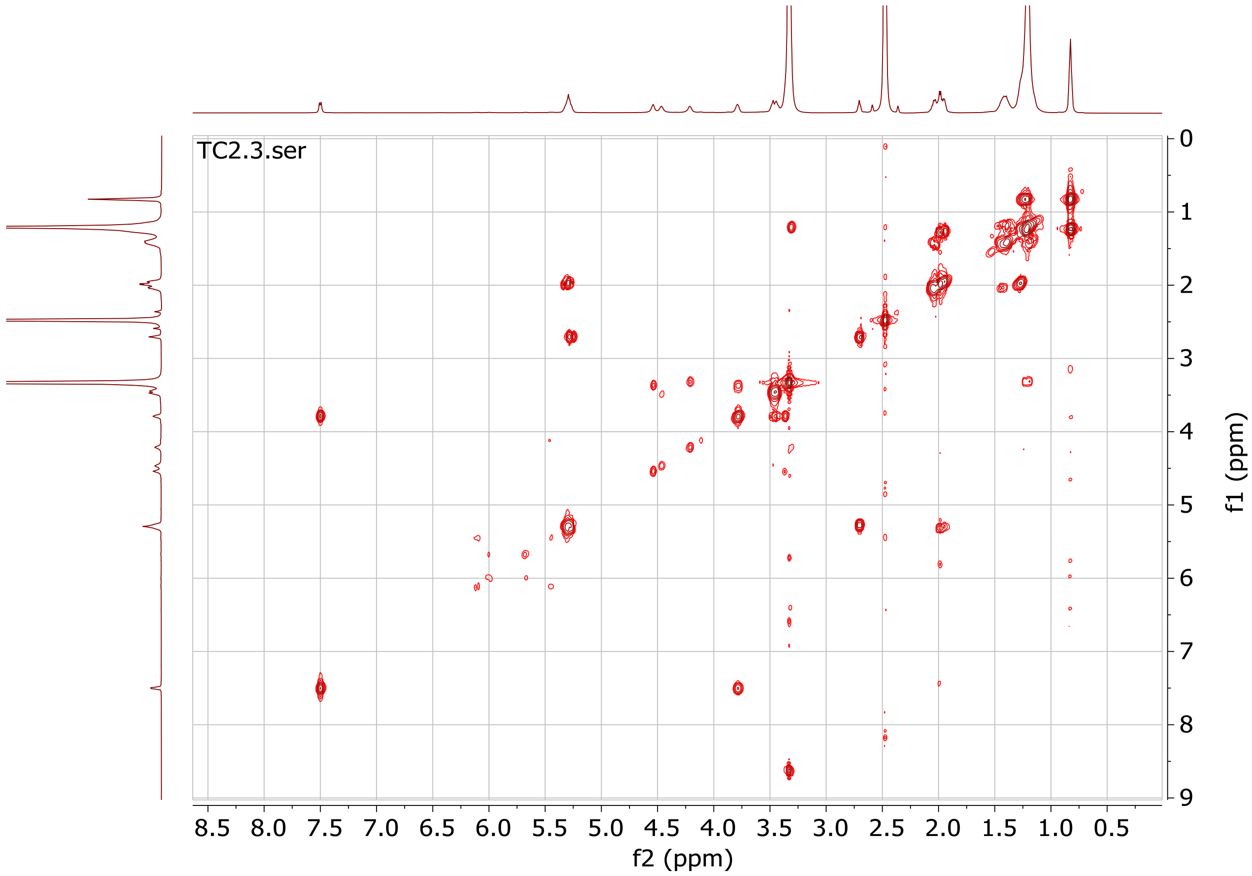

Figure S5. ^{1}H-^{1}H COSY spectrum of compound **1**

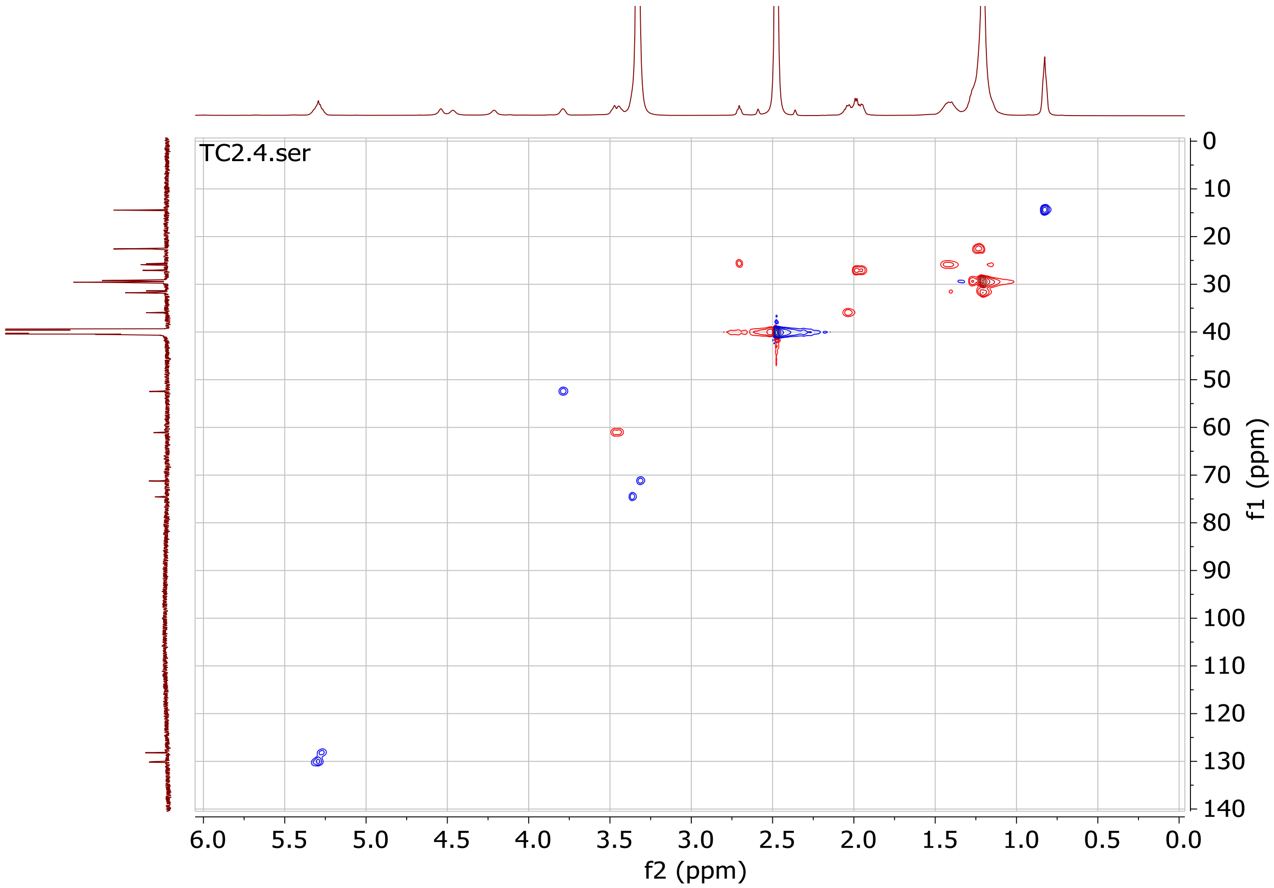

Figure S6. HSQC spectrum of compound **1**

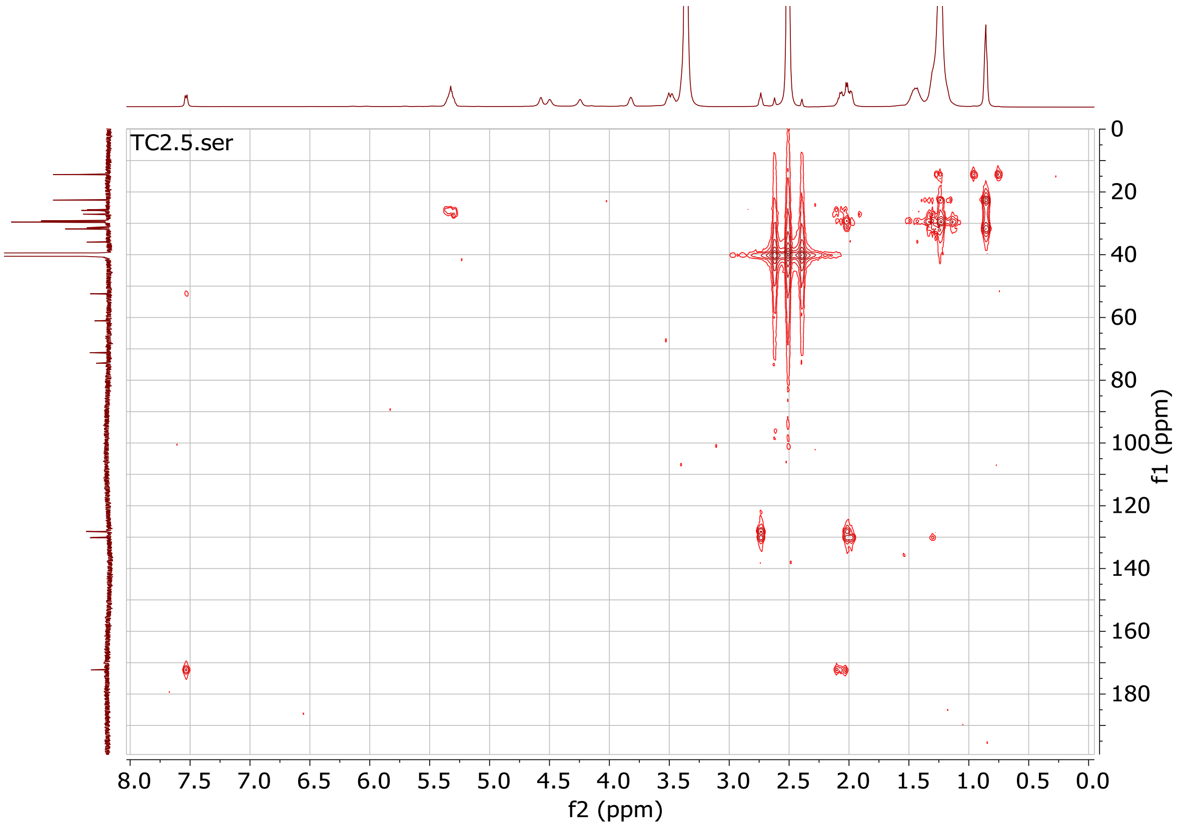

Figure S7. HMBC spectrum of compound **1**

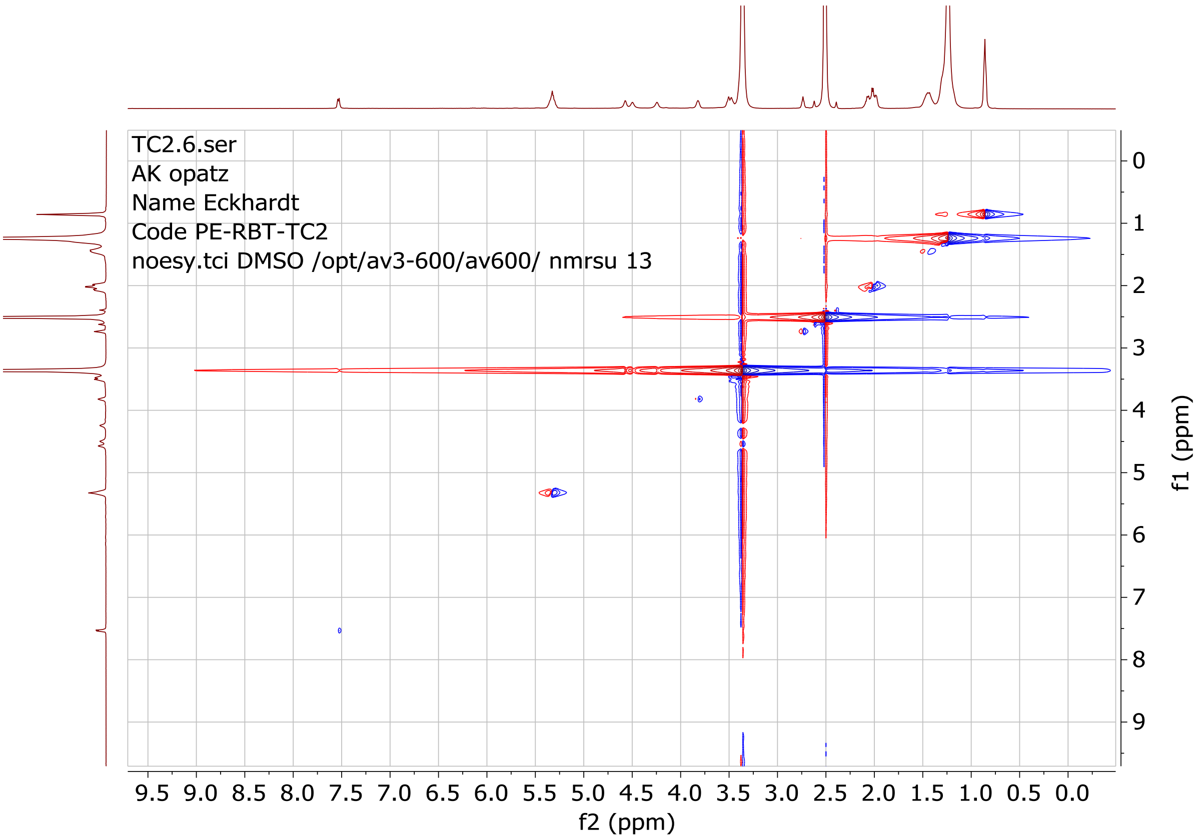

Figure S8. NOESY spectrum of compound **1**

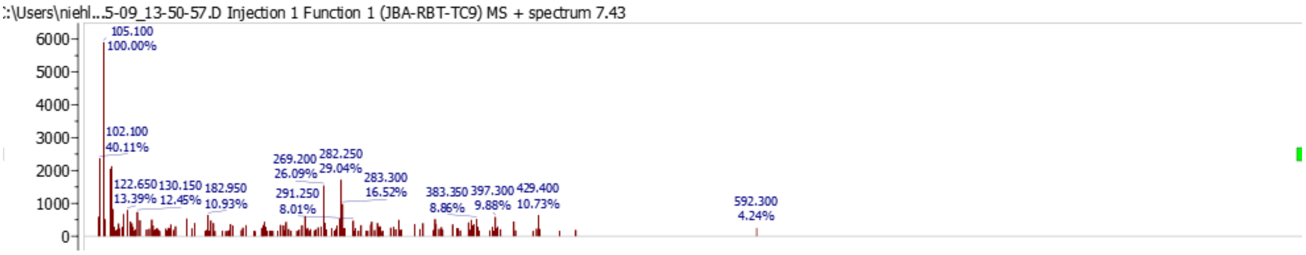

Figure S9. ESI-MS (+) of compound **2**

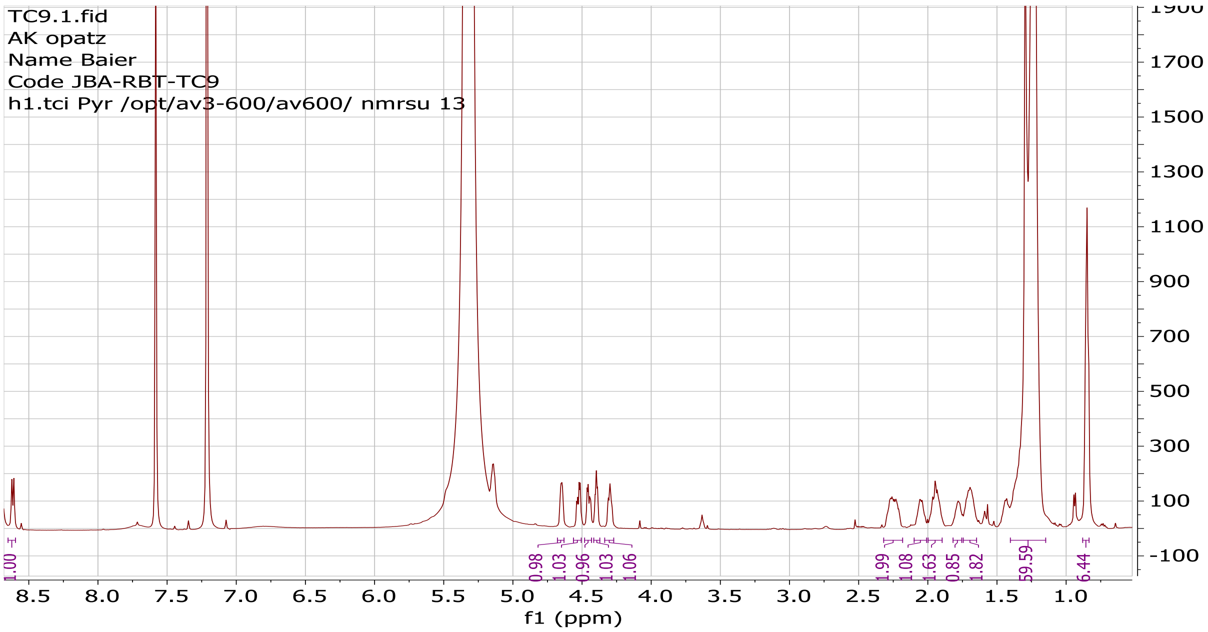

Figure S10. ^{1}H NMR spectrum (600 MHz, Pyridine-d_5) of compound **2**

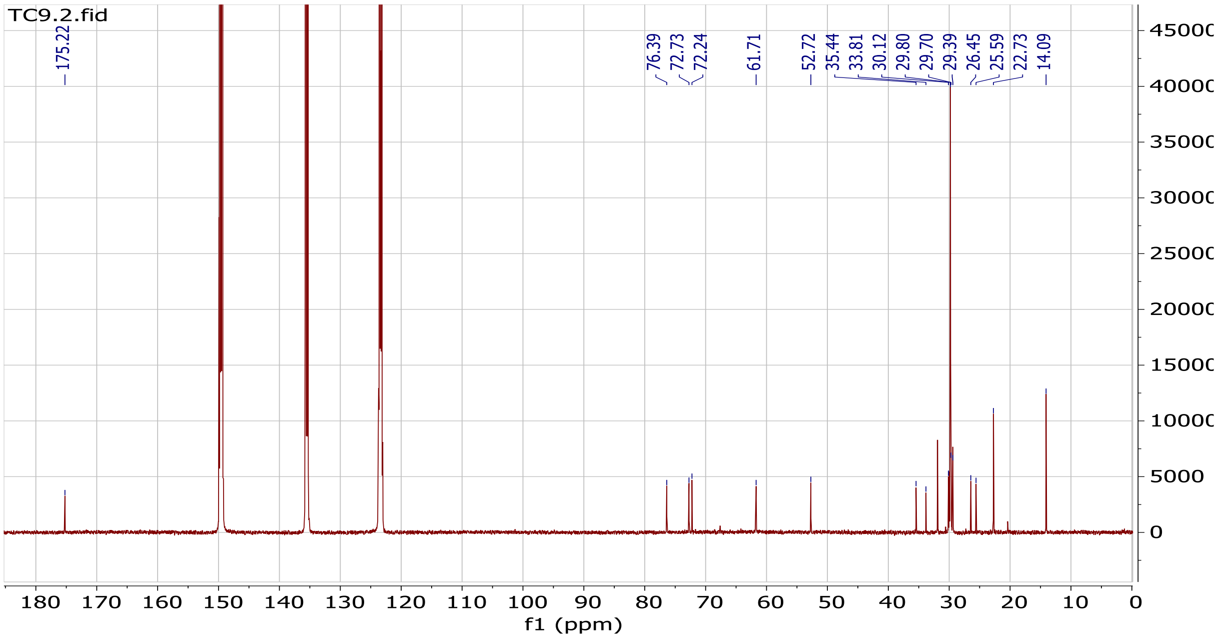

Figure S11. ^{13}C NMR spectrum (600 MHz, Pyridine-d_5) of compound **2**

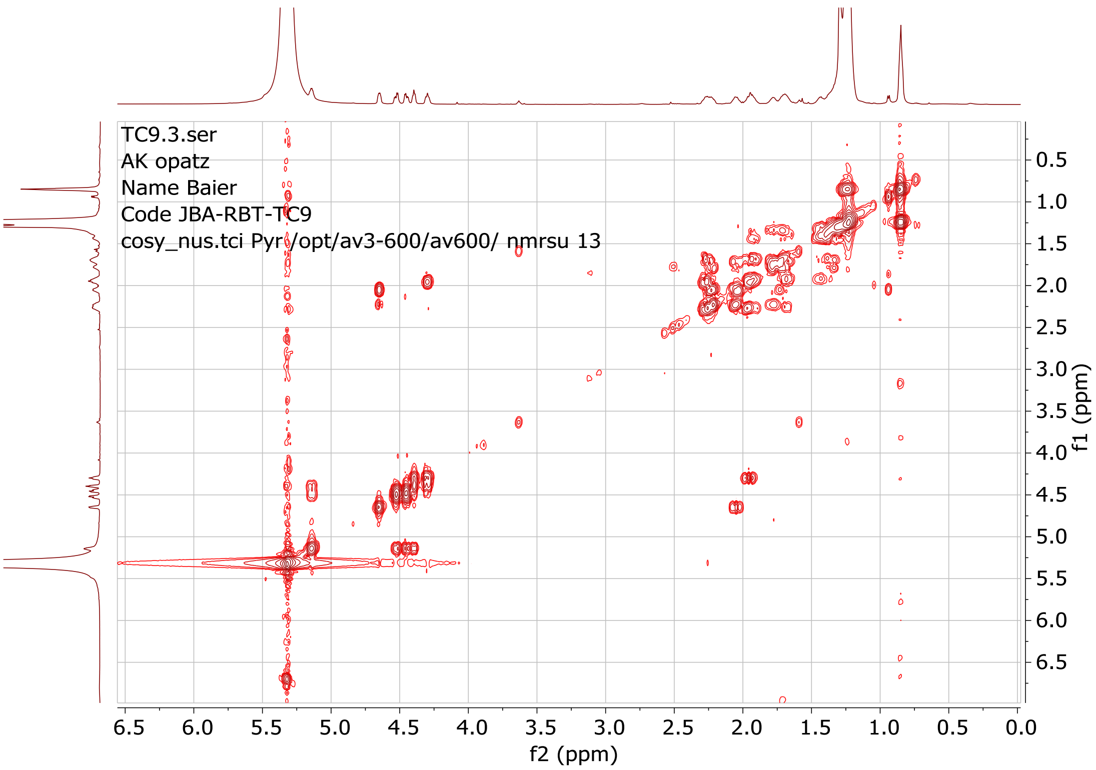

Figure S12. ^{1}H-^{1}H COSY spectrum of compound **2**

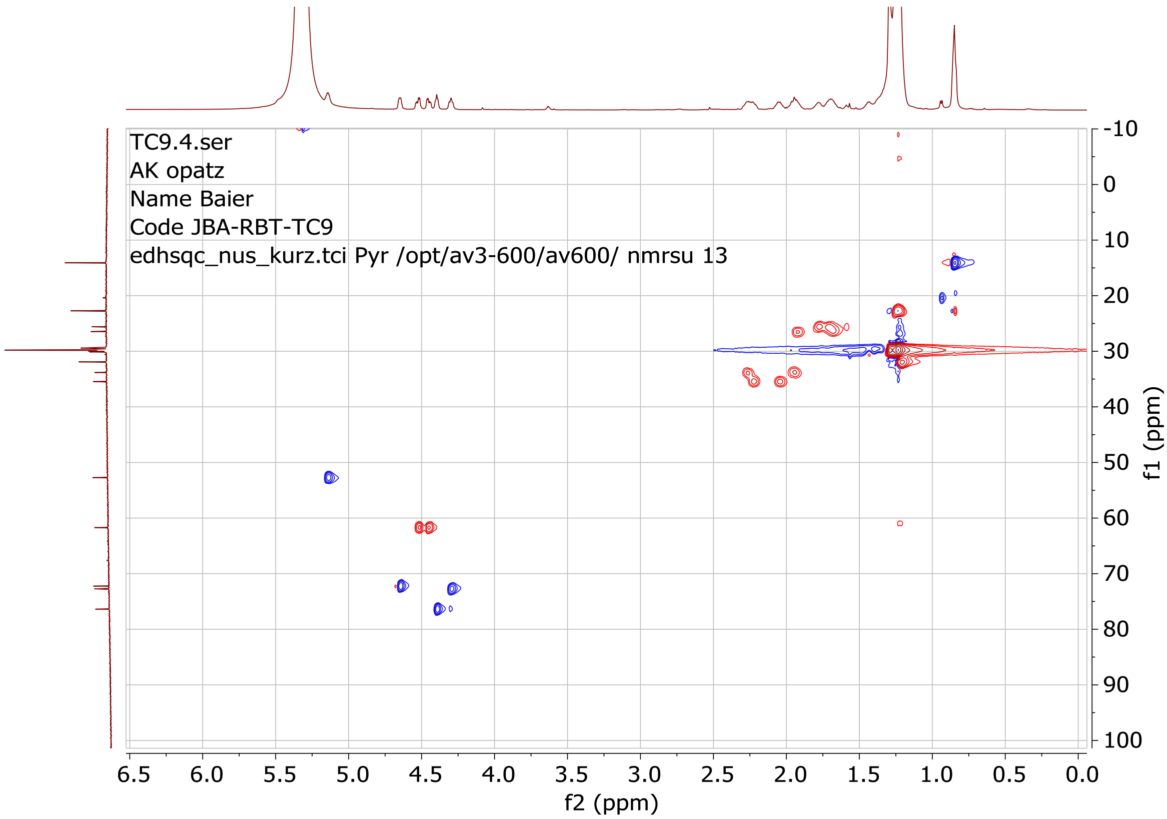

Figure S13. HSQC spectrum of compound **2**

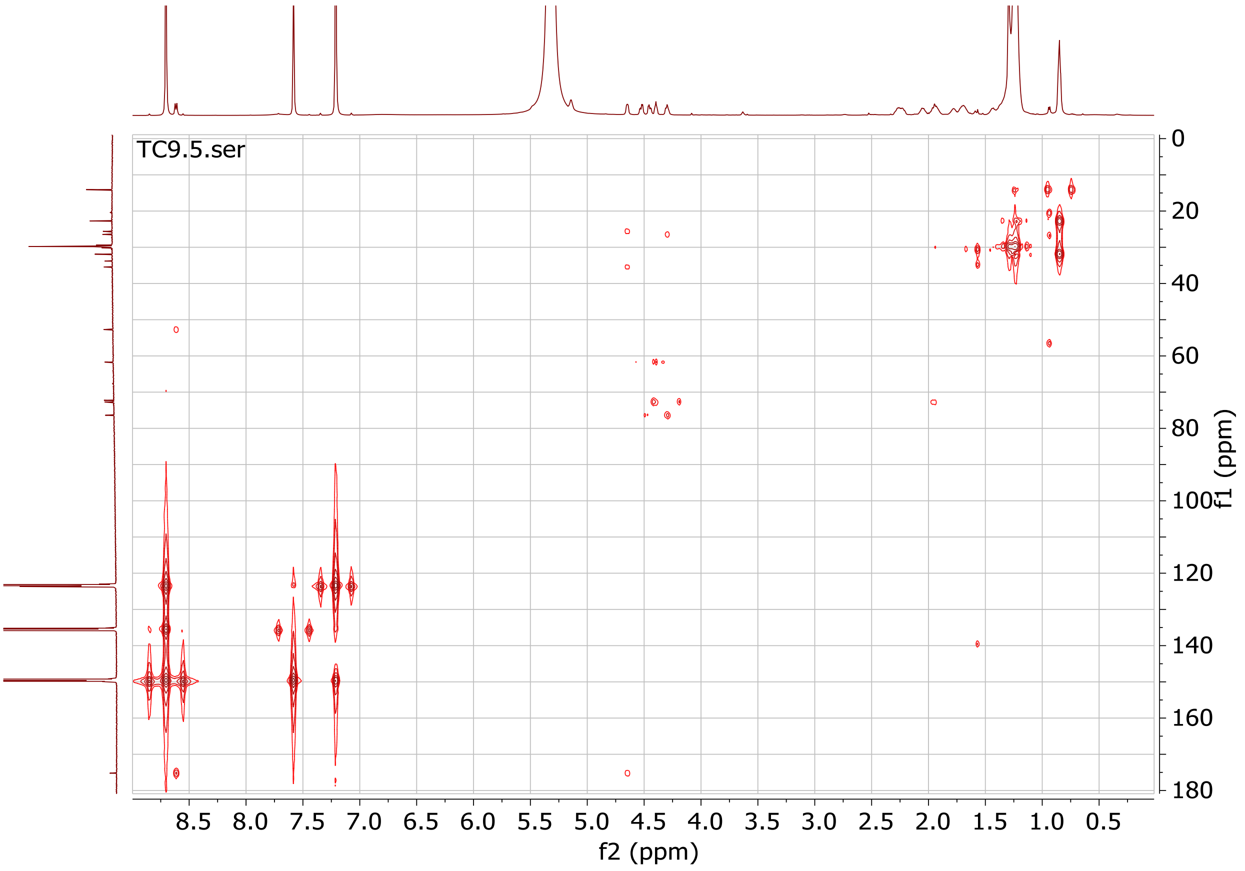

Figure S14. HMBC spectrum of compound **2**

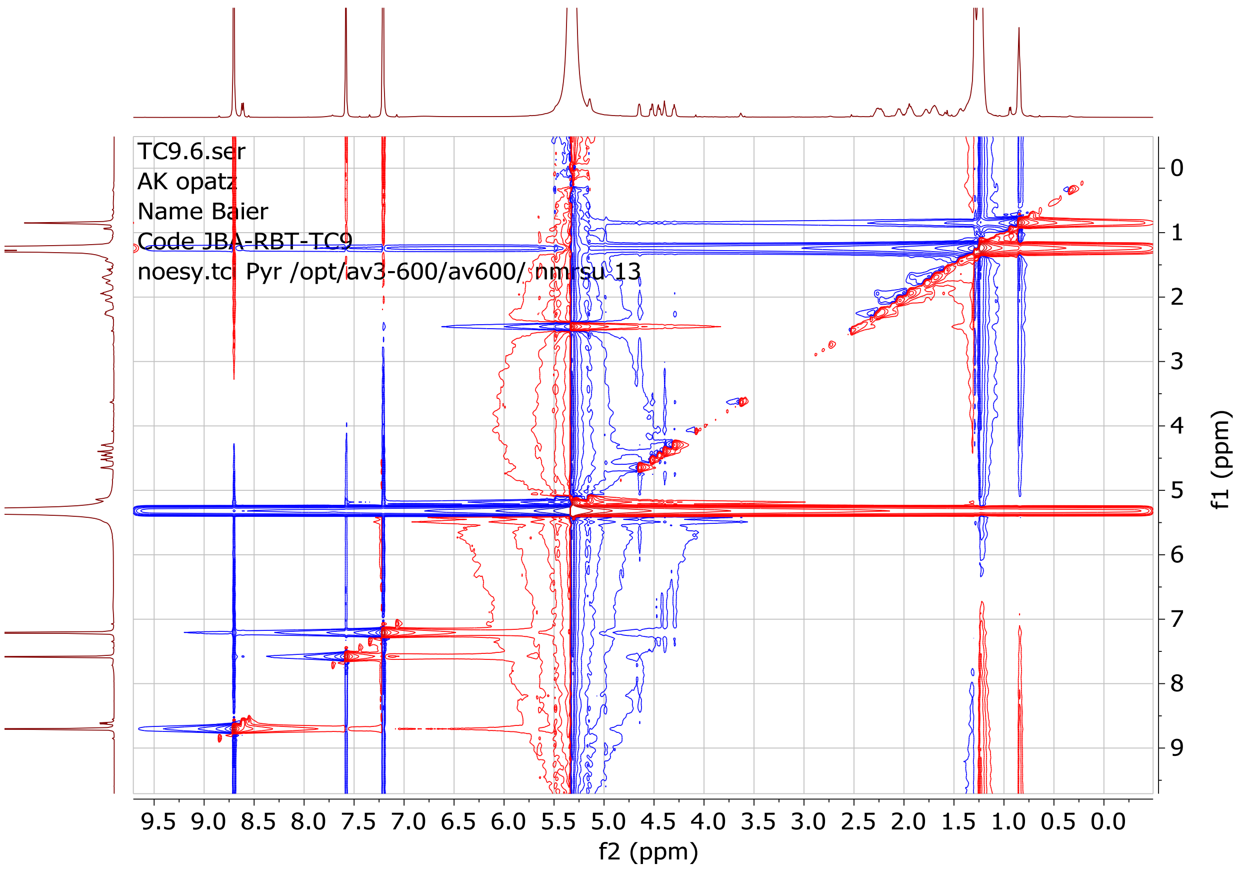

Figure S15. NOESY spectrum of compound **2**

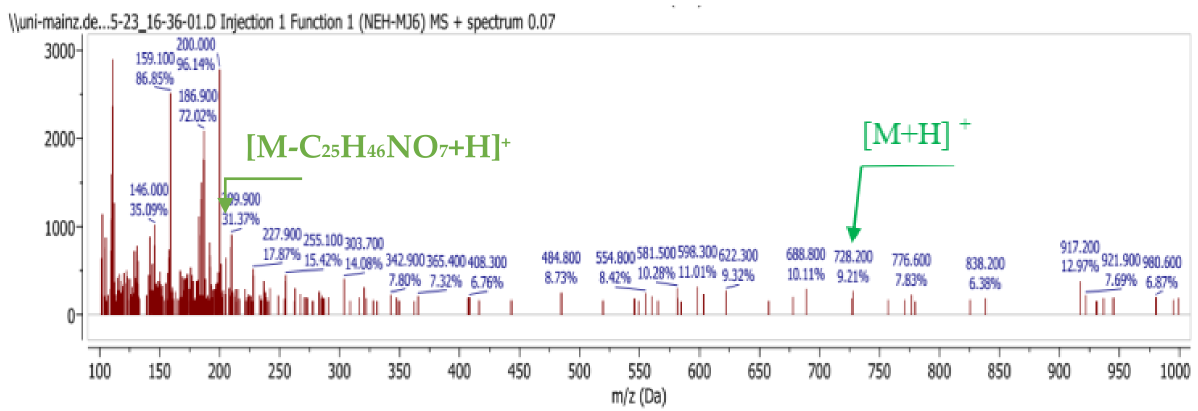

Figure S16. ESI-MS of compound **3**

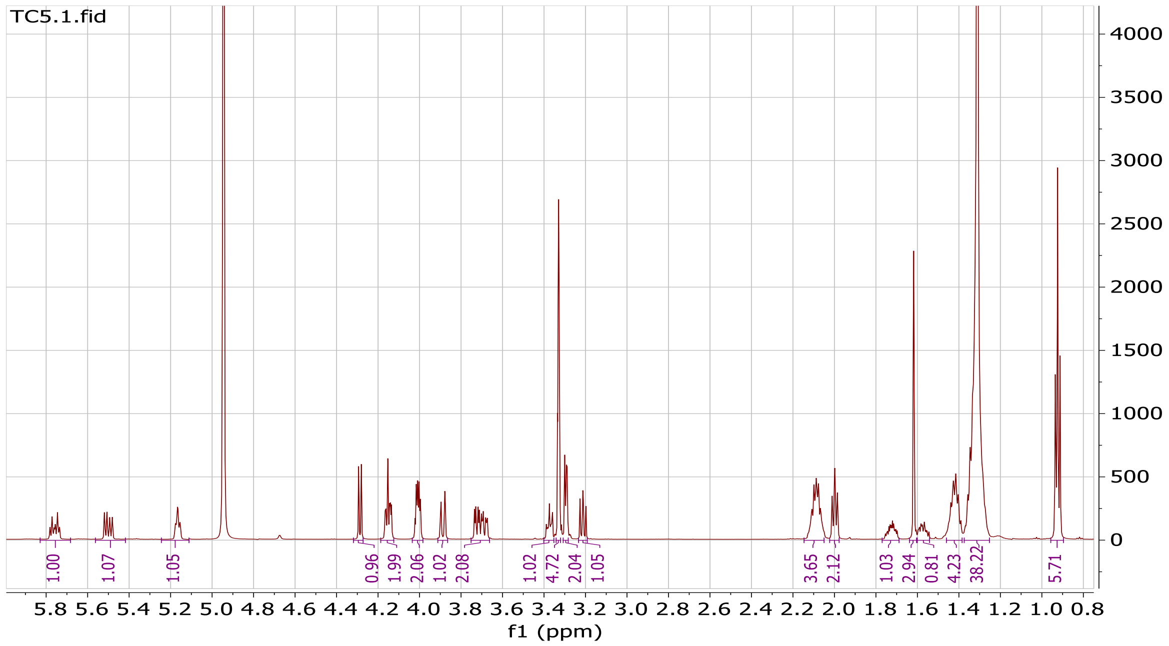

Figure S17. ^{1}H NMR spectrum (600 MHz, CD_3OD) of compound **3**

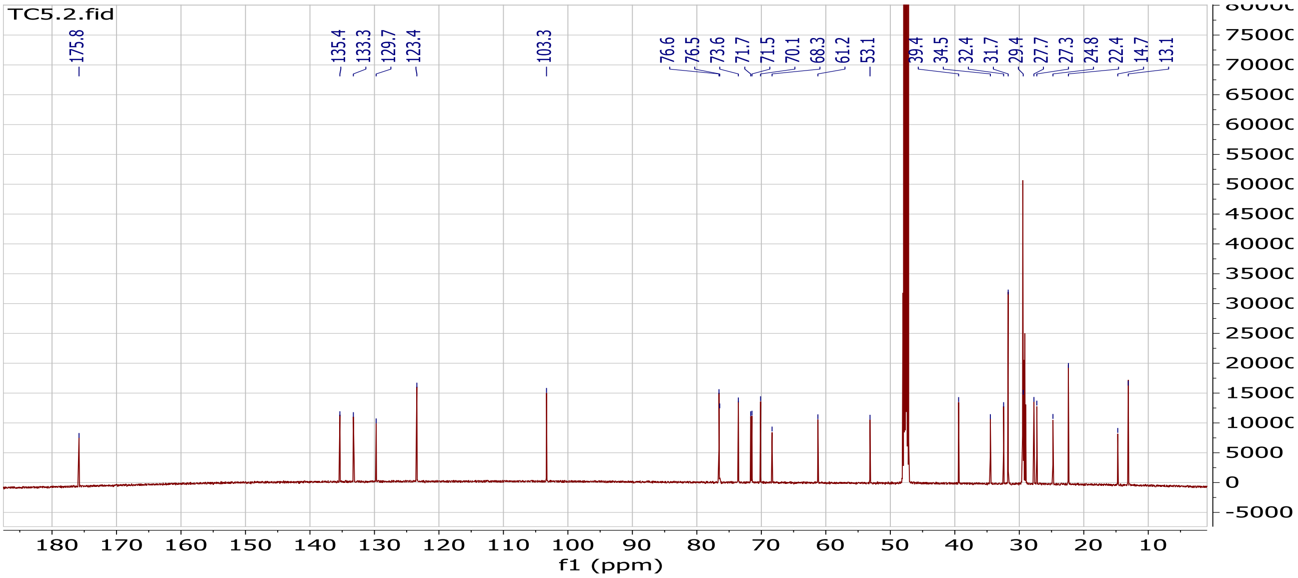

Figure S18. ^{13}C NMR spectrum (150 MHz, CD_3OD) of compound **3**

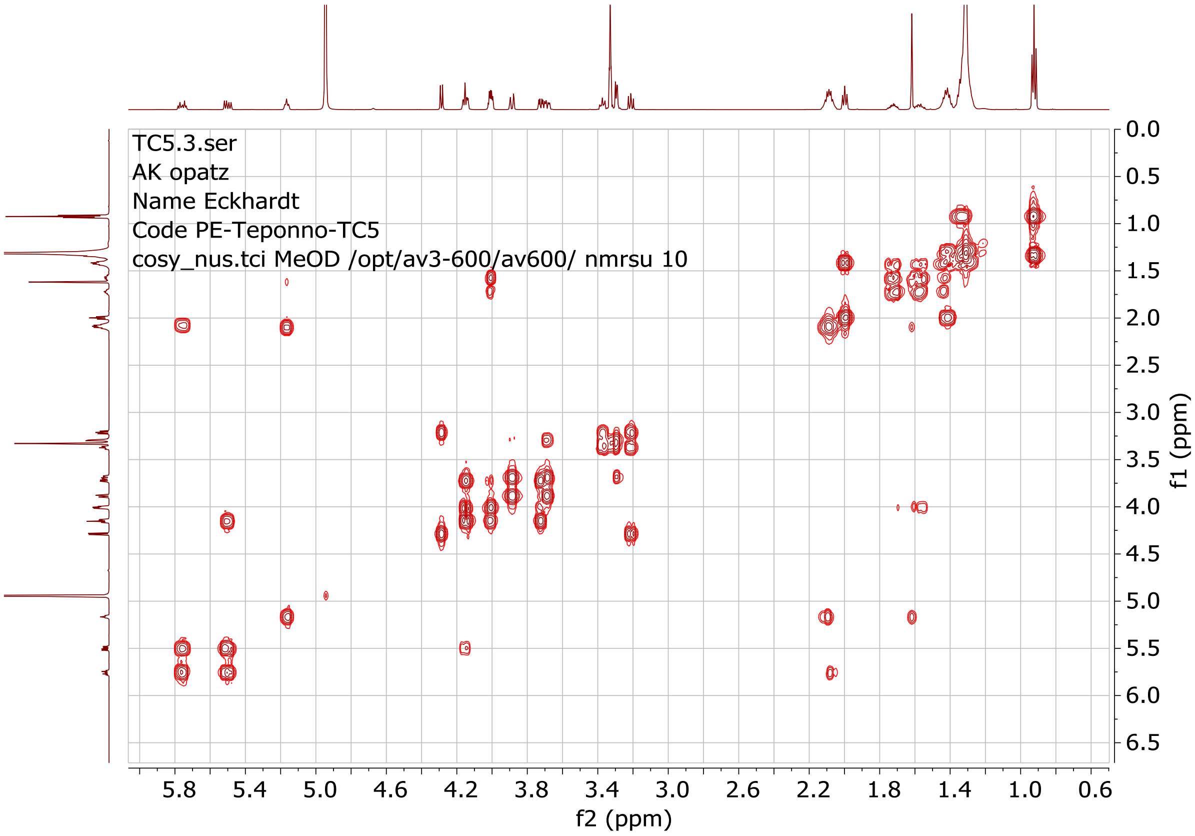

Figure S19. ^{1}H-^{1}H COSY spectrum of compound **3**

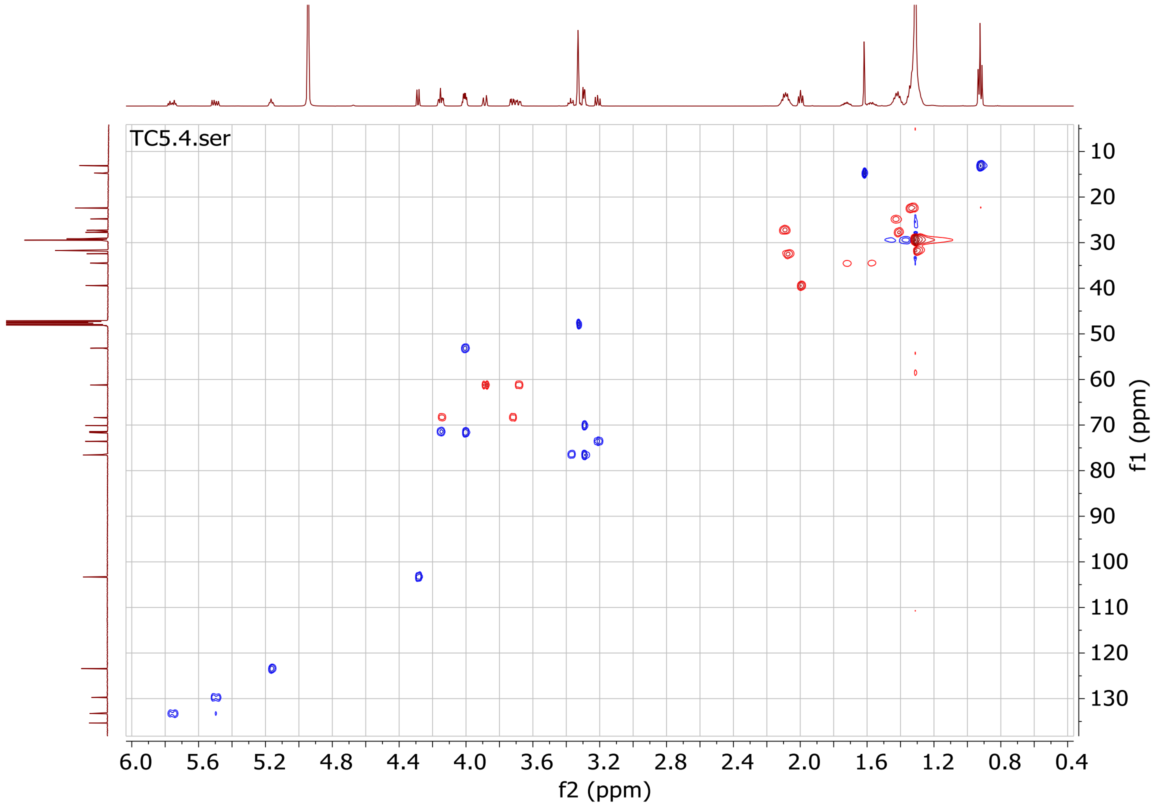

Figure S20. HSQC spectrum of compound **3**

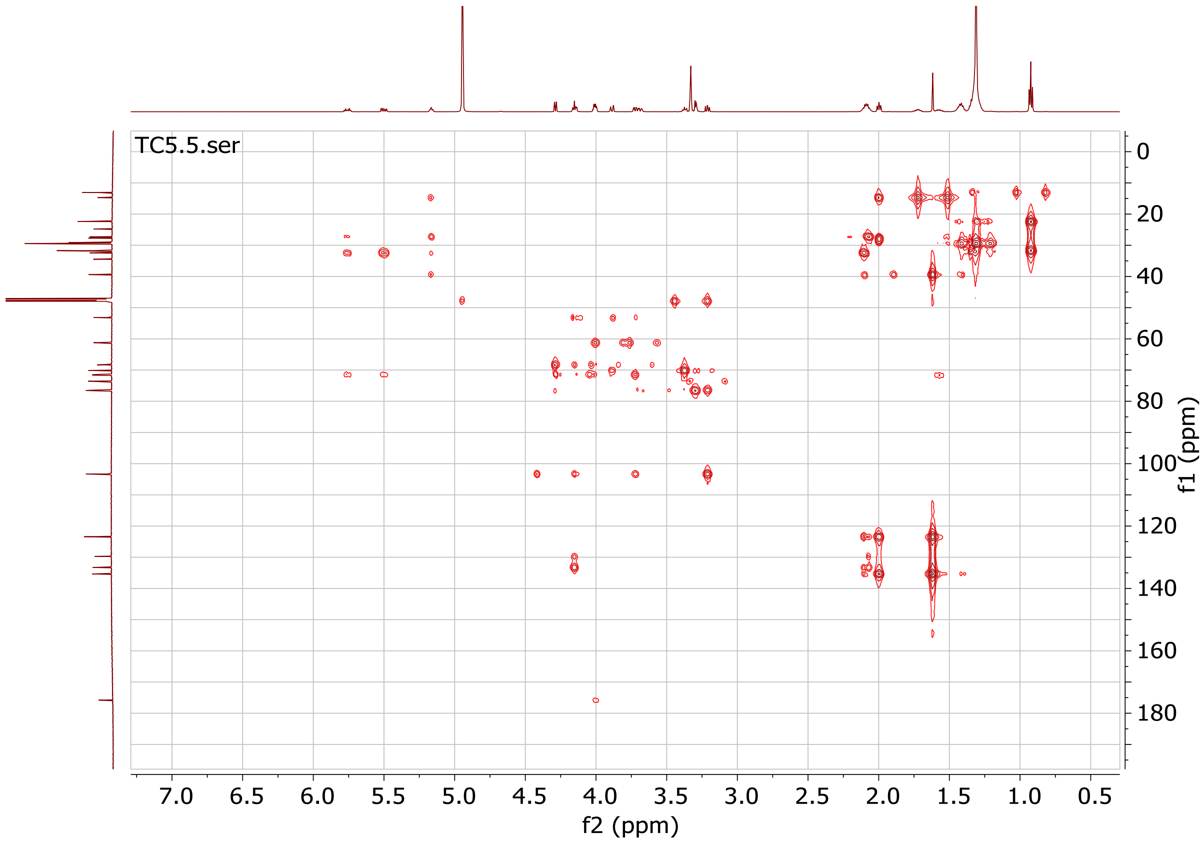

Figure S21. HMBC spectrum of compound **3**

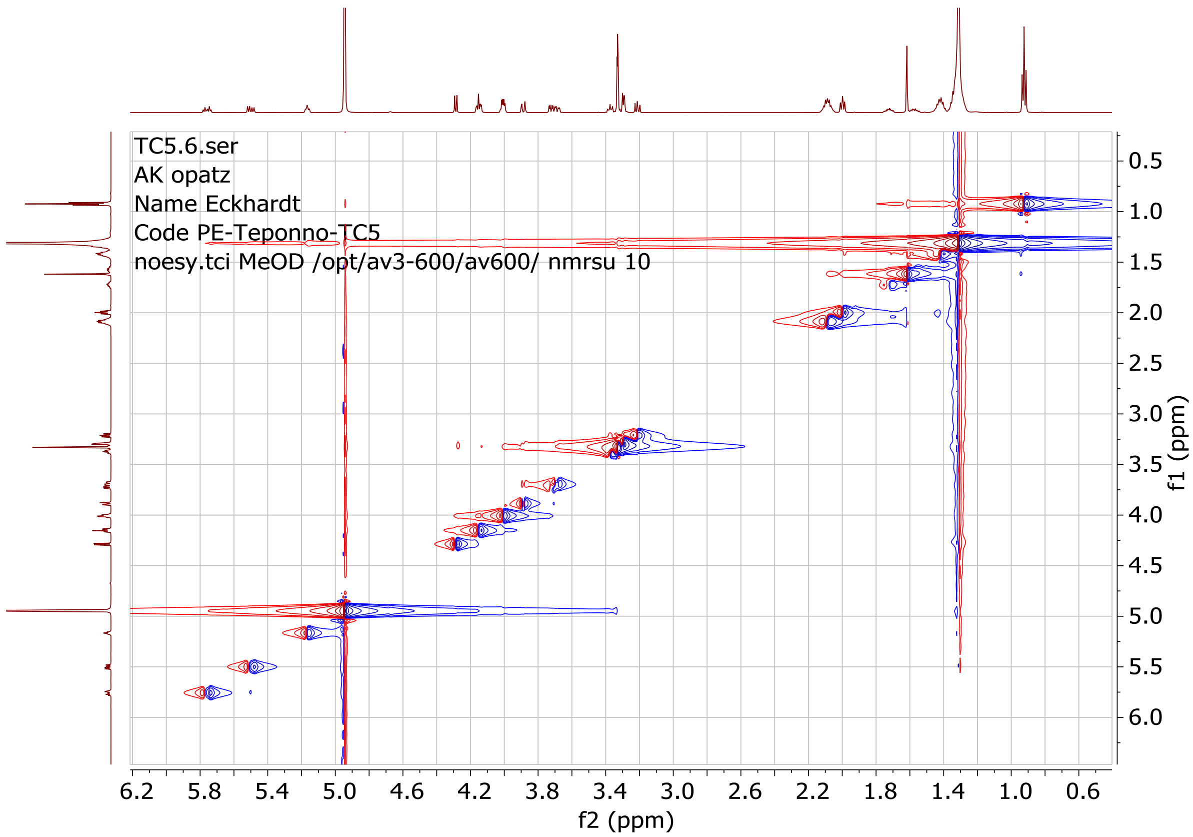

Figure S22. NOESY spectrum of compound **3**

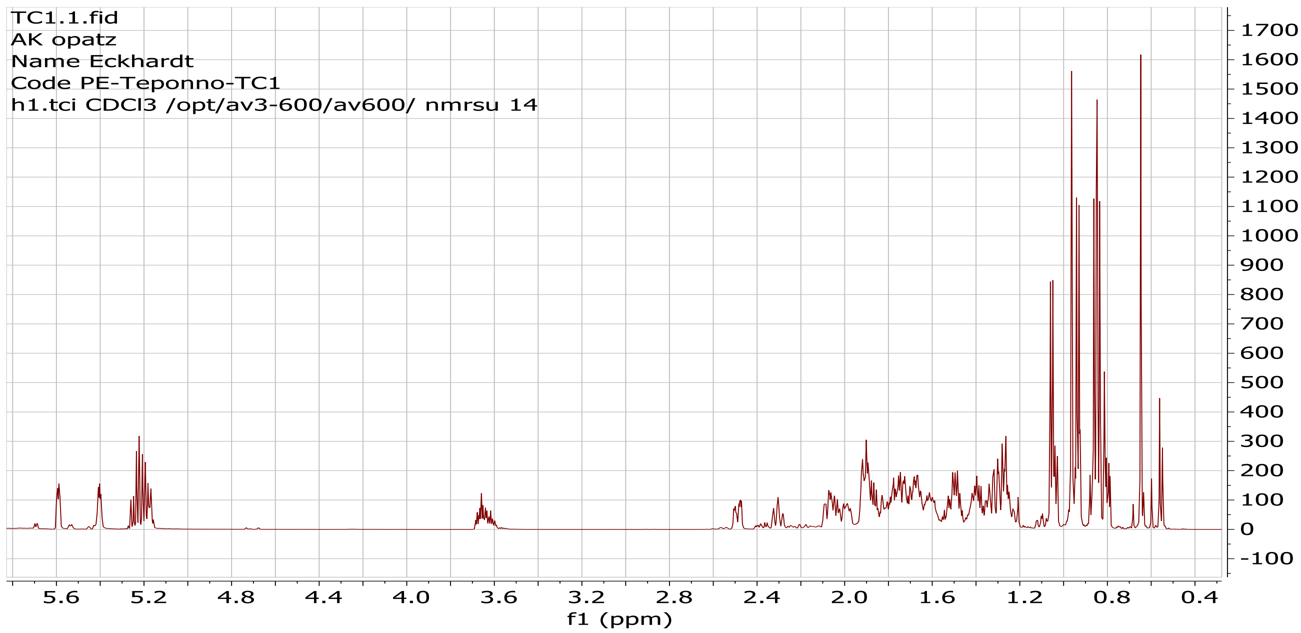

Figure S23. ^{1}H NMR spectrum (600 MHz, $CDCl_3$) of compound **4**

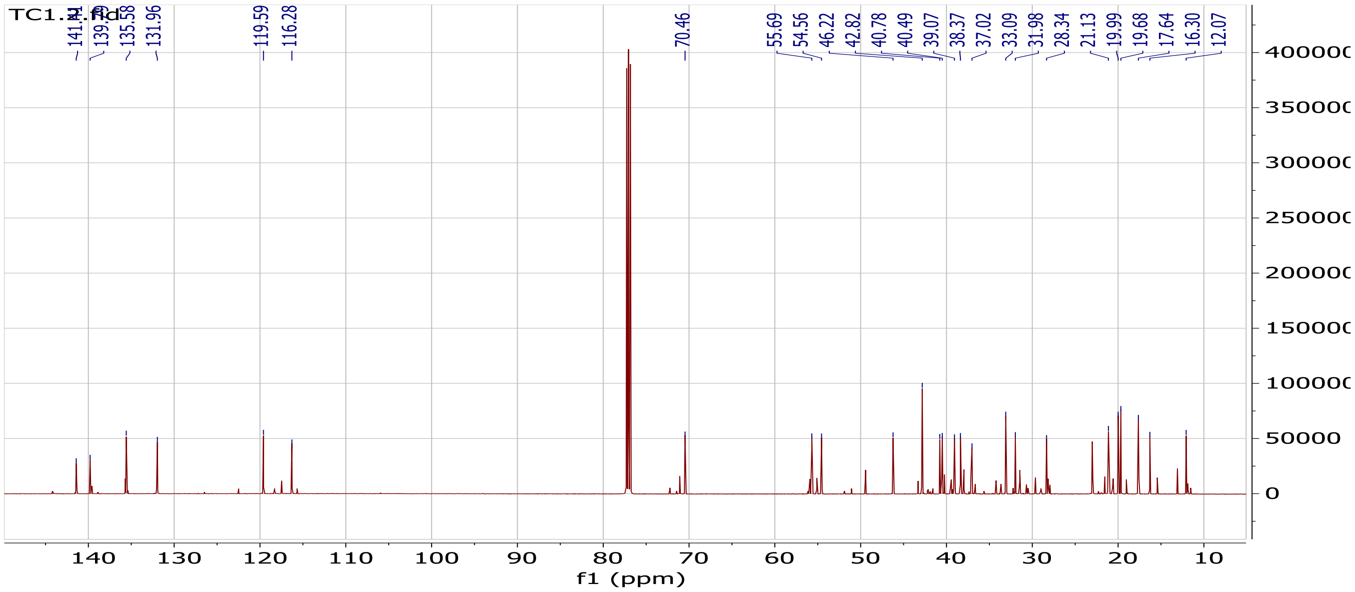

Figure S24. ^{13}C NMR spectrum (150 MHz, $CDCl_3$) of compound **4**

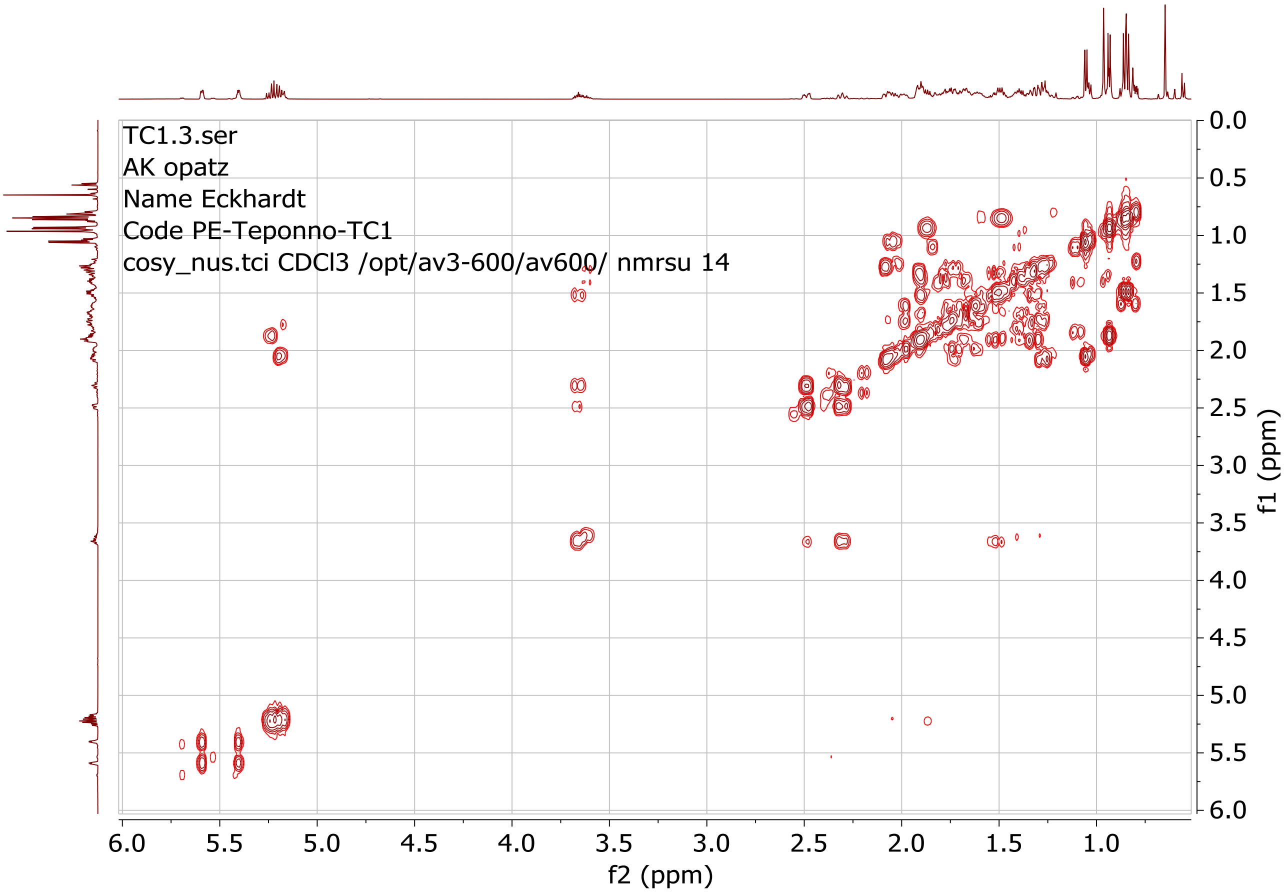

Figure S25. ^{1}H-^{1}H COSY spectrum of compound **4**

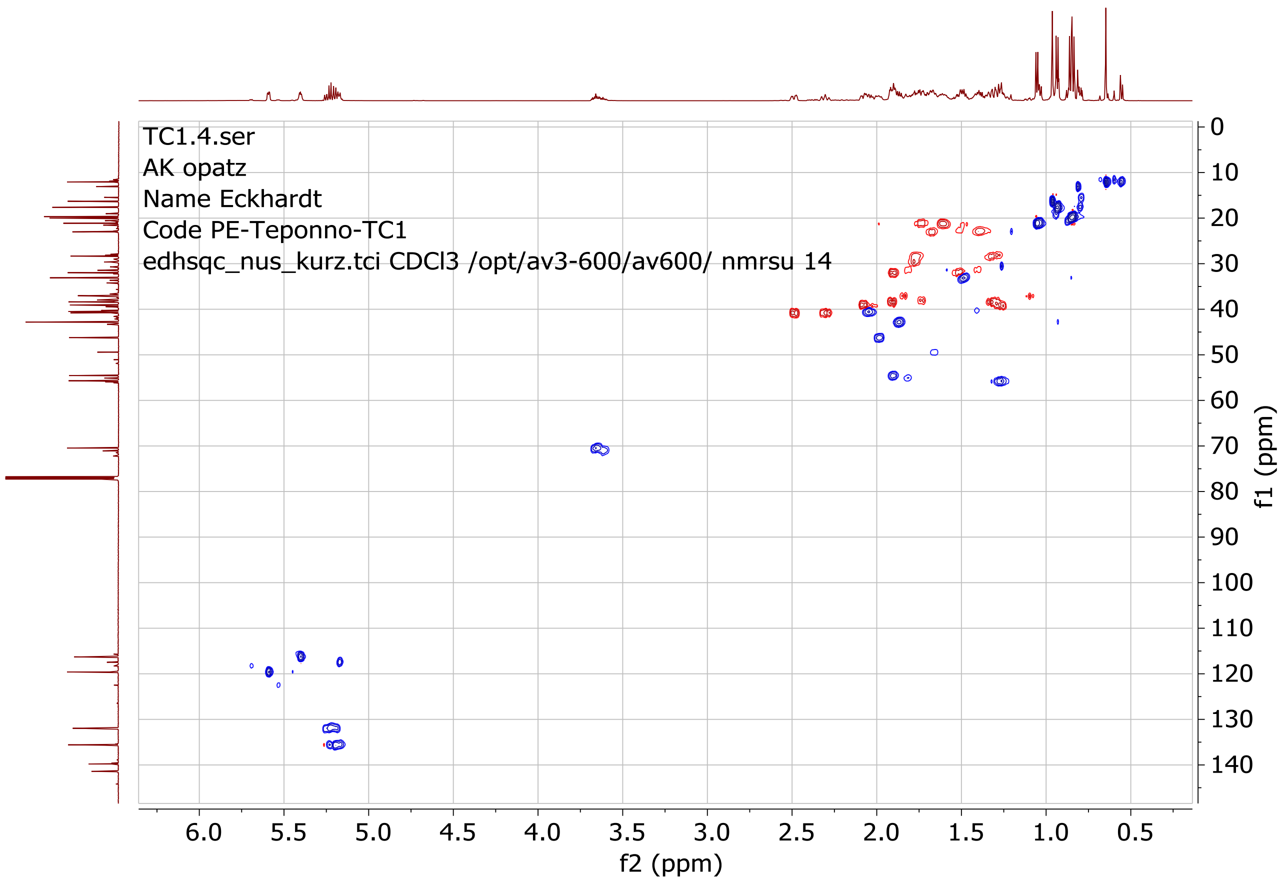

Figure S26. HSQC spectrum of compound **4**

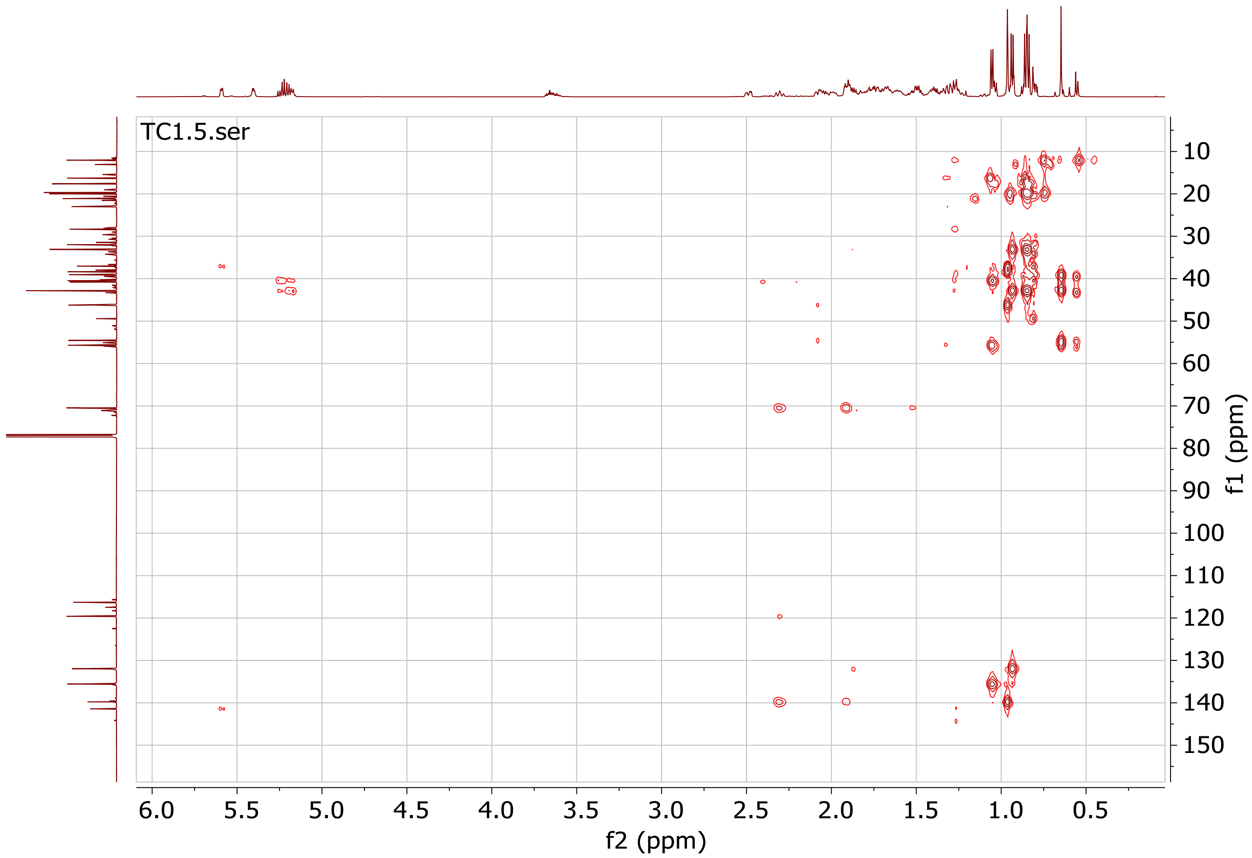

Figure S27. HMBC spectrum of compound **4**

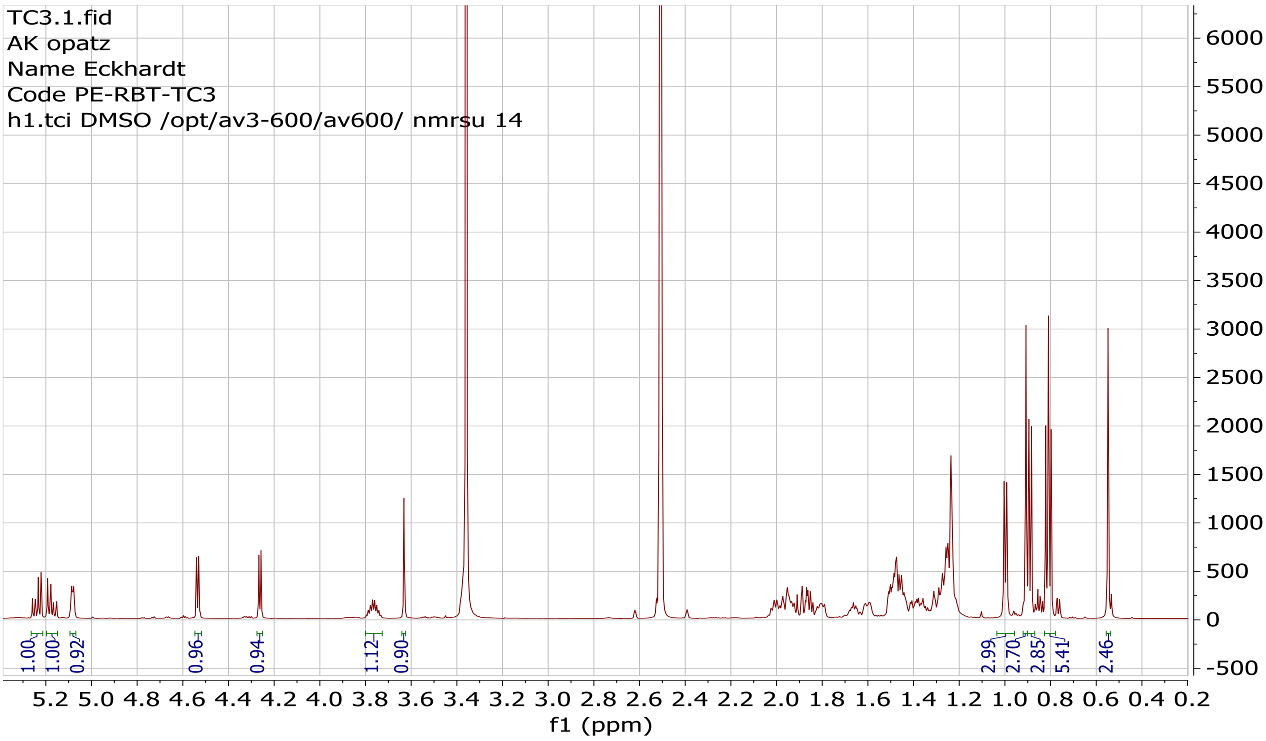

Figure S28. ^{1}H NMR spectrum (600 MHz, DMSO-d_6) of compound **5**

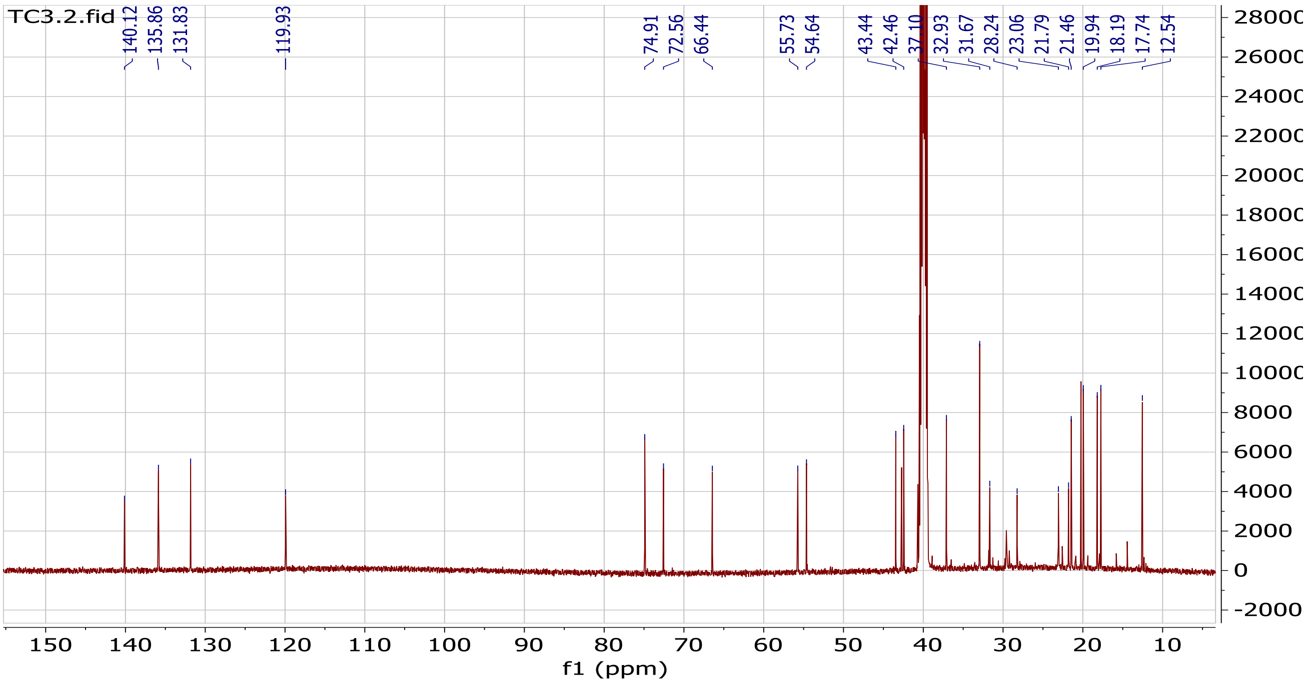

Figure S29. ^{13}C NMR spectrum (150 MHz, DMSO-d_6) of compound **5**

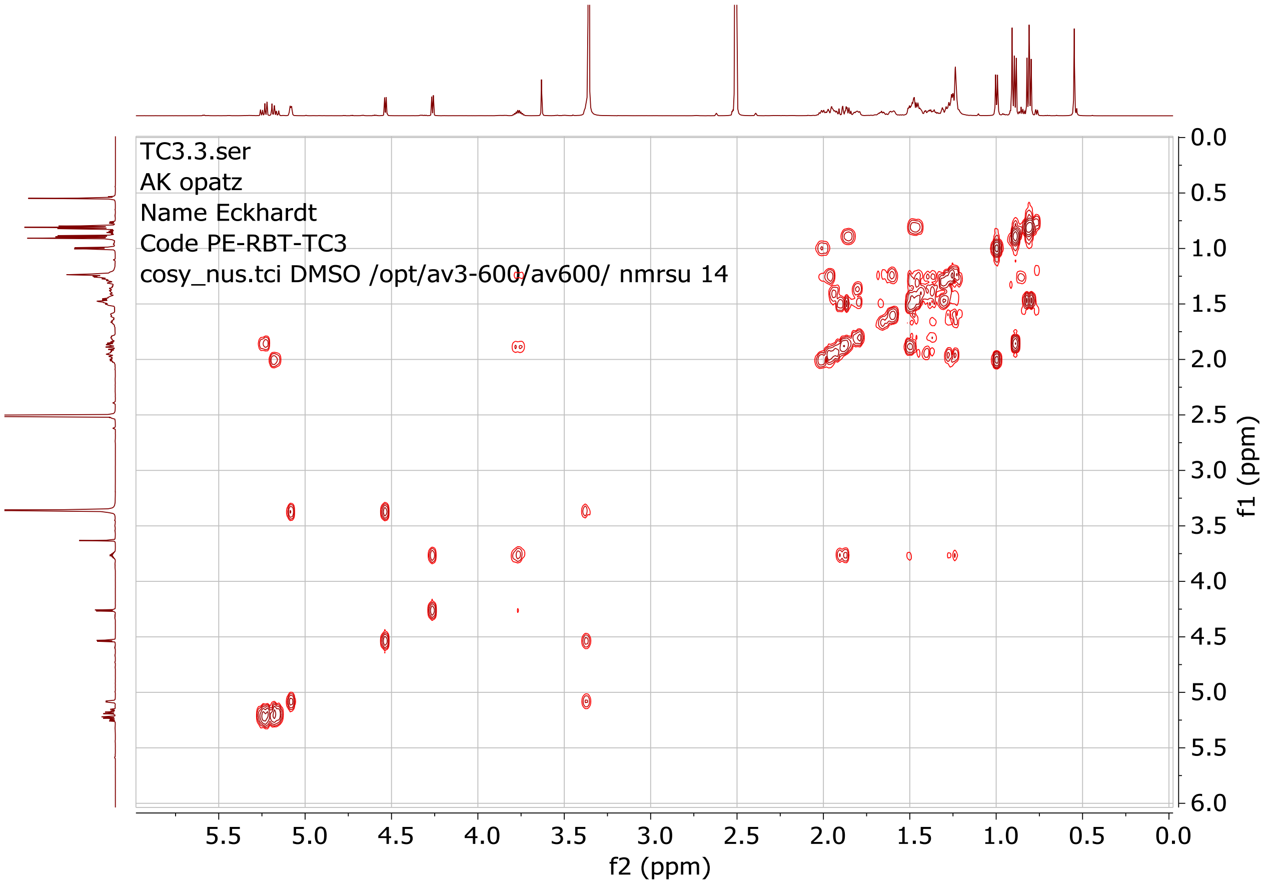

Figure S30. ^{1}H-^{1}H COSY spectrum of compound **5**

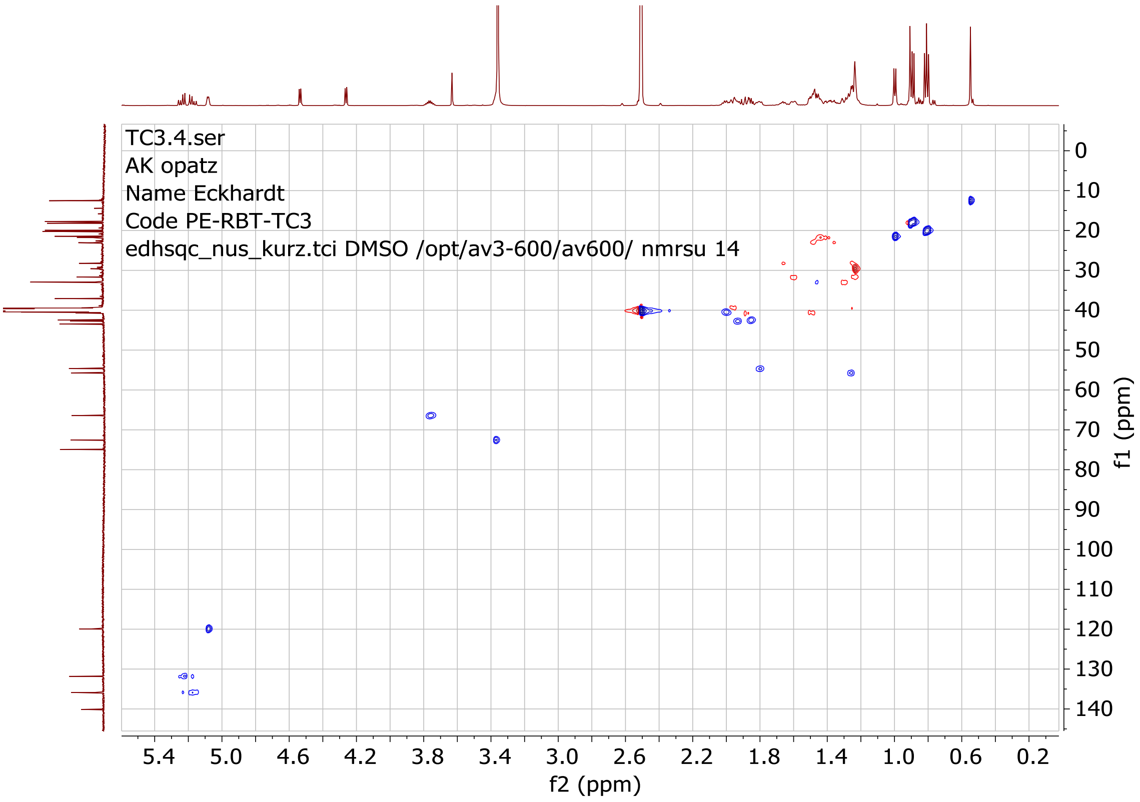

Figure S31. HSQC spectrum of compound **5**

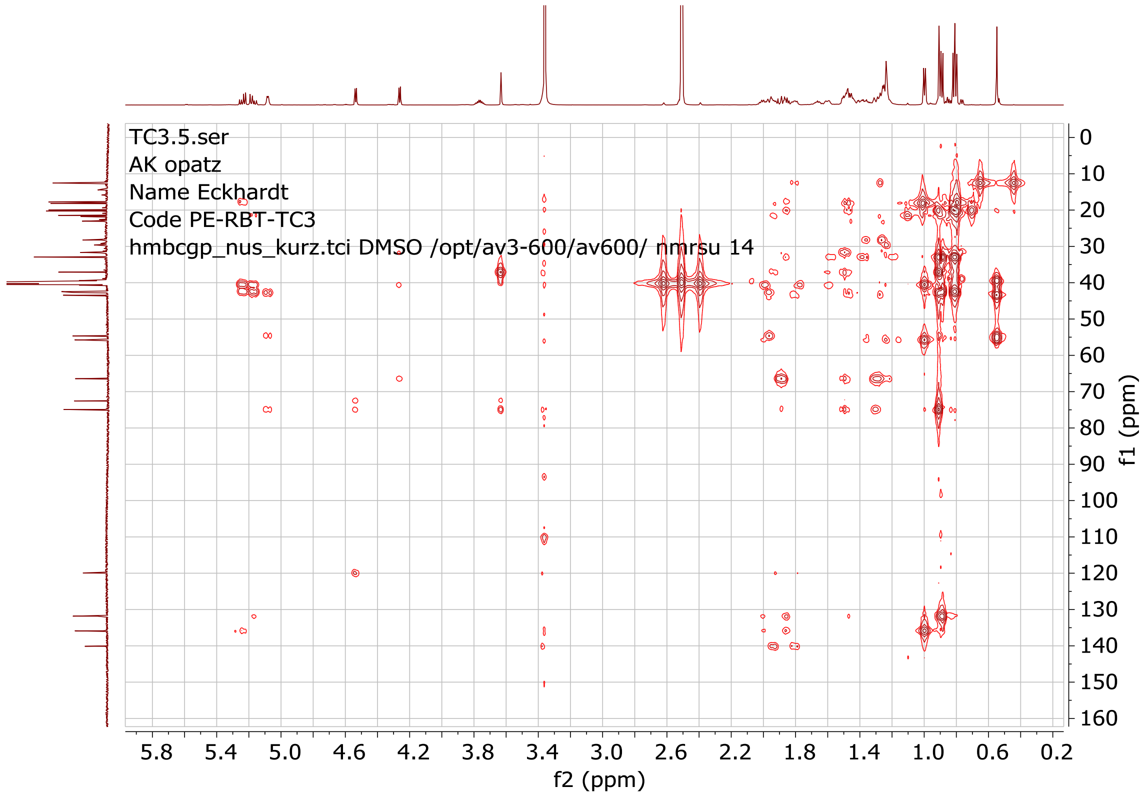

Figure S32. HMBC spectrum of compound **5**

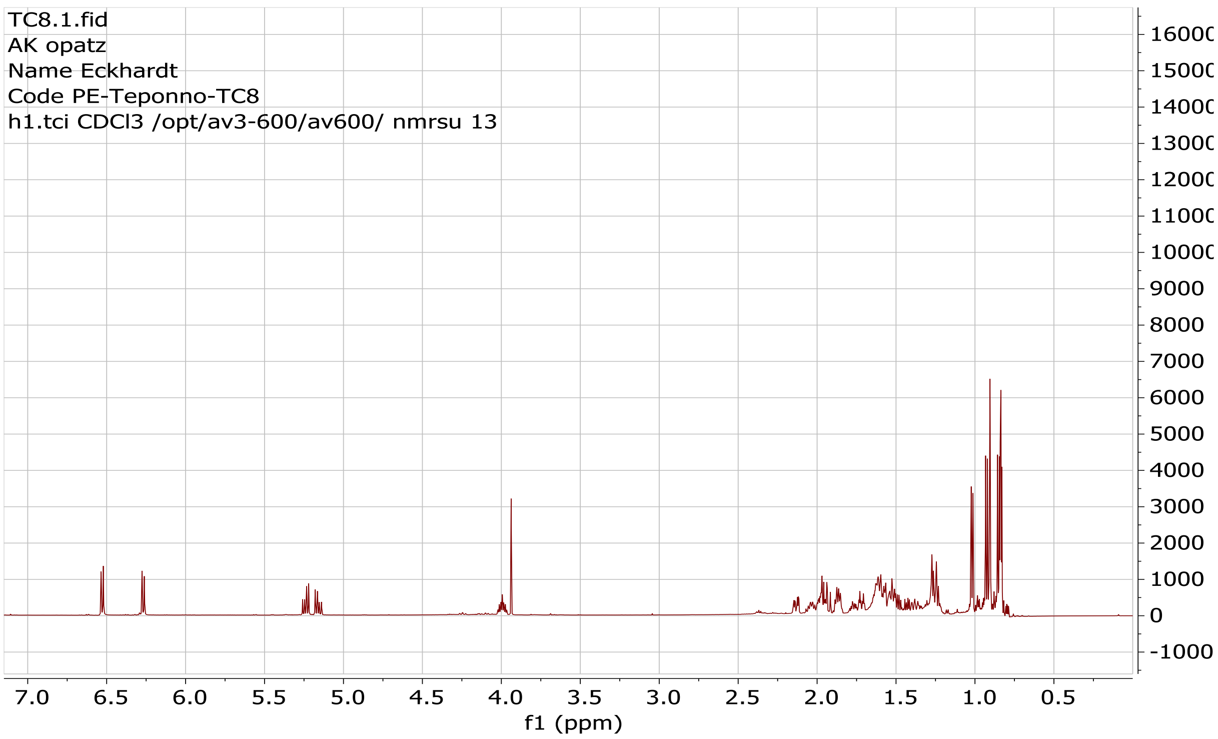

Figure S33. ^{1}H NMR spectrum (600 MHz, $CDCl_3$) of compound **6**

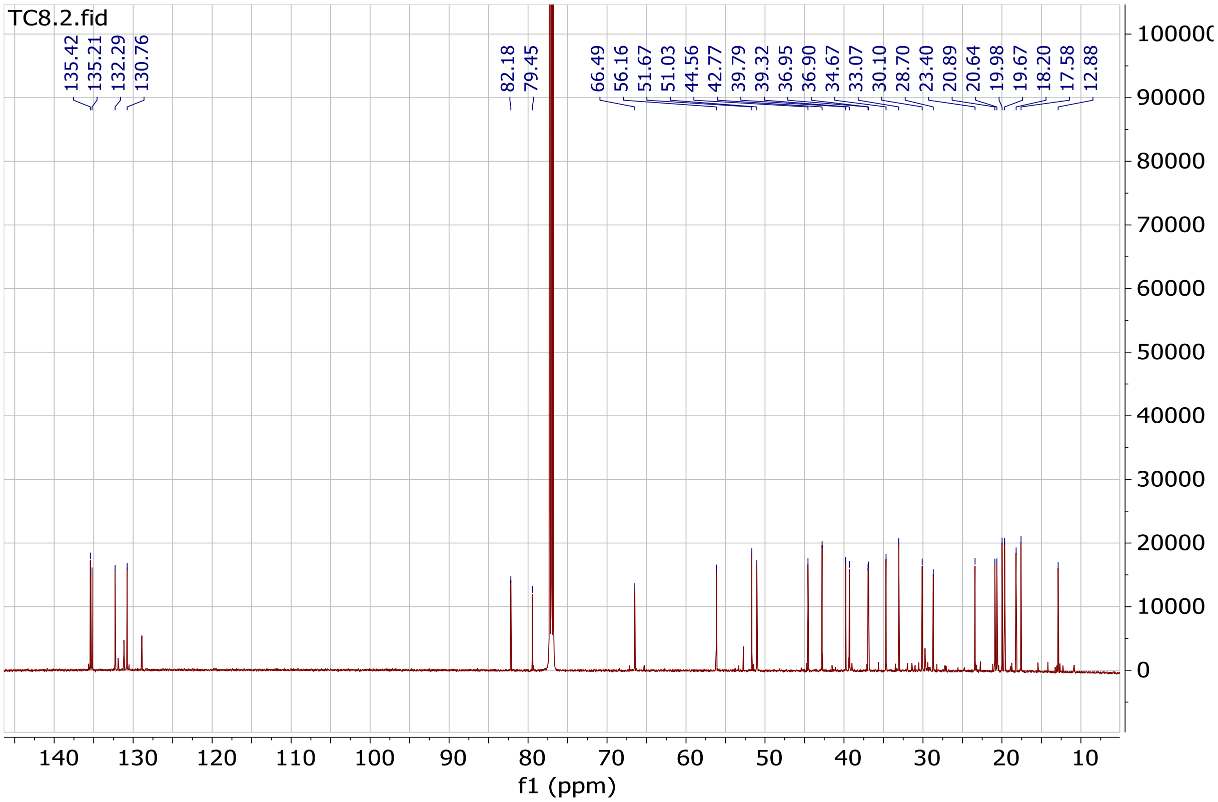

Figure S34. ^{13}C NMR spectrum (150 MHz, $CDCl_3$) of compound **6**

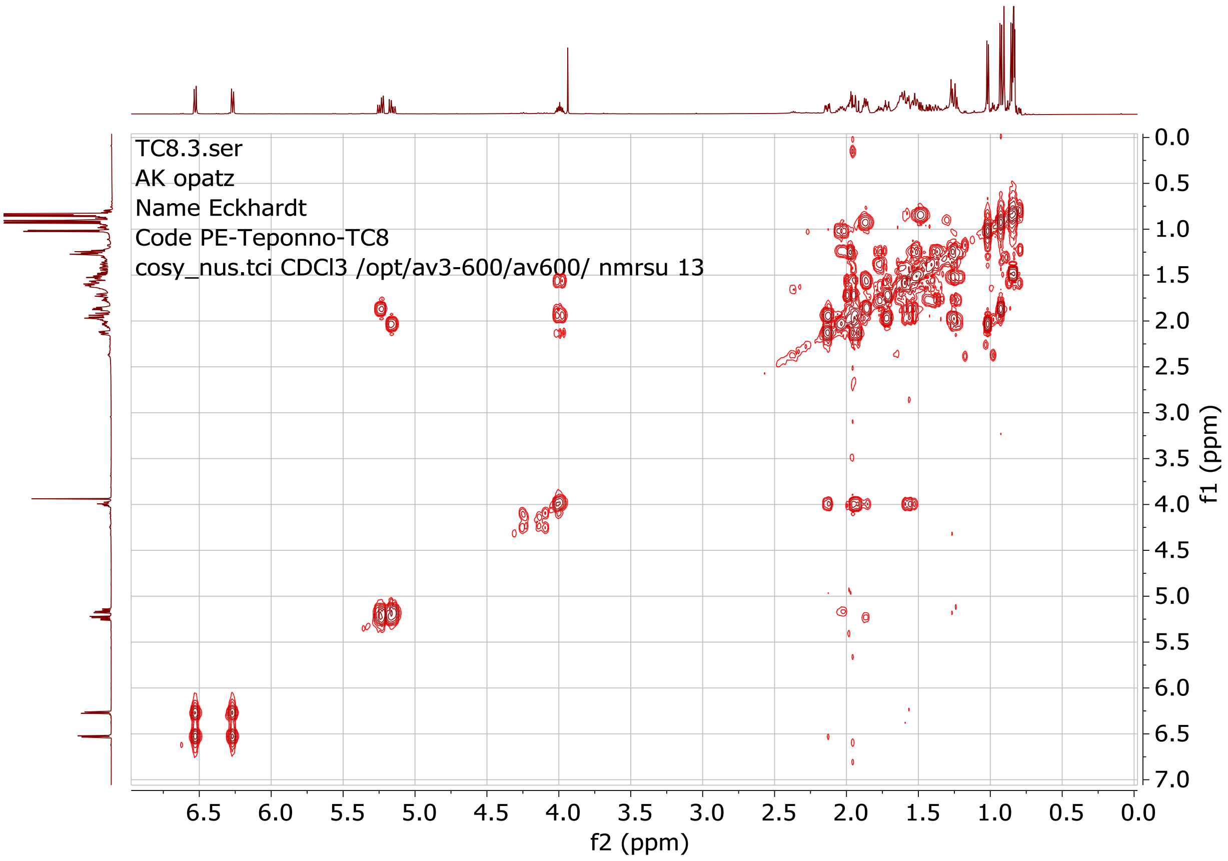

Figure S35. ^{1}H-^{1}H COSY spectrum of compound **6**

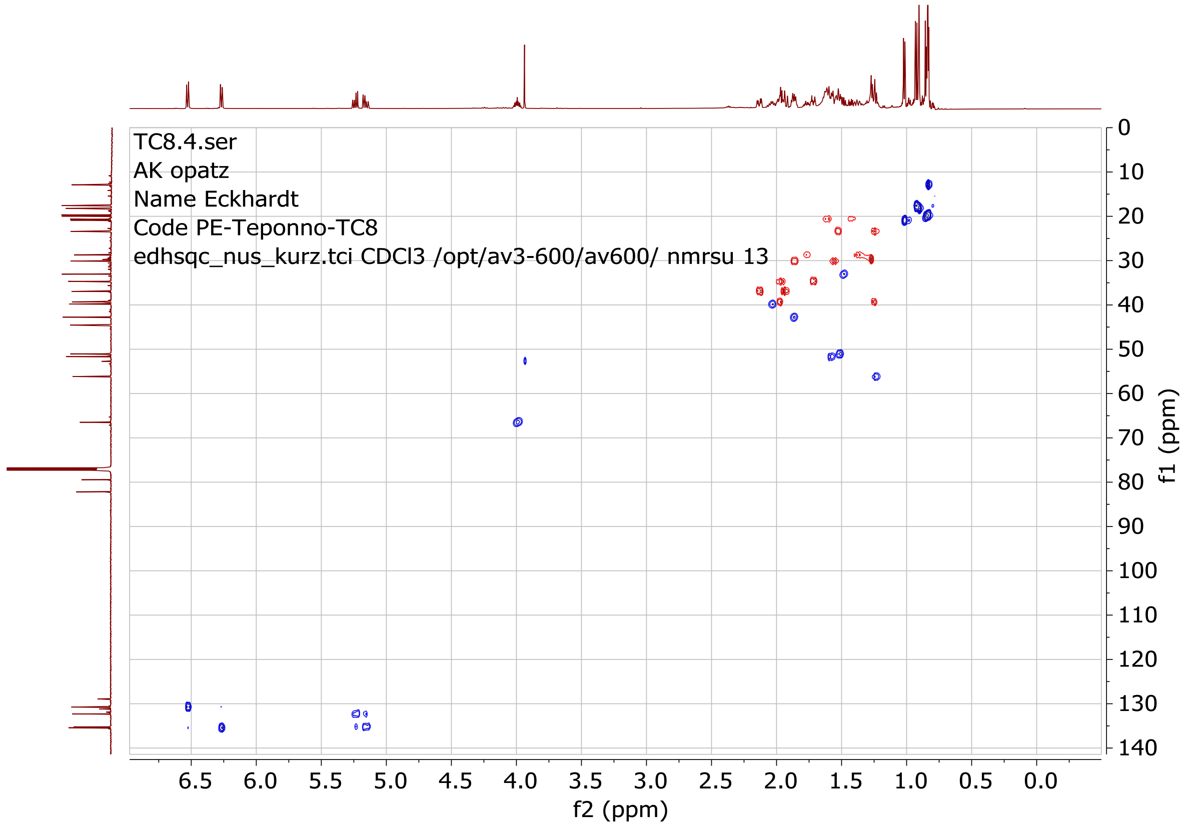

Figure S36. HSQC spectrum of compound **6**

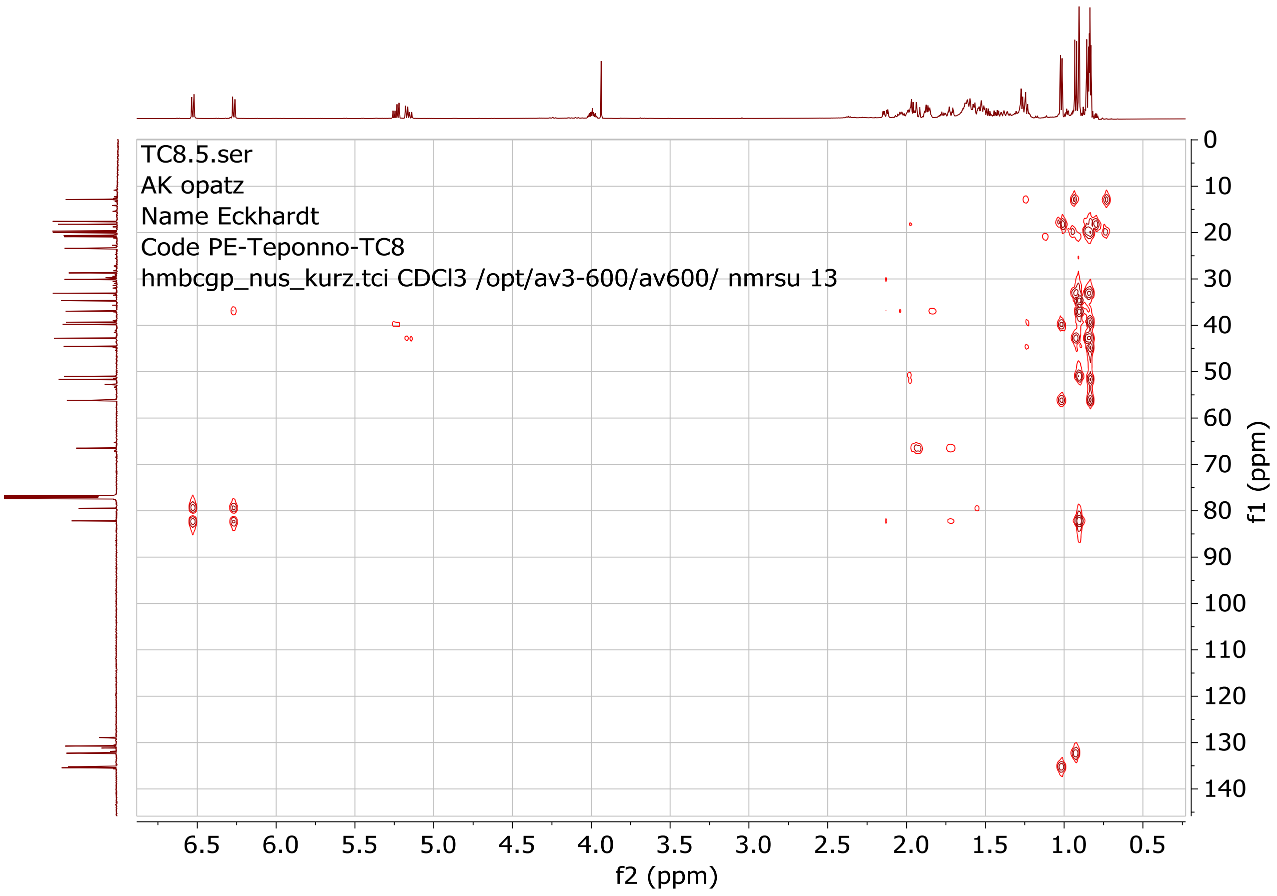

Figure S37. HMBC spectrum of compound **6**

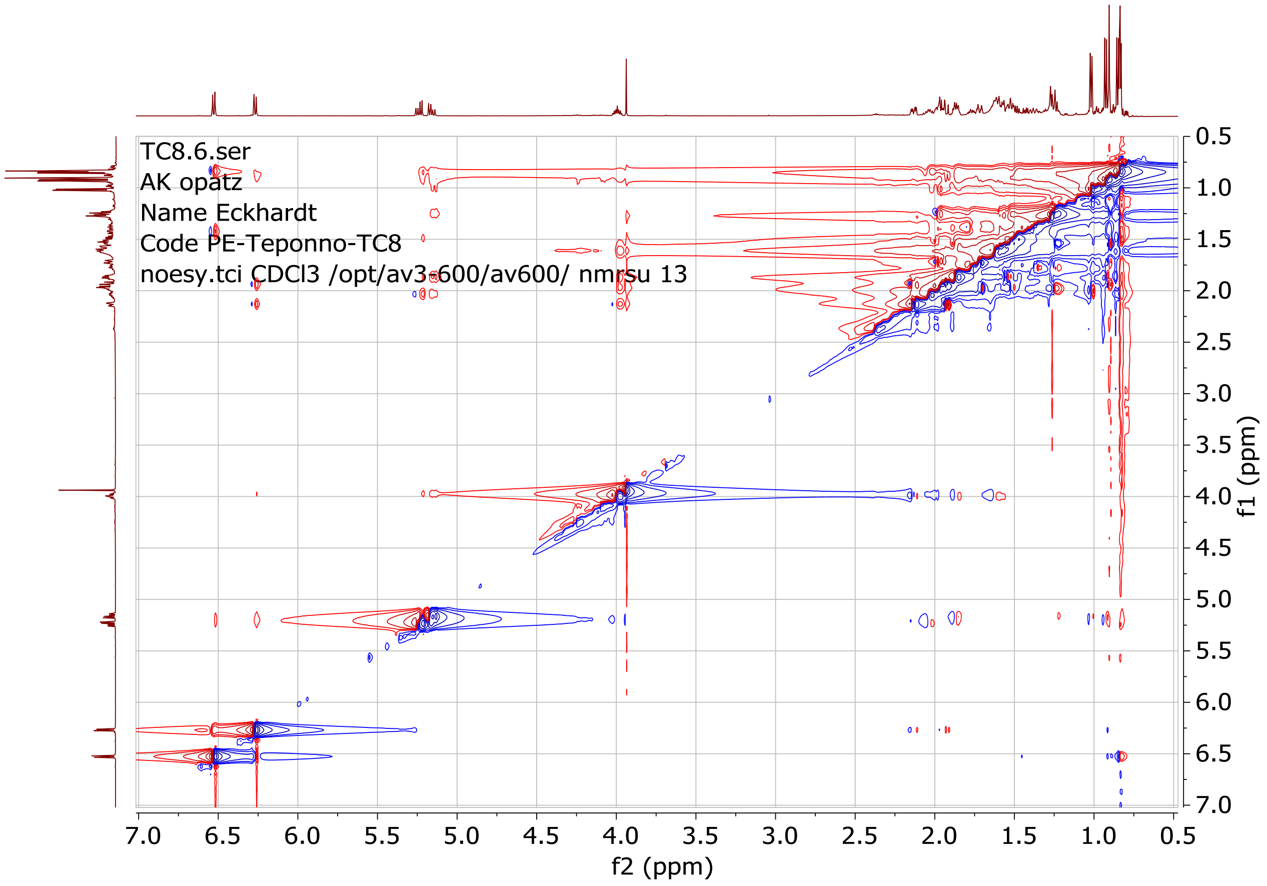

Figure S38. NOESY spectrum of compound **6**

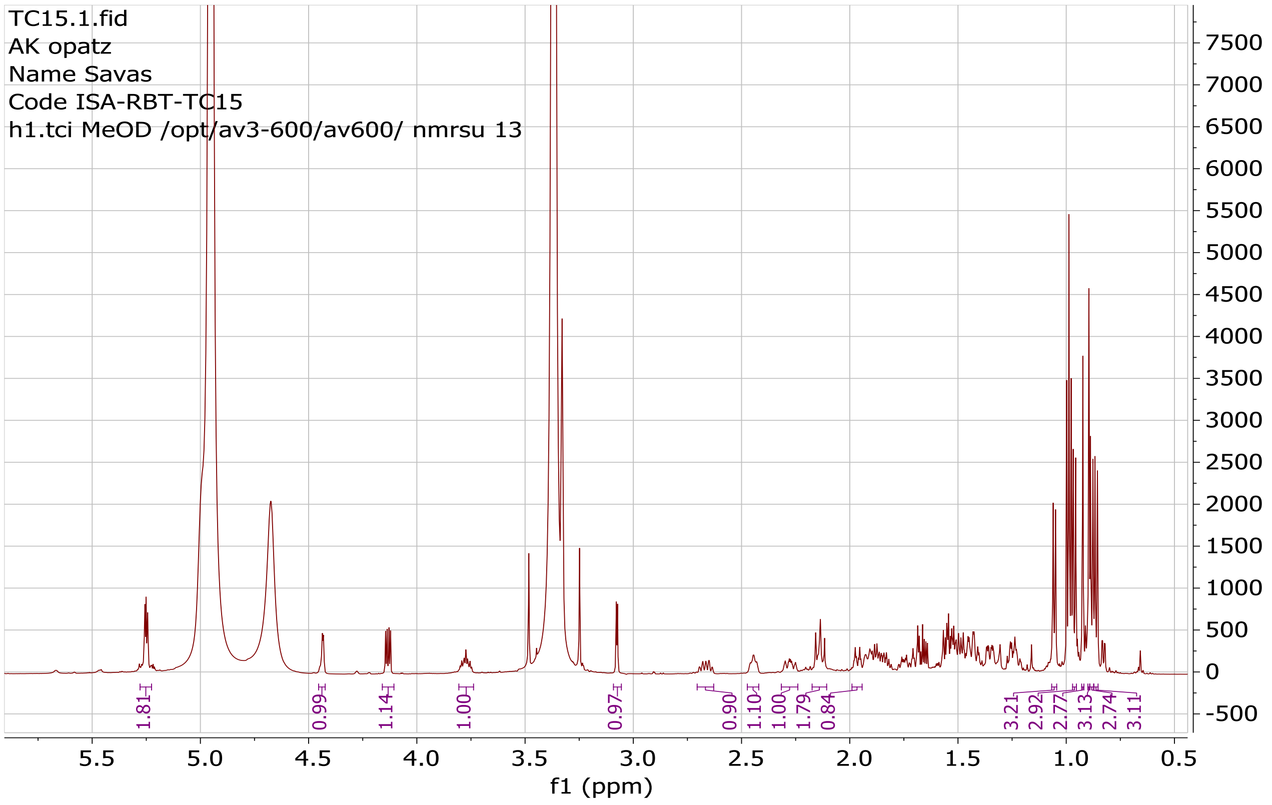

Figure S39. ^{1}H NMR spectrum (600 MHz, CD_3OD) of compound **7**

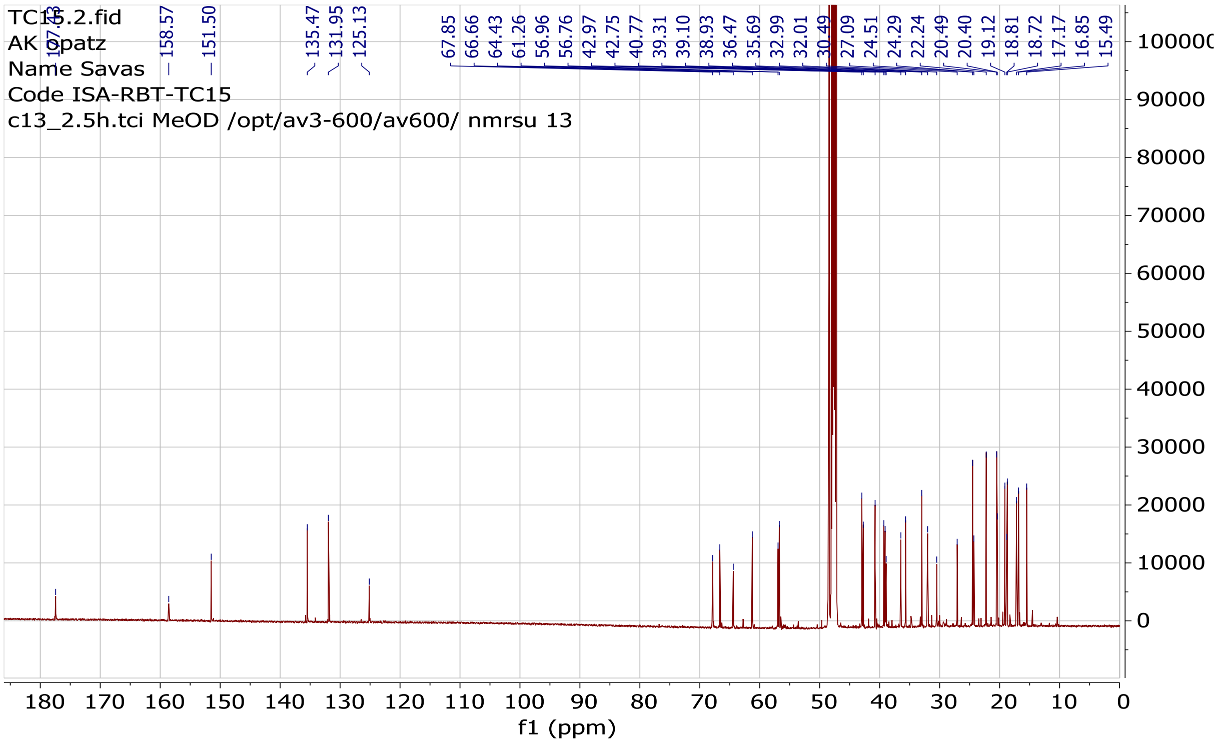

Figure S40. ^{13}C NMR spectrum (150 MHz, CD_3OD) of compound **7**

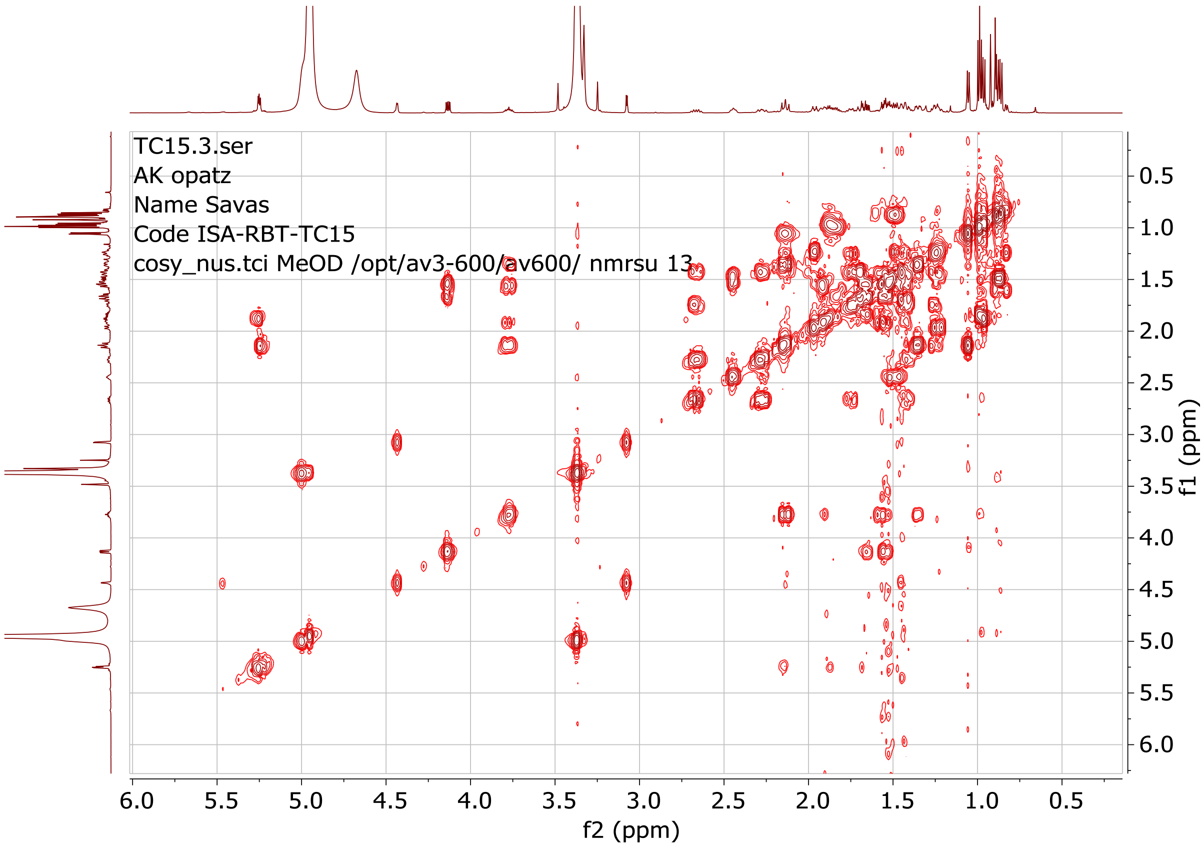

Figure S41. ^{1}H-^{1}H COSY spectrum of compound **7**

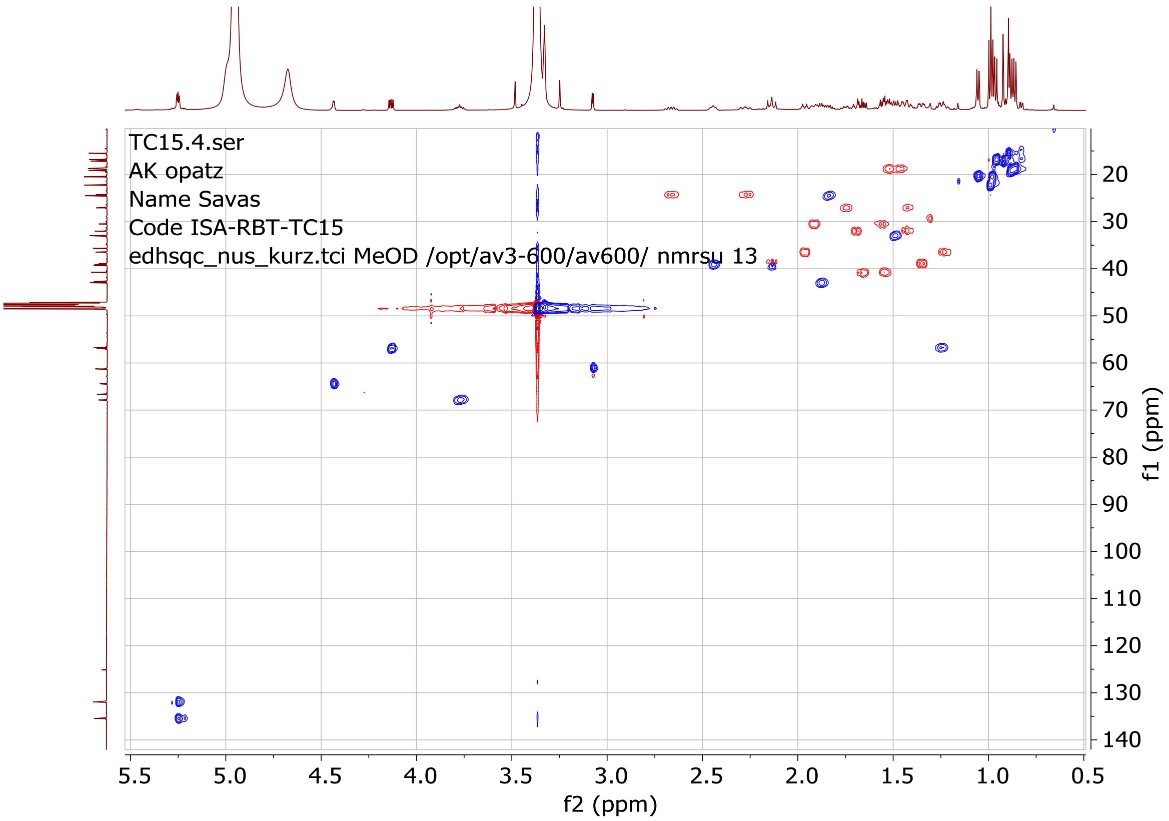

Figure S42. HSQC spectrum of compound **7**

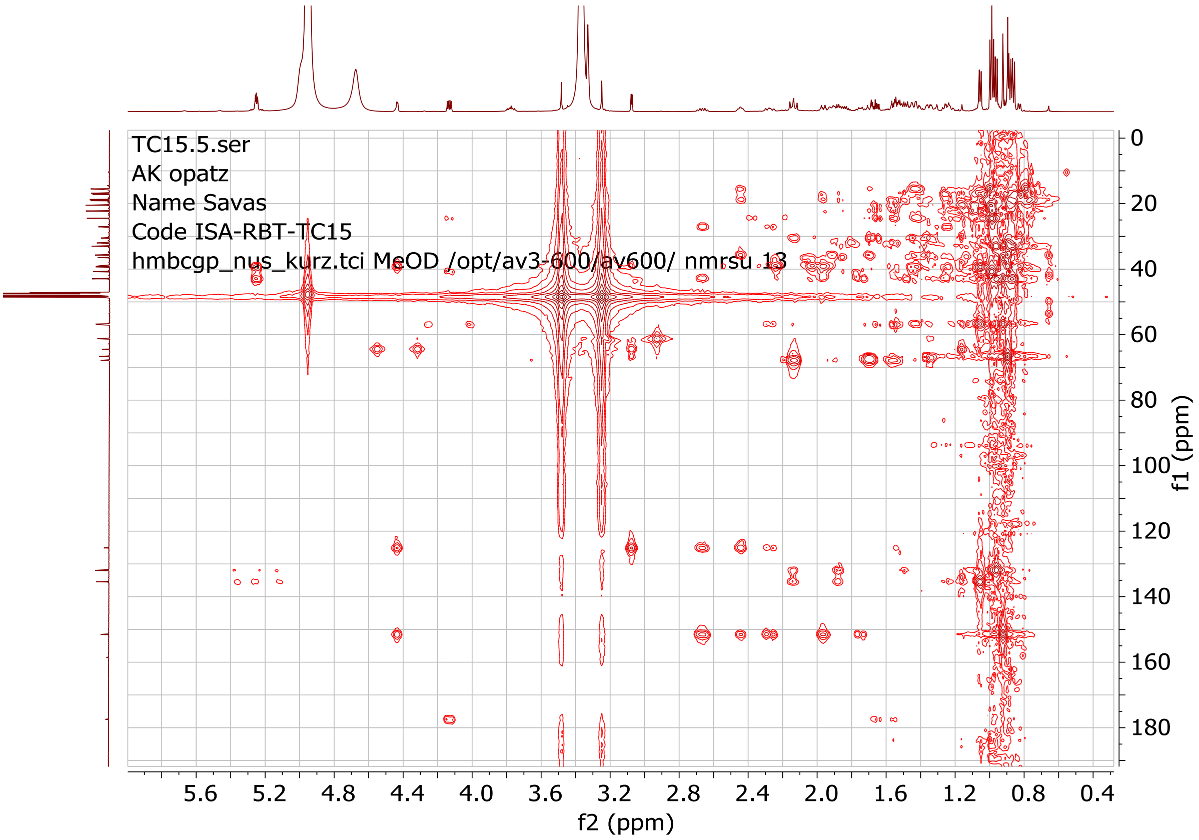

Figure S43. HMBC spectrum of compound **7**

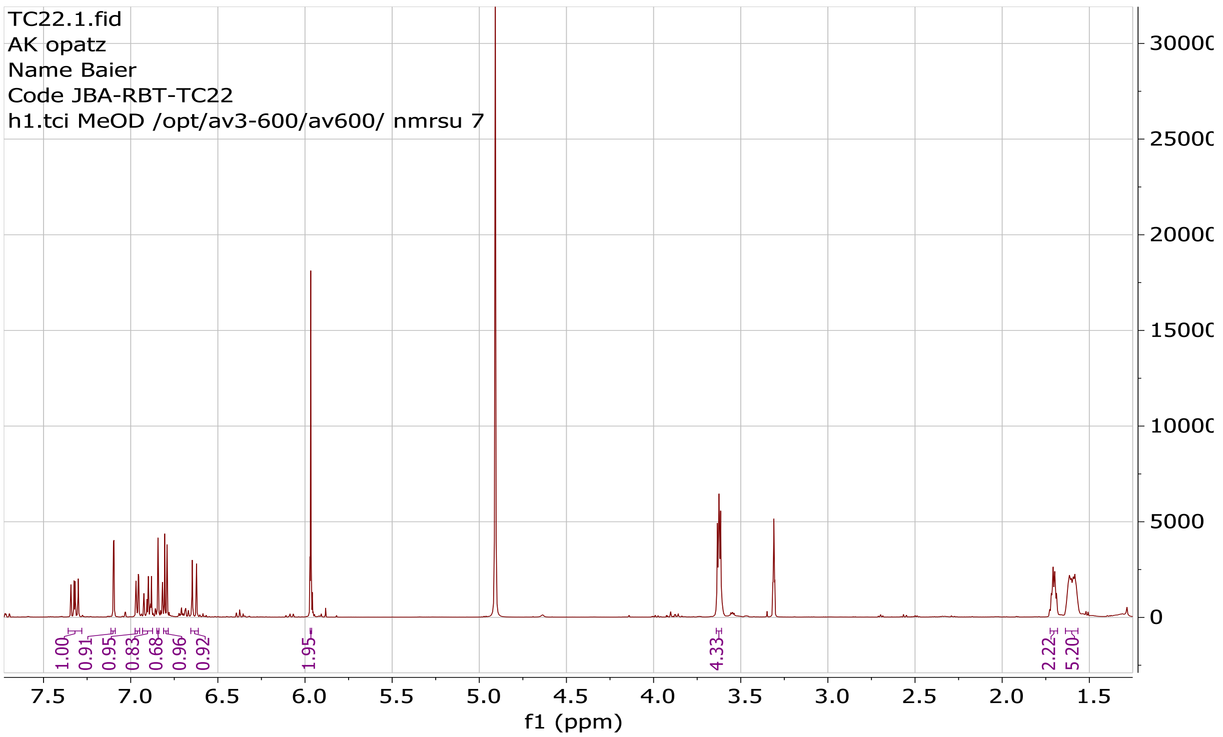

Figure S44. ^{1}H NMR spectrum (600 MHz, CD_3OD) of compound **8**

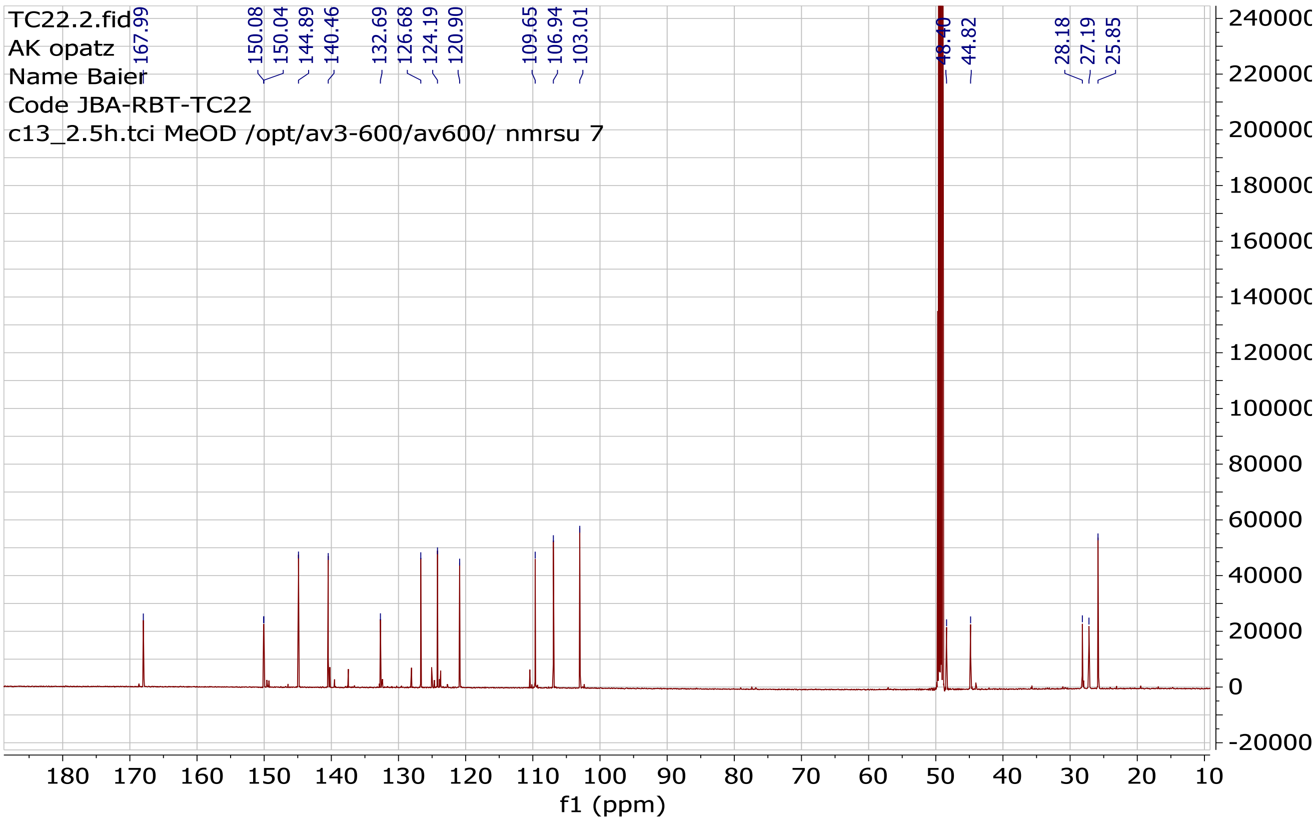

Figure S45. ^{13}C NMR spectrum (150 MHz, CD_3OD) of compound **8**

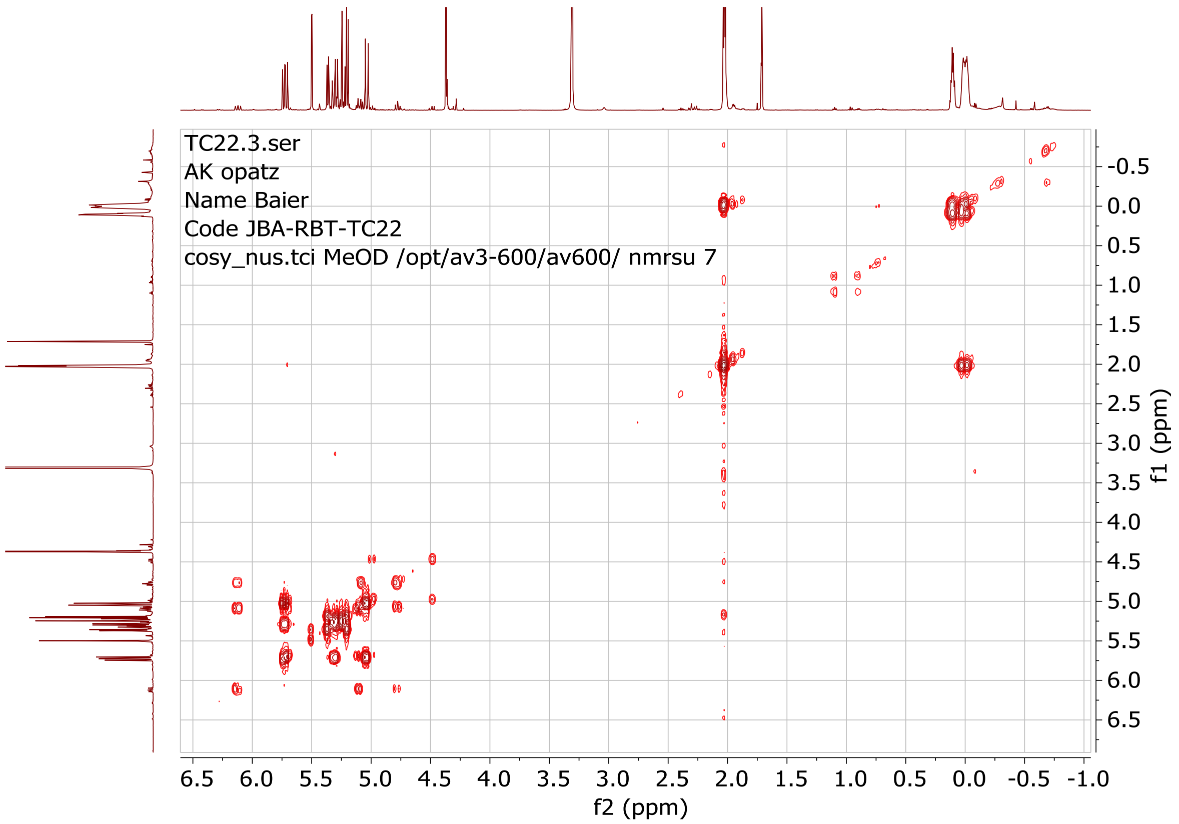

Figure S46. ^{1}H-^{1}H COSY spectrum of compound **8**

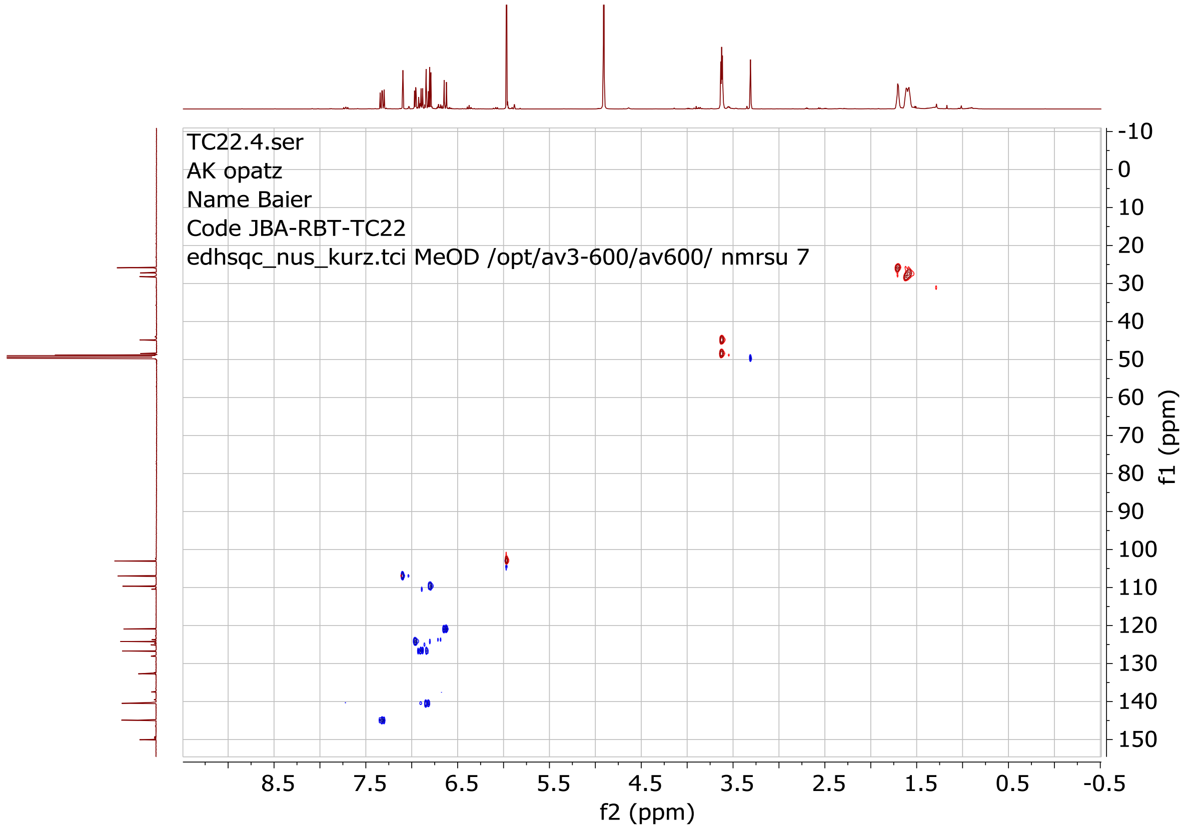

Figure S47. HSQC spectrum of compound **8**

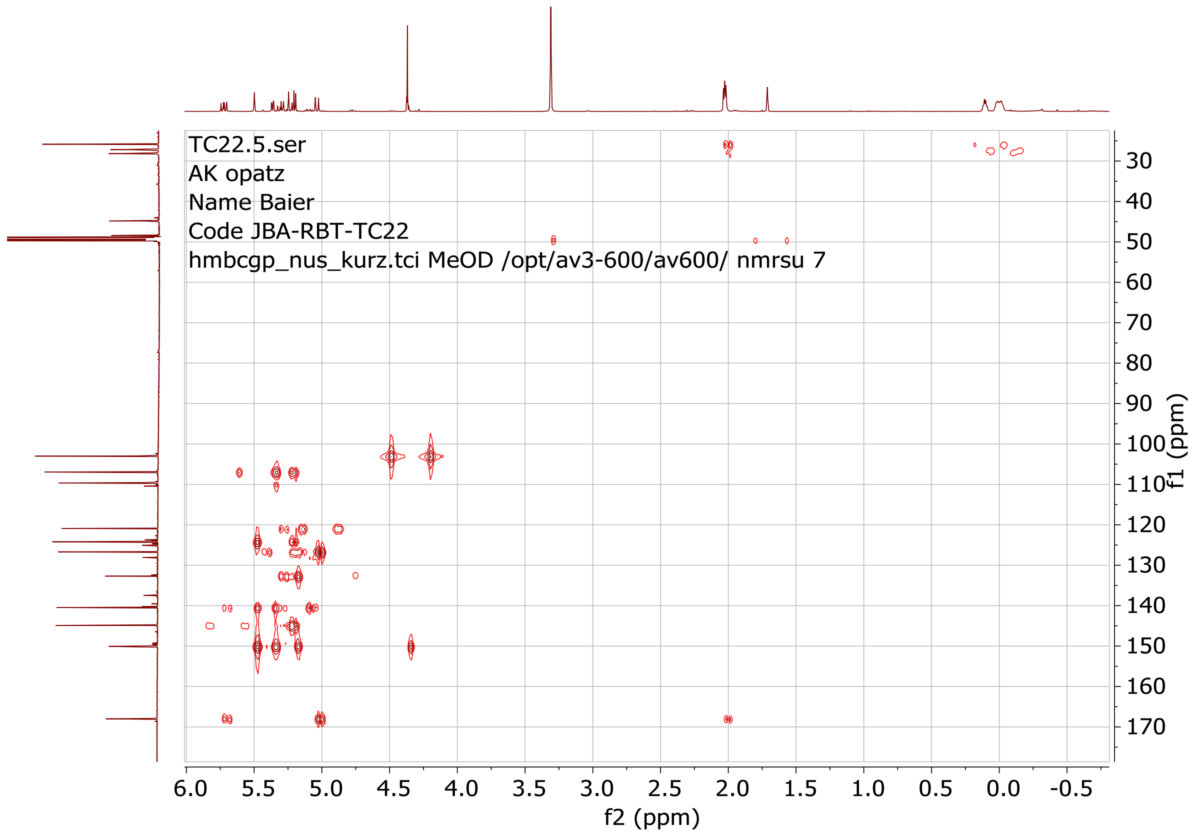

Figure S48. HMBC spectrum of compound **8**

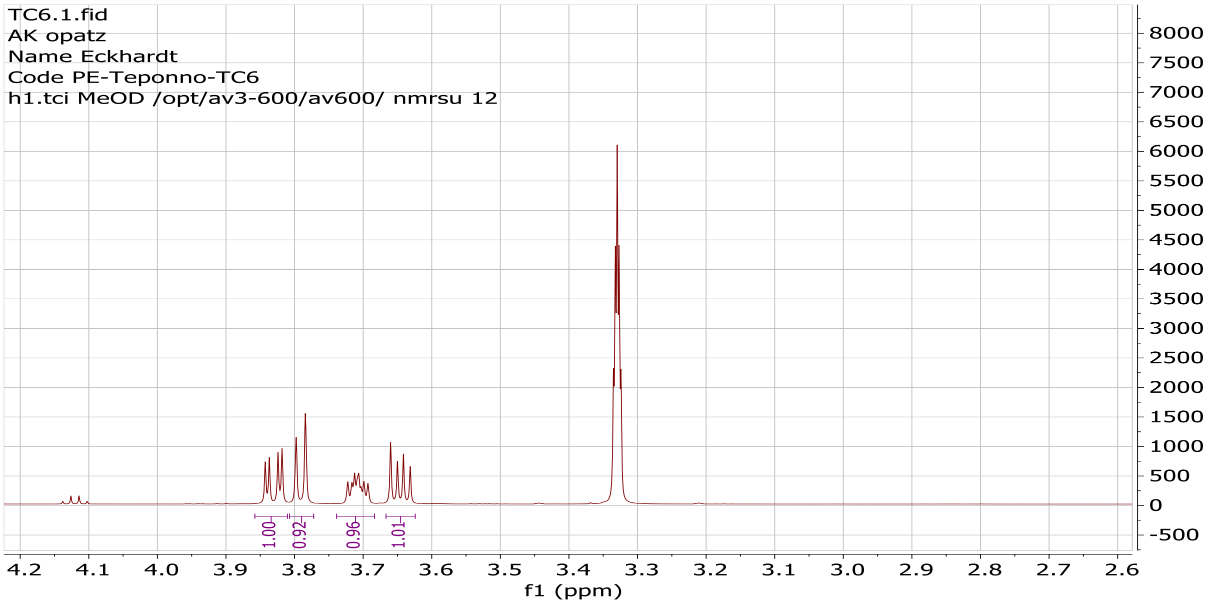

Figure S49. ^{1}H NMR spectrum (600 MHz, CD_3OD) of compound **9**

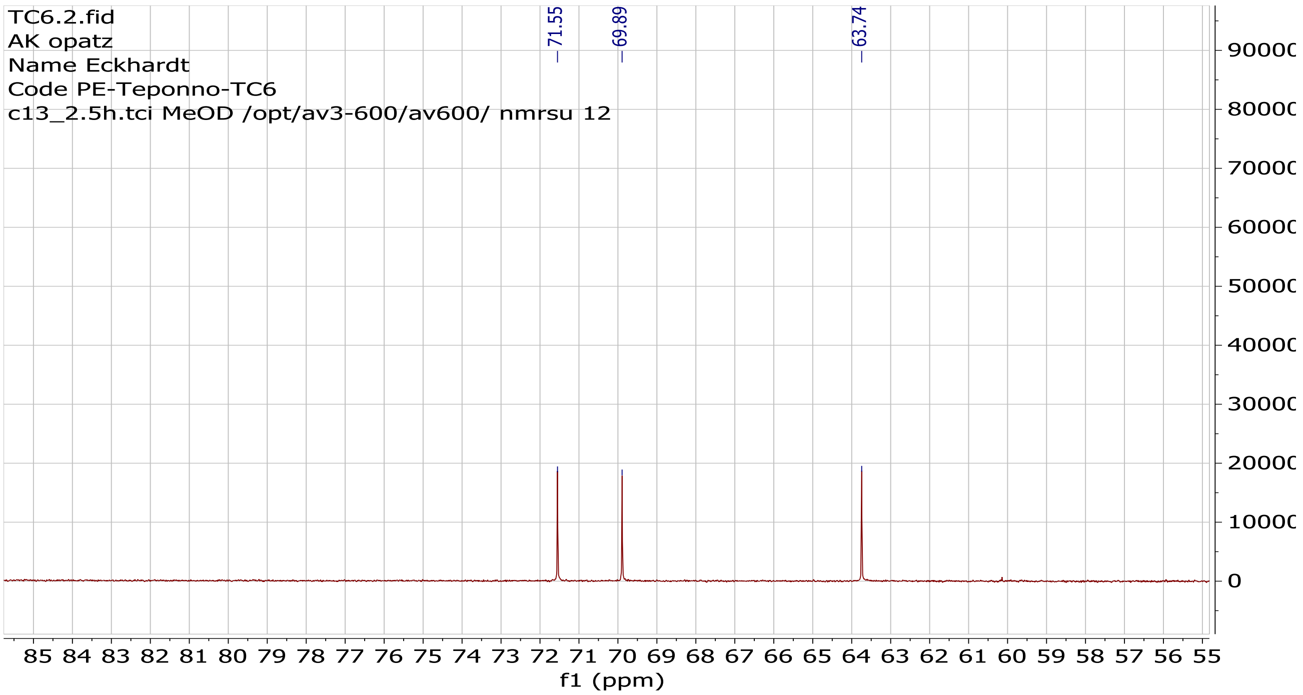

Figure S50. ^{13}C NMR spectrum (150 MHz, CD_3OD) of compound **9**

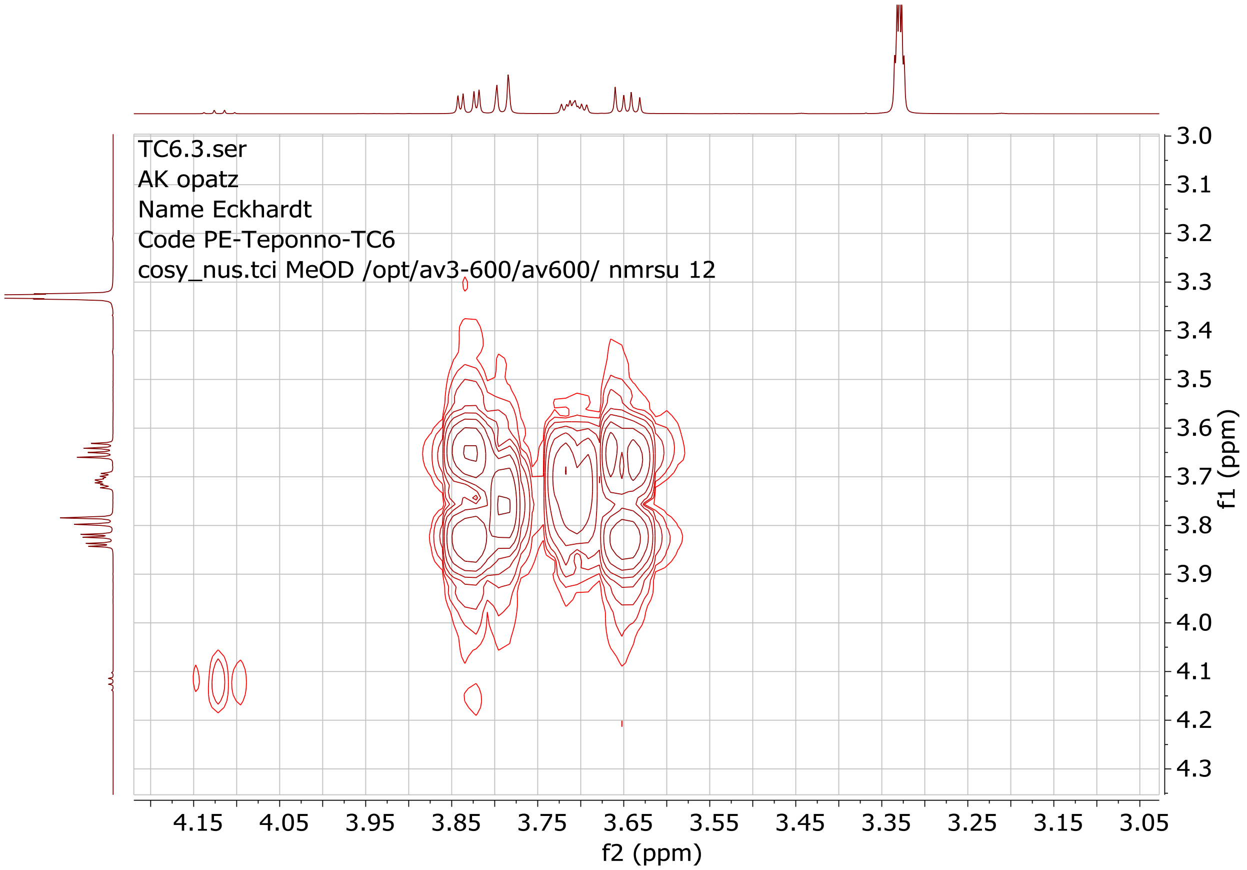

Figure S51. ^{1}H-^{1}H COSY spectrum of compound **9**

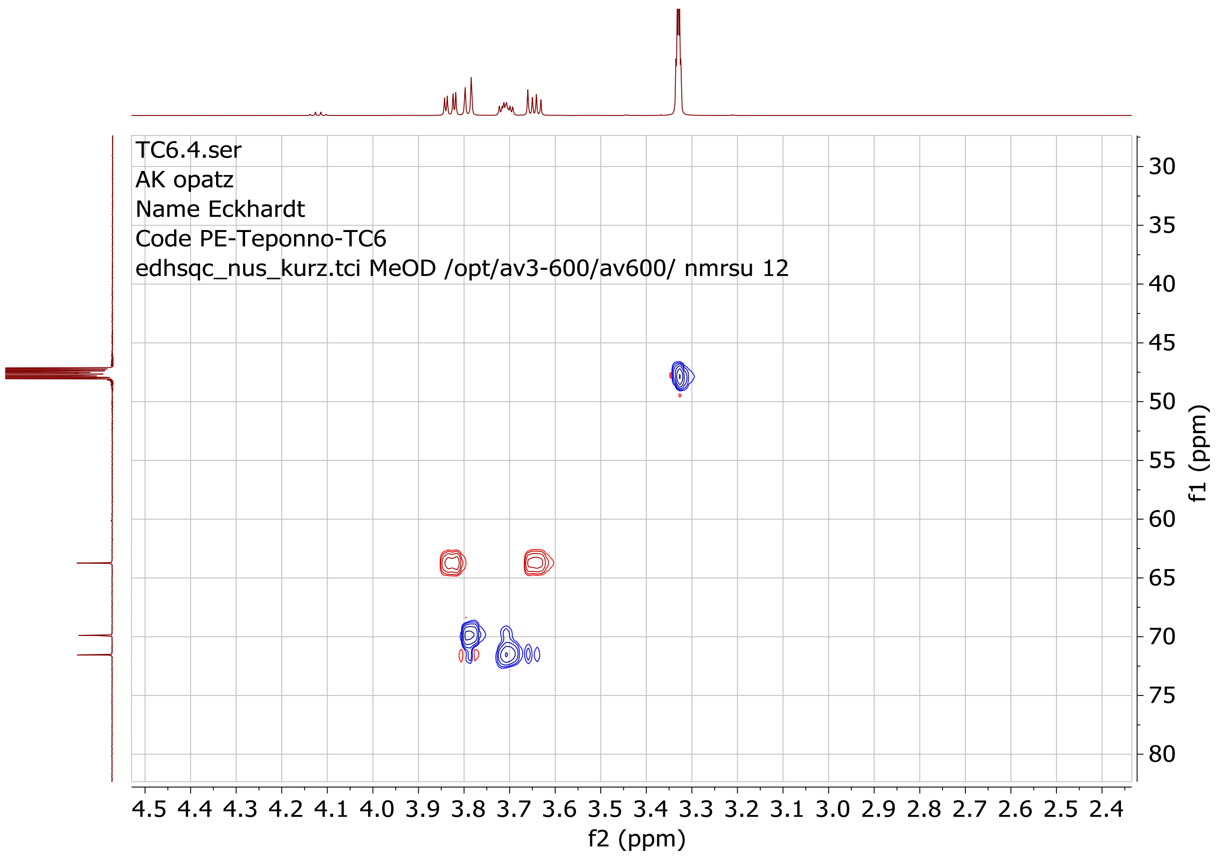

Figure S52. HSQC spectrum of compound **9**

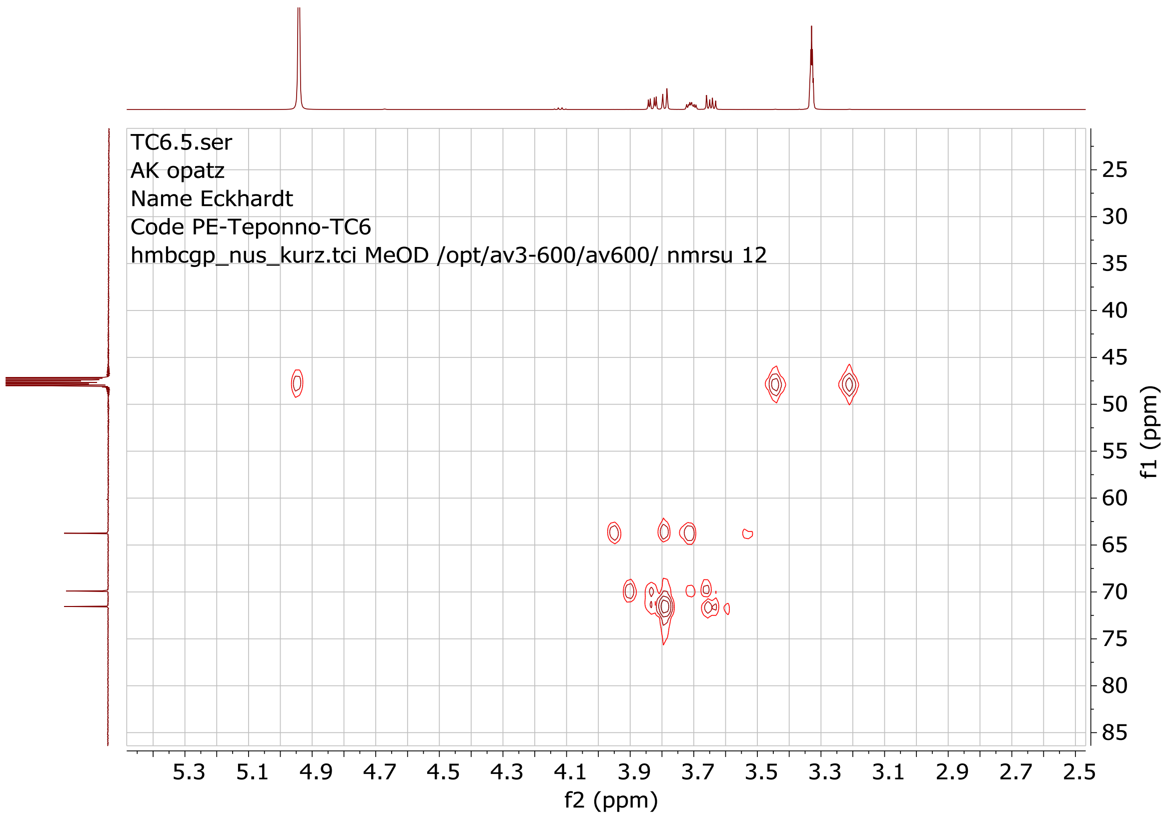

Figure S53. HMBC spectrum of compound **9**

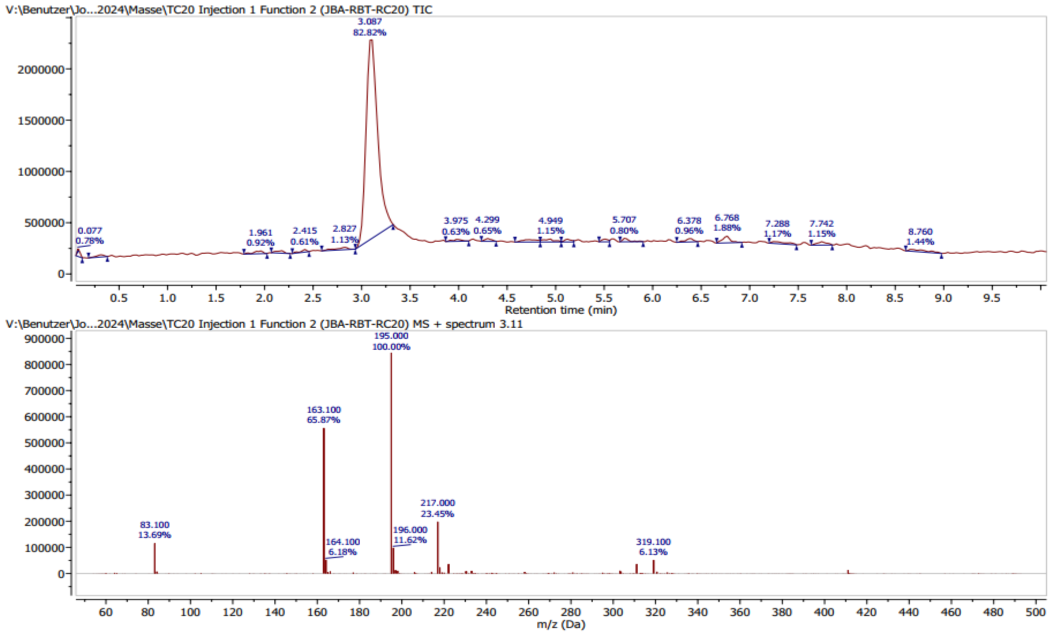

Figure S54. LCESIMS (+) of compound **10**

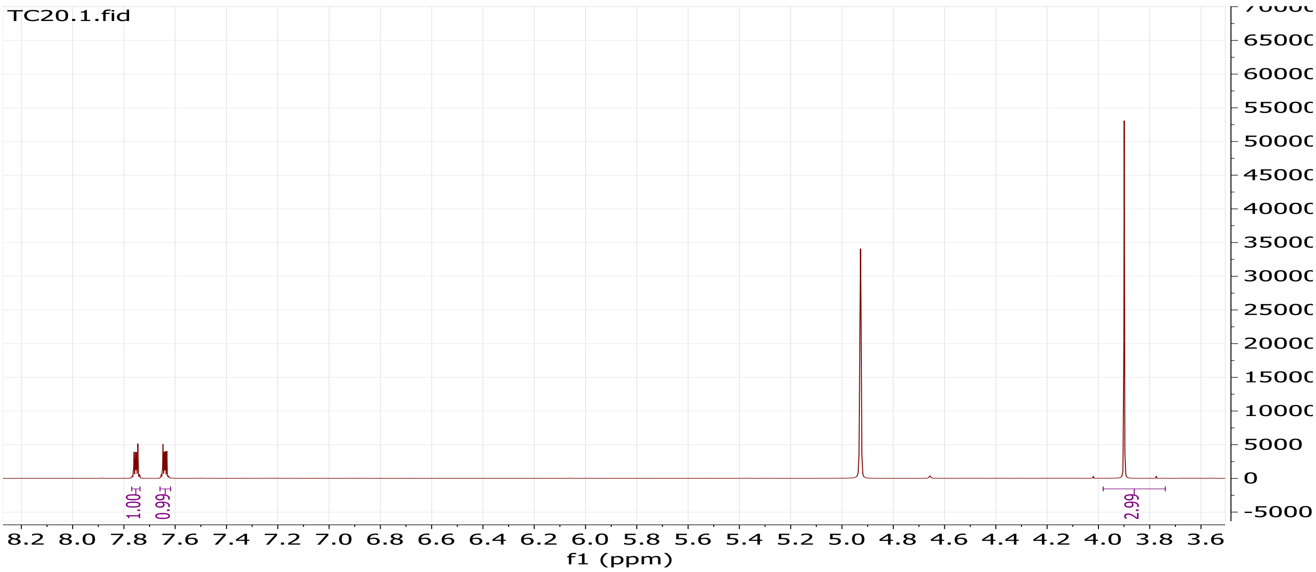

Figure S55. ^{1}H NMR spectrum (600 MHz, CD_3OD) of compound **10**

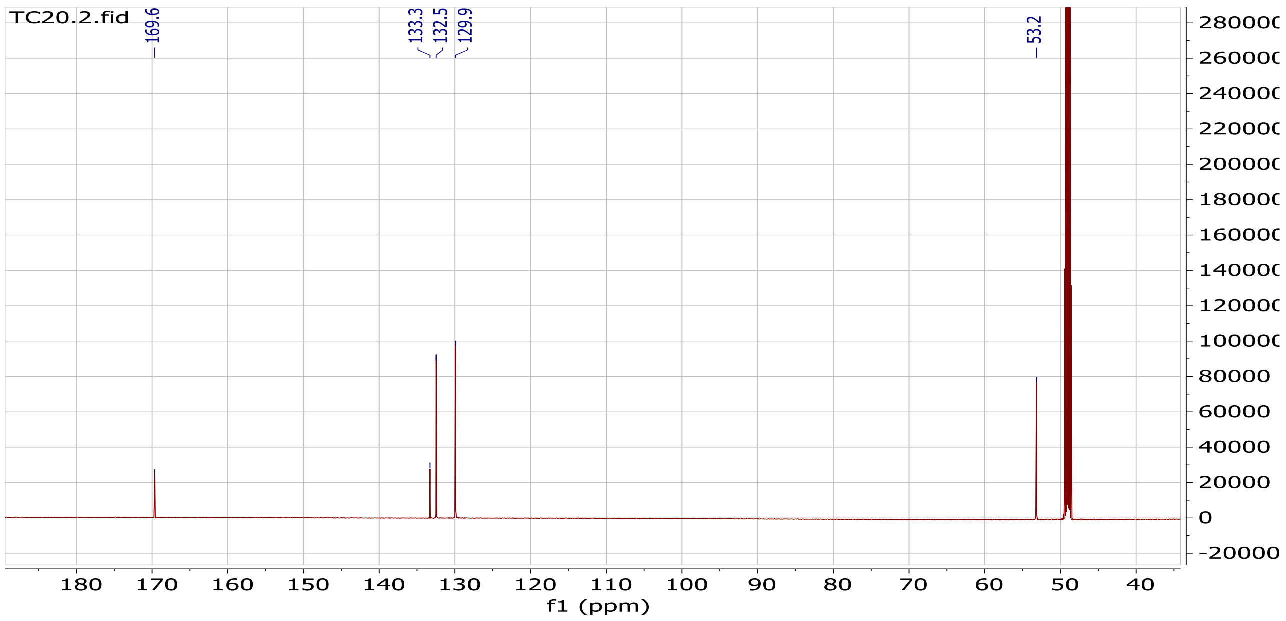

Figure S56. ^{13}C NMR spectrum (150 MHz, CD_3OD) of compound **10**

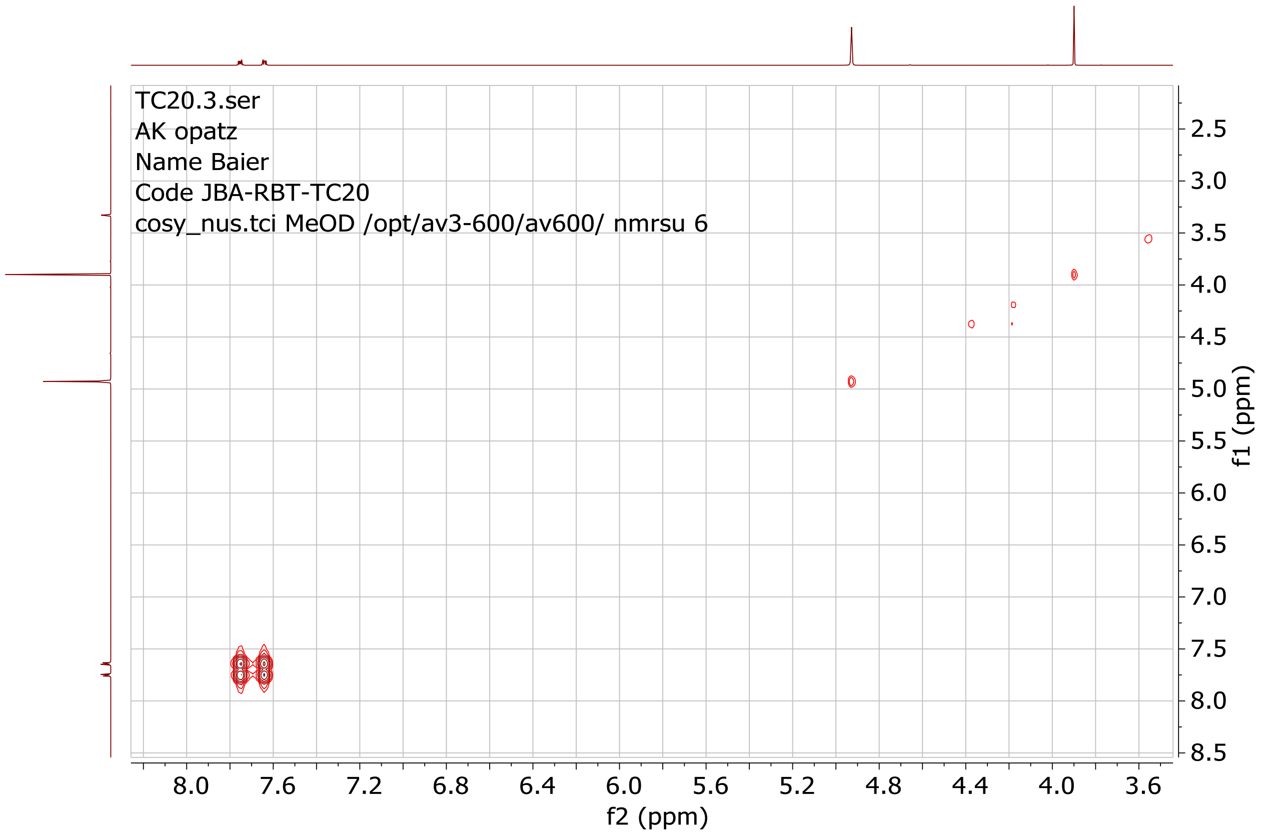

Figure S57. ^{1}H-^{1}H COSY spectrum of compound **10**

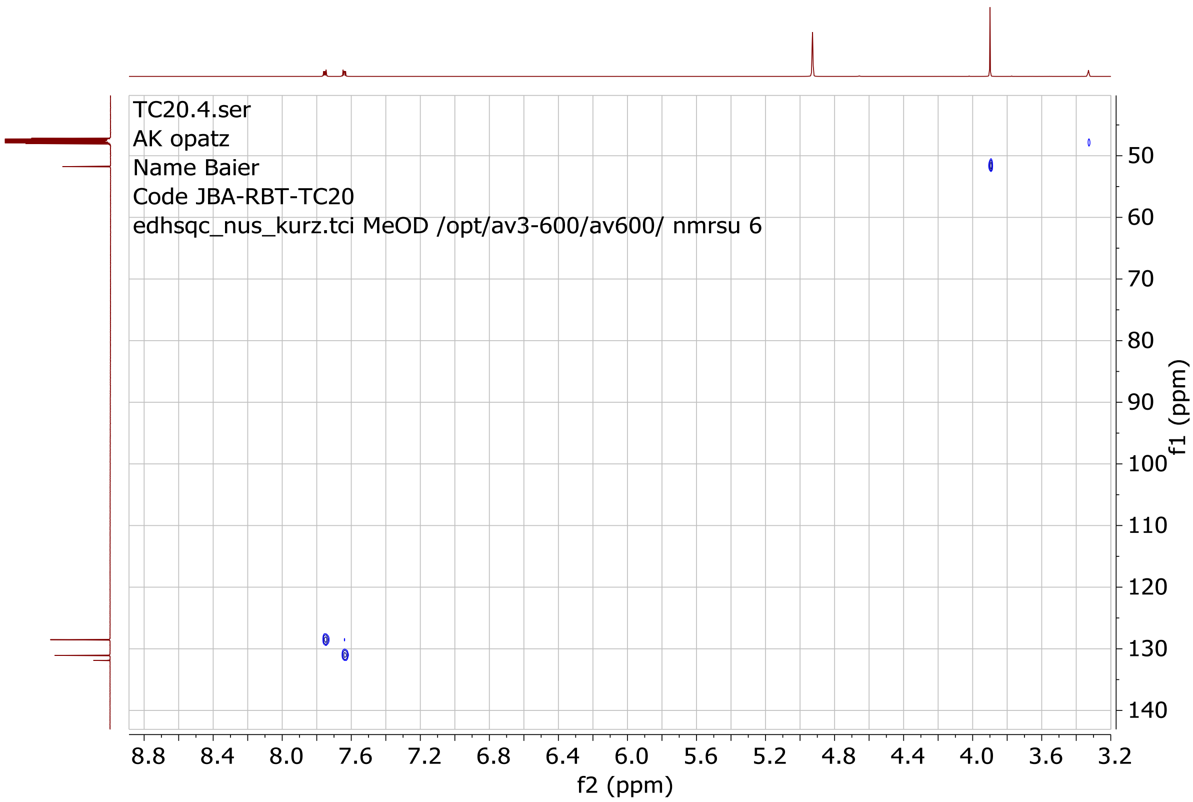

Figure S58. HSQC spectrum of compound **10**

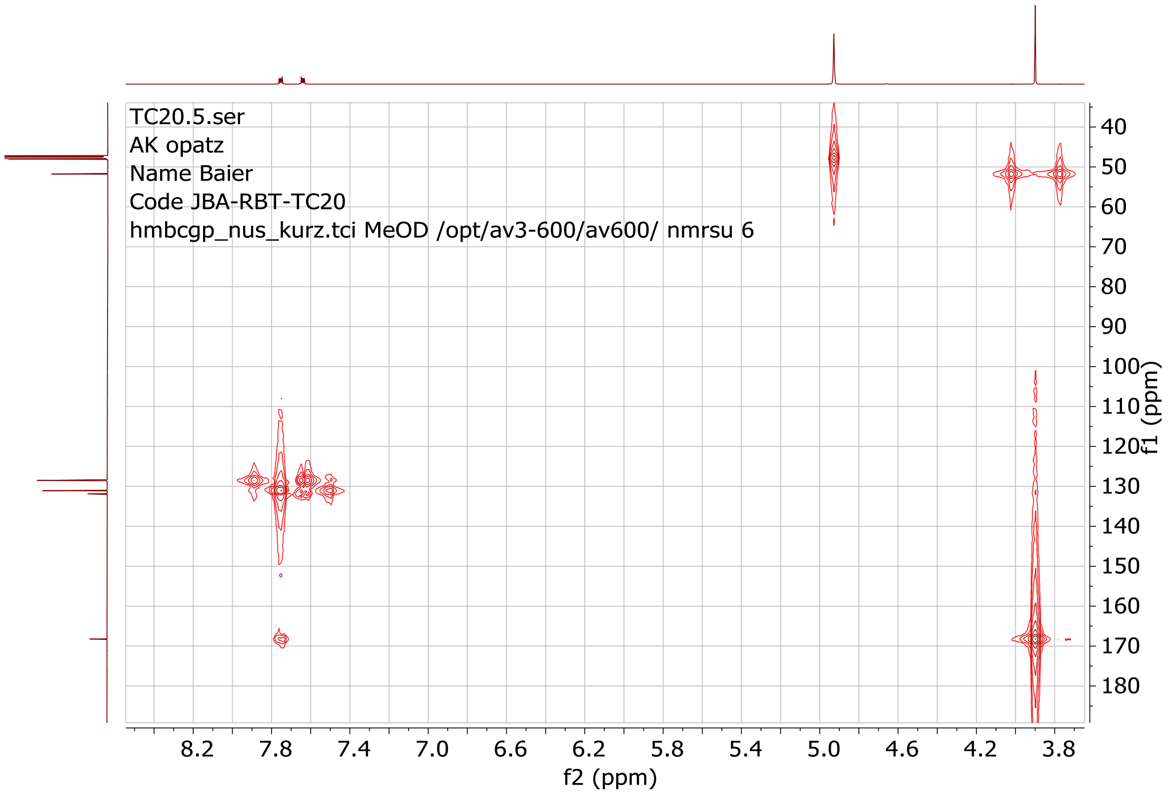

Figure S59. HMBC spectrum of compound **10**

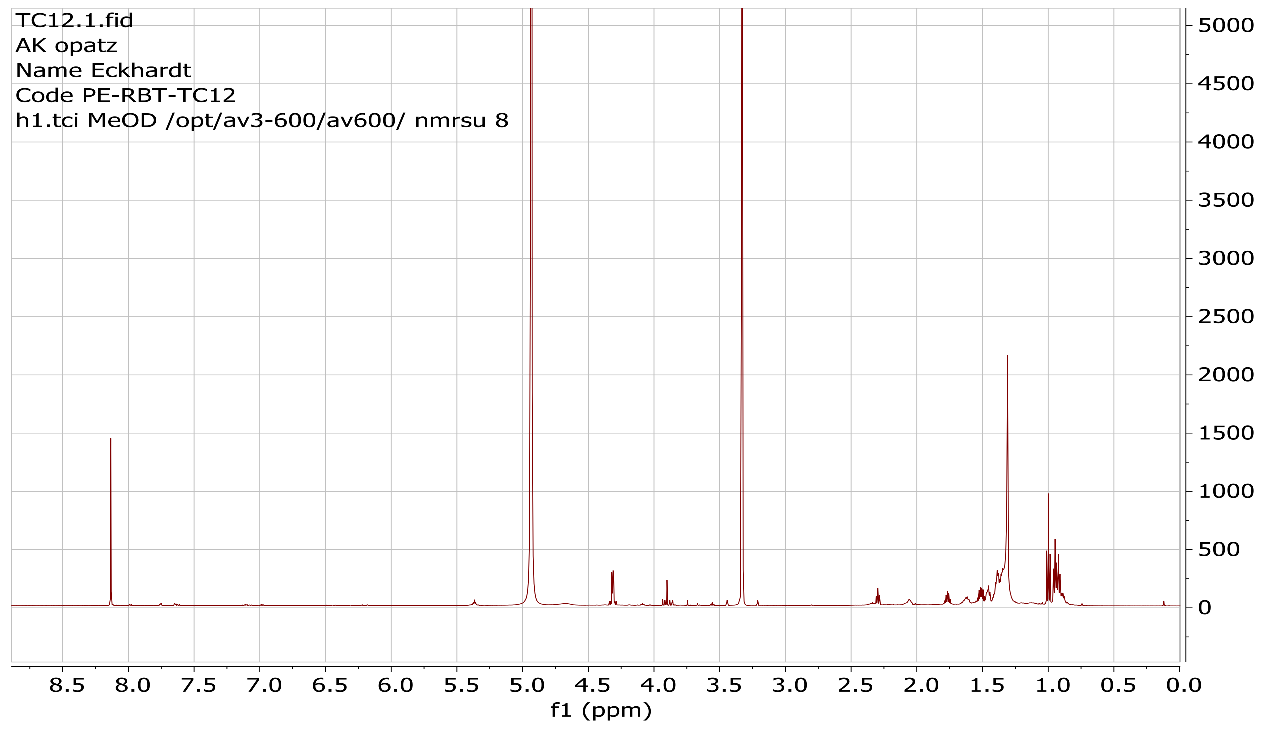

Figure S60. ^{1}H NMR spectrum (600 MHz, CD_3OD) of compound **11**

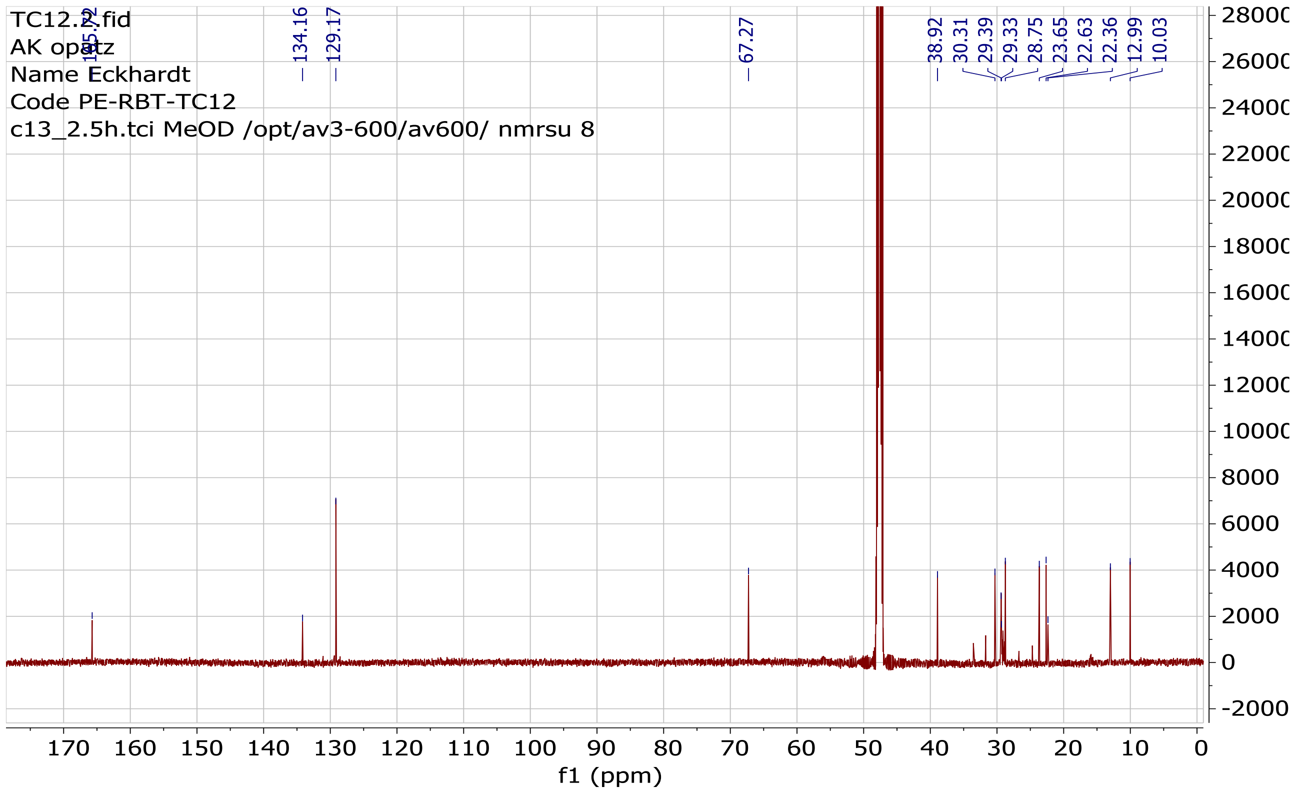

Figure S61. ^{13}C NMR spectrum (15 0 MHz, CD_3OD) of compound **11**

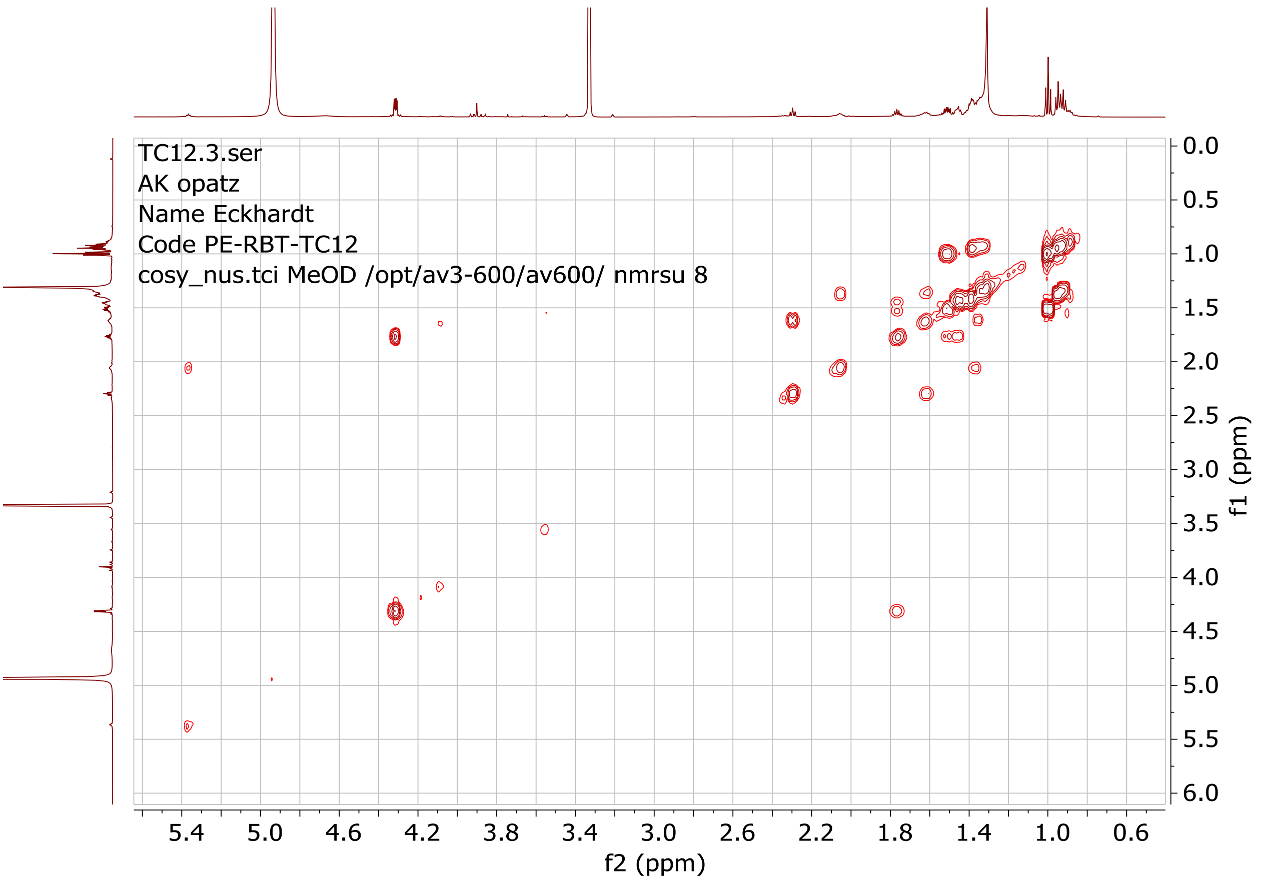

Figure S62. ^{1}H-^{1}H COSY spectrum of compound **11**

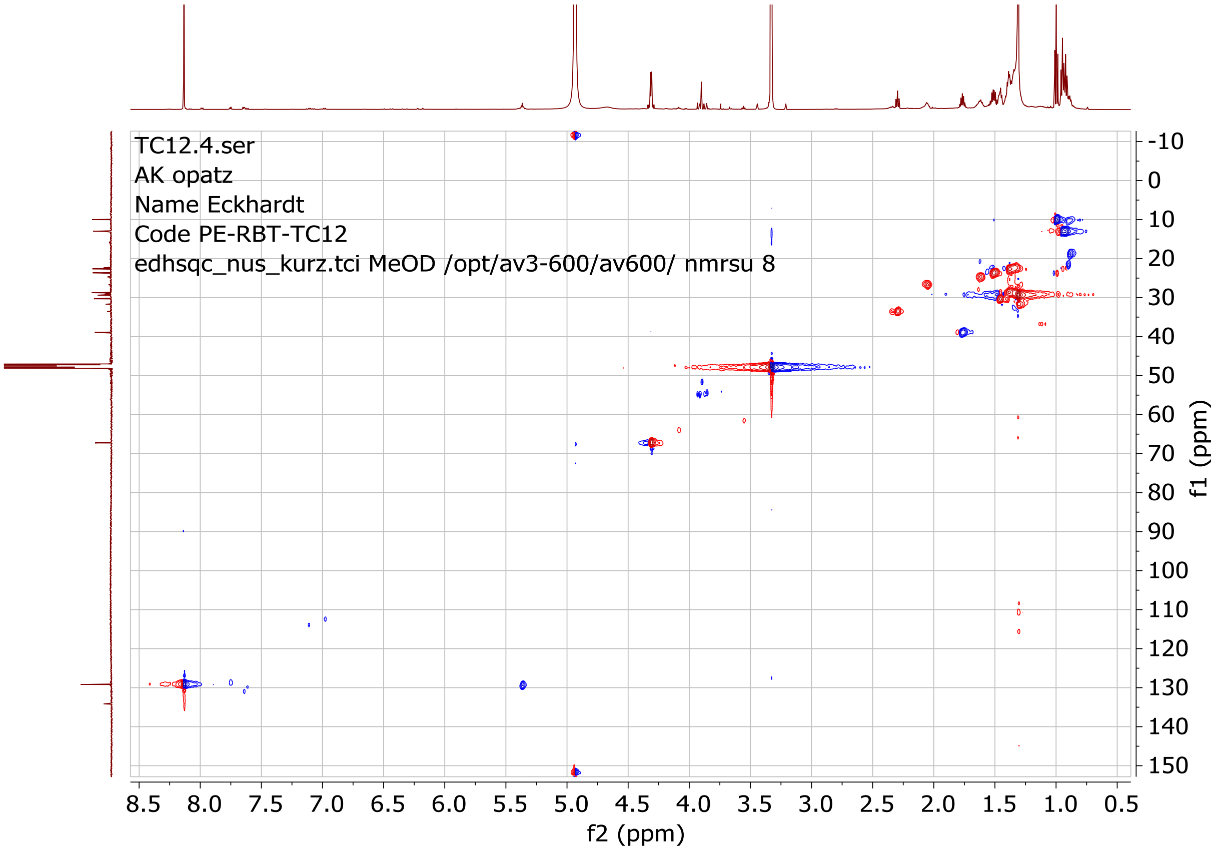

Figure S63. HSQC spectrum of compound **11**

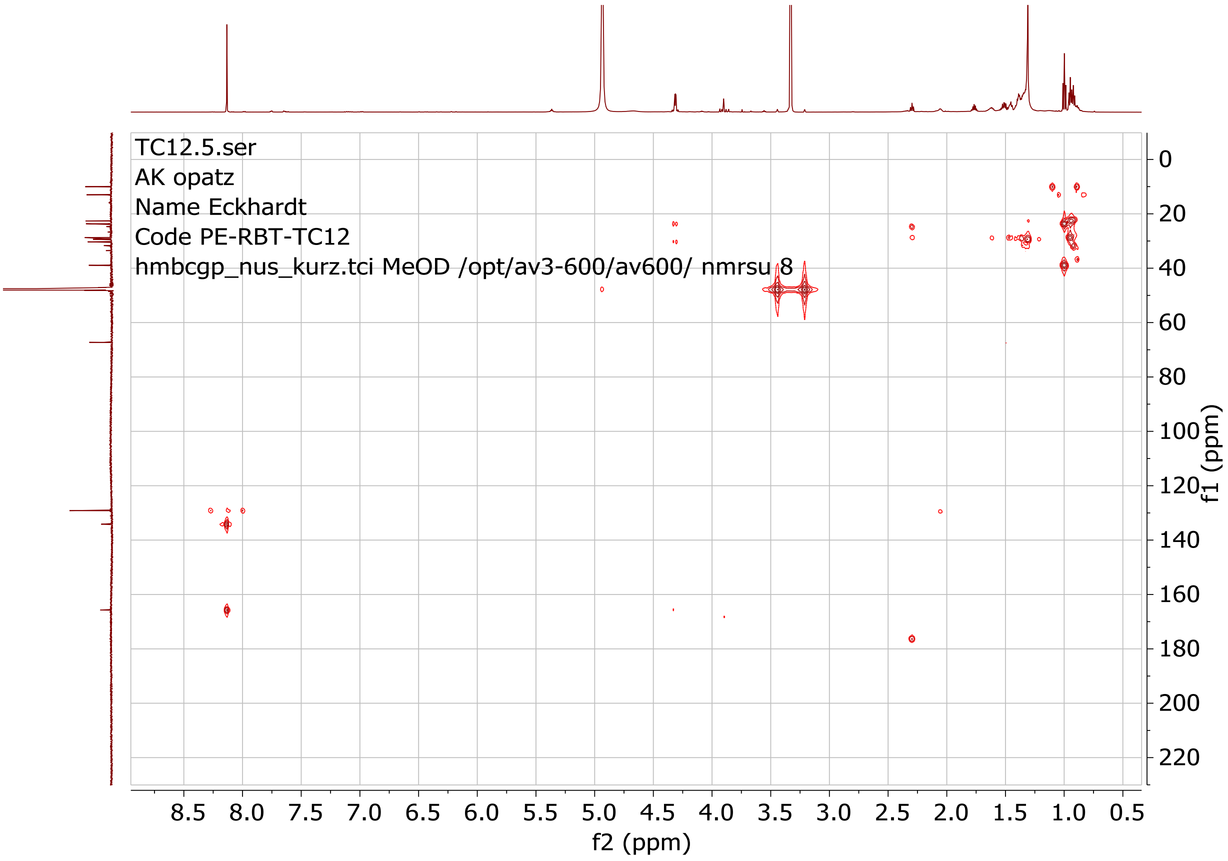

Figure S64. HMBC spectrum of compound **11**

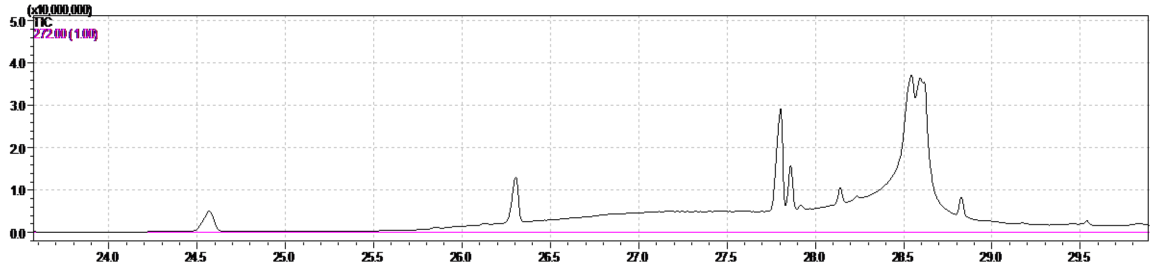

Figure S65. GC Chromatogram of Sub-fraction A of EtOH extract of *T. clypeatus*

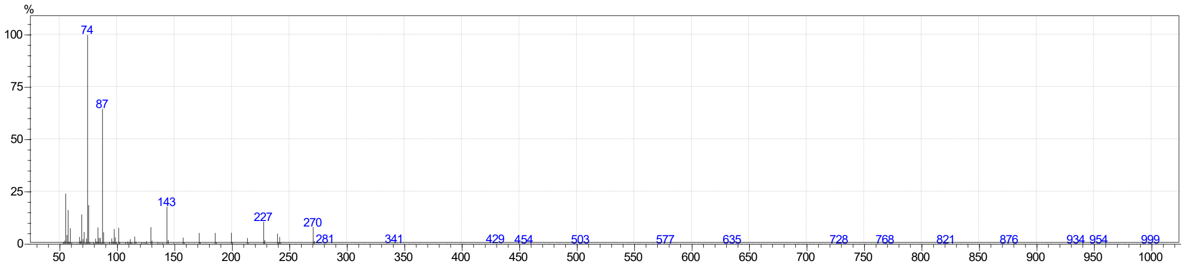

Figure S66. EI-MS of compound **12**

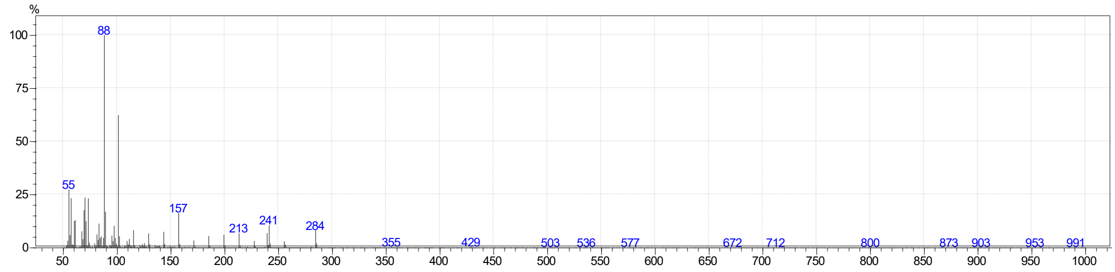

Figure S67. EI-MS of compound **13**

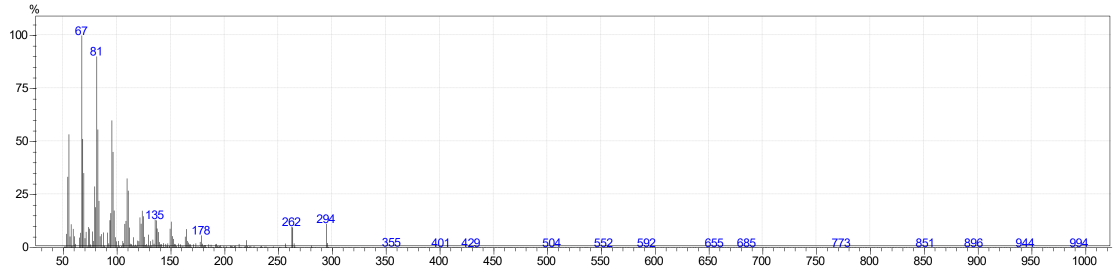

Figure S68. EI-MS of compound **14**

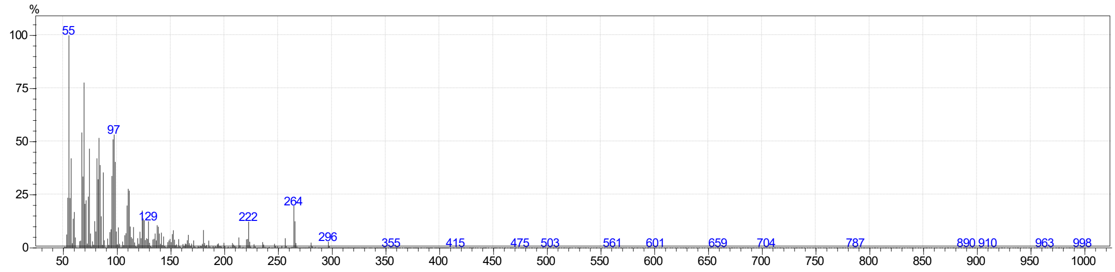

Figure S69. EI-MS of compound **15**

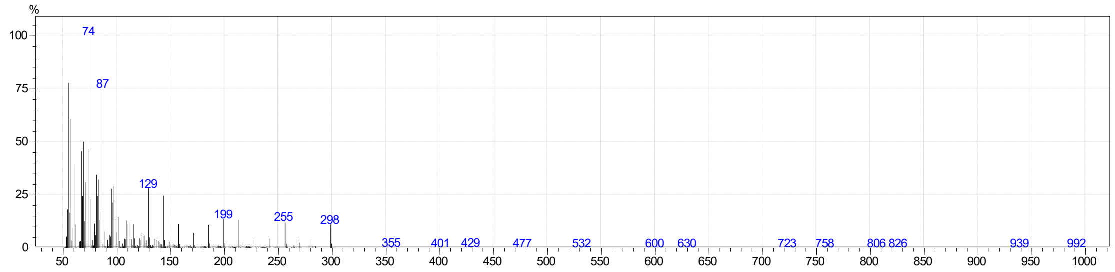

Figure S70. EI-MS of compound **16**

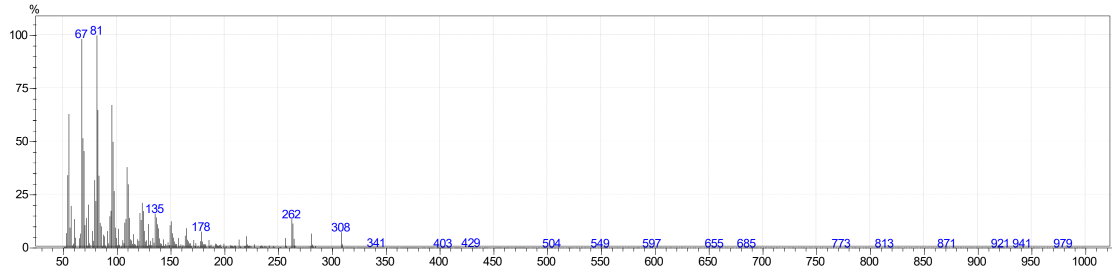

Figure S71. EI-MS of compound **17**

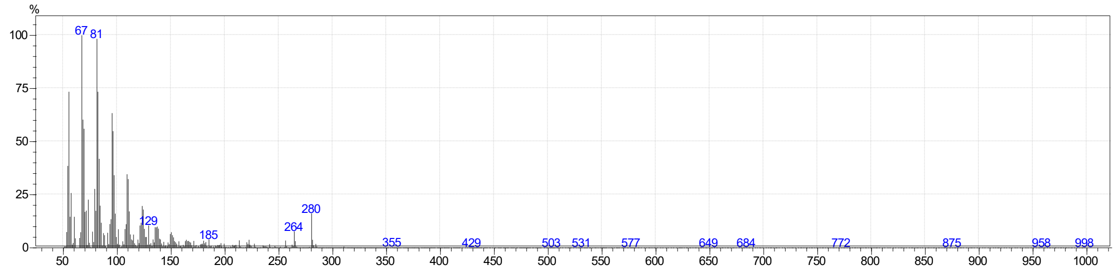

Figure S72. EI-MS of compound **18**

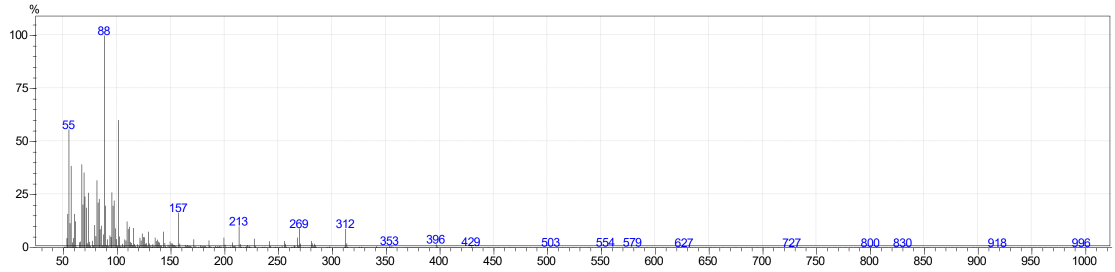

Figure S73. EI-MS of compound **19**
